# An 8-year-old girl with secondary histiocytic sarcoma with BRAF^V600^ mutation following T-cell acute lymphoblastic leukemia demonstrating stable disease for 3 years on dabrafenib and trametinib – a case report and literature review

**DOI:** 10.1186/s12887-025-05539-2

**Published:** 2025-03-08

**Authors:** Sue Lyn Tan, Betty Lee Sue Ho, Ting Ting Yew, Dahziela Yunus

**Affiliations:** 1 Department of Paediatrics, Faculty of Medicine and Health Sciences, Universiti Malaysia Sarawak, Jalan Datuk Muhammad Musa, Kota Samarahan, Sarawak, 94300 Malaysia; 2 Department of Paediatrics, Sarawak General Hospital, Jalan Hospital, Kuching, Sarawak, 93586 Malaysia; 3 Department of Radiology, Faculty of Medicine and Health Sciences, Universiti Malaysia Sarawak, Jalan Datuk Muhammad Musa, Kota Samarahan, Sarawak, 94300 Malaysia; 4 Department of Pathology, Queen Elizabeth Hospital, Kota Kinabalu Sabah, 88586 Malaysia

**Keywords:** Histiocytic sarcoma, BRAF mutation, MAPK-targeted therapy

## Abstract

**Background:**

Histiocytic sarcoma as a secondary malignancy following childhood leukemia is extremely uncommon with fewer than 20 cases reported worldwide. They often pose a diagnostic challenge and prognosis is dismal. There is a lack of well-established clinical treatment protocols owing to rarity of disease. Majority were managed with chemotherapy with variable outcomes.

**Case presentation:**

Herein we report a rare case of an 8-year-old girl with secondary BRAF^V600^-mutant histiocytic sarcoma following T-cell acute lymphoblastic leukemia. After poor disease control with salvage chemotherapy, she was treated with MAPK-targeted therapy with dabrafenib and trametinib. She demonstrated excellent response and remained in partial remission with no signs of disease progression 3 years later.

**Conclusions:**

There is yet to be consensus on the optimal management for this neoplasm. Description of our successful clinical experience highlights that investigation for BRAF mutations in histiocytic sarcoma is potentially advantageous. It also adds to the growing evidence that precision medicine may be a promising avenue to target this aggressive tumor and lays the foundation for future research.

**Supplementary Information:**

The online version contains supplementary material available at 10.1186/s12887-025-05539-2.

## Background

Histiocytic sarcoma (HS) developing as a secondary neoplasm is extraordinarily rare [1] in childhood and presents a diagnostic challenge. Dismal prognosis is expected due to rapid progression and poor response to therapy [2]. No optimal treatment has been defined and standardized [3]. Among the reported cases of secondary HS following acute lymphoblastic leukemia (ALL) in children (Table 1), treatment modalities employed include various regimens of chemotherapy [2, 4–9], thalidomide post-stem cell transplant [10], targeted therapy with monoclonal antibodies [11] and palliative care [9, 12]. Interestingly, a recent article discovered BRAF^V600E^ mutation in secondary HS of a child, who responded dramatically to MAPK-targeted therapy with combined BRAF inhibition (dabrafenib) and MEK inhibition (trametinib) [13]. We describe an 8-year-old girl with secondary BRAF^V600^-mutant HS who also demonstrated therapeutic benefit from this therapy, and provide an overview of the literature.

**Table 1:** Published cases of secondary histiocytic sarcoma following acute lymphoblastic leukemia in children

Case #	Year of publication	Author	Age/sex	Primary malignancy	Onset of secondary HS	HS location	Treatment	Outcome
1.	1996	Soslow et al.^4^	8y/ M	preB-ALL	10 m after ID	Paraspinous, bone, lung, liver, spleen	ETO, MEP	Died 3 m later
2.	1996	Soslow et al.^4^	6y/ M	ALL	20 m after ID	Bone, paravertebral, lung, liver	IFO, ETO, CBP	Alive 16 m later but developed new lesions
3.	2003	Wongchanchailert and Laosombat^5^	8y / F	preB-ALL	6 m after ID	Bone, extradural	CY, DNR, VCR, PRED	Died from sepsis following relapsed ALL
4.	2003	Dalle et al.^10^	4y/ M	T-ALL	9 m after SCT	Bone, BM, GIT, lung	Post-SCT: VIN, PRED, THA, DLI	Alive 33 m later
5.	2004	Feldman et al.^6^	14y/ M	preB-ALL	21 m after ID	BM, bone, spleen, kidney	VCR, CY, DNR, MTX, ETO	Not known
6.	2010	Castro et al.^7^	5y/ M	T-ALL	6 m after ID	Bone	Chemotherapy	Died
7.	2010	Castro et al.^7^	15y/ M	preB-ALL	3 m after ID	Soft tissue, bone, lung	Chemotherapy	Died
8.	2010	Castro et al.^7^	7y/ M	preB-ALL	6 m after ID	Bone, kidney	Chemotherapy, SCT	Alive at last follow up
9.	2010	Castro et al.^7^	3y/ M	T-ALL	16 m after ID	Liver, GIT	Chemotherapy	Died
10.	2011	Kumar et al.^8^	4y/ M	preB-ALL	1 m into maintenance	Bone, liver, spleen	DXM, CY, MTX, IFO, ARAC, ETO, RT	Died 1 year later
11.	2013	Karabova and Ilievova^9^	3y/ F	T-ALL	2 m into maintenance	GIT, LN, lung	DXM, CY, CLO, ETO, palliative care	Died 7 m later
12.	2014	Ganapule et al.^12^	4y/ M	T-ALL	18 m into maintenance	Bone, lung	Palliative care	Not known
13.	2015	Alten et al.^2^	6y/ M	T-ALL	15 m after ID	BM, liver, spleen, LN	DXM, ETO, ATG, BAS	Died 6 weeks later
14.	2015	Alten et al.^2^	10y/ M	T-ALL	12 m after ID	BM, skin, liver, spleen	DXM, VCR, MTX, ASP, ARAC, IDA, CY, NEL, SCT	Died 3 weeks after SCT from multiorgan failure
15.	2020	Venkataraman et el.^13^	1y/ M	T-ALL	4 m into maintenance	Bone	CLO, DXM, MAPK	Alive 14 m later
16.	2020	Valera et al.^11^	6y/ M	T-ALL	During maintenance	Skin, LN	CY, DOX, PRED, VCR, ALM, CLD, ARAC, SCT	Partial response, died after SCT
17.	2024	Our patient	8y/ F	T-ALL	2 m into maintenance	GIT, LN	IFO, CBP, ETO, MAPK	Alive 37 m later

ALL acute lymphoblastic leukemia, ALM alemtuzumab, ARAC cytosine arabinoside, ASP asparaginase, ATG anti-thymocyte globulin, BAS basiliximab, BM bone marrow, CBP carboplatin, CLD cladribine, CLO clofarabine, CY cyclophosphamide, DLI donor lymphocyte infusion, DNR daunorubicin, DOX doxorubicin, DXM dexamethasone, ETO etoposide, F female, GIT gastrointestinal tract, HS histiocytic sarcoma, ID initial diagnosis, IDA idarubicin, IFO ifosfamide, LN lymph node, M male, m months, MAPK mitogen-activated protein kinase targeted therapy with dabrafenib and trametinib, MEP methylprednisolone, MTX methotrexate, NEL nelarabine, preB precursor B-cell, PRED prednisolone, RT radiotherapy, SCT stem cell transplant, THA thalidomide, VCR vincristine, VIN vinblastine, y years

## Case presentation

A young female presented at 7 years 5 months of age with fever, hepatosplenomegaly and white blood cell count over 800,000/mm^3^. Bone marrow aspiration (BMA) revealed > 95% lymphoblasts and immunophenotyping confirmed T-acute lymphoblastic leukemia. No abnormalities were detected on cytogenetics and molecular mutation studies were negative for the 30 most common chromosomal translocations in acute leukemia. Cerebrospinal fluid examination was acellular. She was treated with the AIEOP-BFM ALL 2009 chemotherapy protocol. End-of-induction BMA reassessment showed complete remission with no minimal residual disease.

Approximately two months into maintenance therapy, the patient presented with three episodes of intussusception requiring laparotomy twice. A repeat bone marrow examination ruled out disease relapse. Initial histopathological examinations of the resected bowel and mesenteric lymph nodes were mostly consistent with a peripheral T-cell lymphoma, NOS. Four courses of CHOP (cyclophosphamide, doxorubicin, prednisolone and vincristine) chemotherapy were then given.

**Fig. 1:**
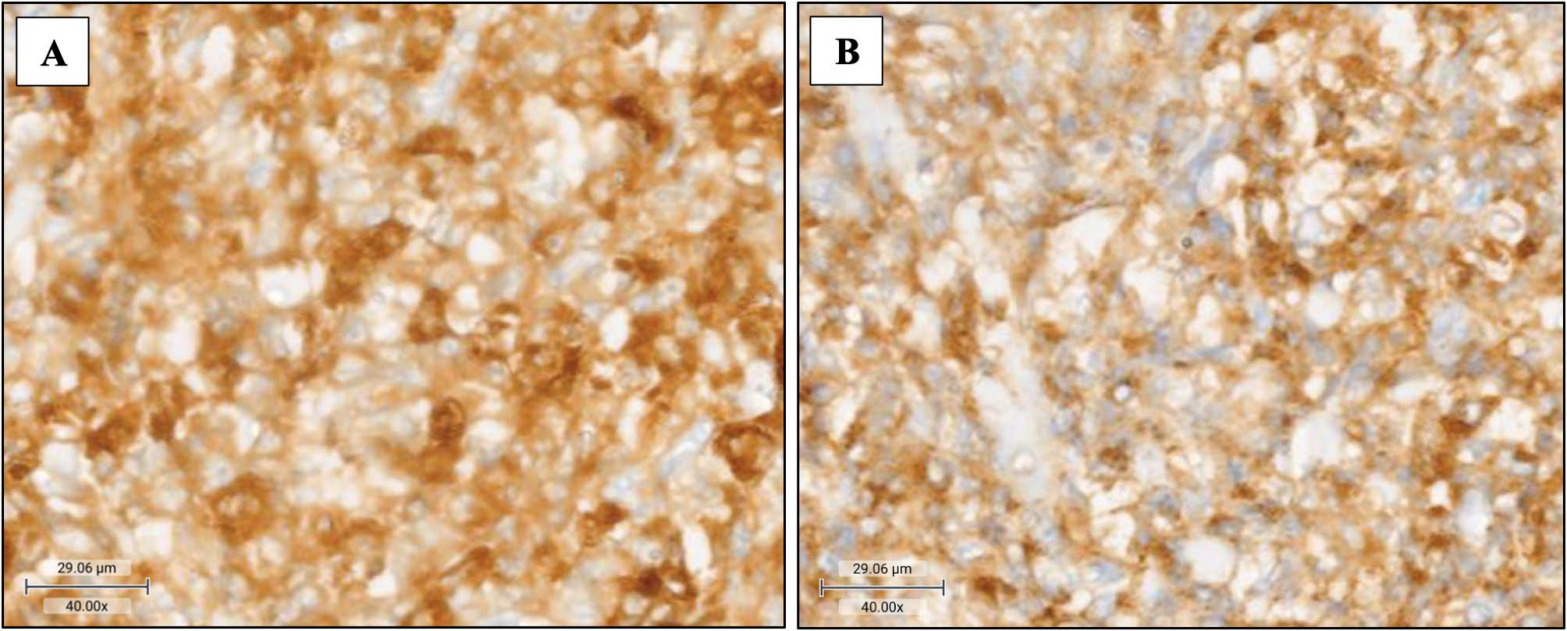
Immunohistochemical studies on gastrointestinal tissue The neoplastic cells showed histiocytic differentiation and stained strongly for (**A**) CD163 and (**B**) CD68 (original magnification 400x)

In view of mixed response to chemotherapy and following further consultation, additional immunohistochemical staining was performed on the previous gastrointestinal tissue and revealed that the neoplastic cells were positive for CD45+, CD4+, CD14+, CD68+, CD163 + and S100+, with weak and patchy expression of CD33 (Fig. 1). Negative staining was demonstrated for Langerhans cells, follicular dendritic cells, myeloid, B and T cells, epithelial, melanocytic and precursor markers (CD1a, CD23, MPO, CD117, CD56, CD30, CD20, CD79a, CD3, CD5, CD7, AE1/AE3, HMB45, TdT, CD34, CD99). Mutation in exon 15 of BRAF V600 (V600E, V600K and V600R) was detected, and the diagnosis was revised to histiocytic sarcoma. There is no family history of malignancy, and she does not exhibit any neurocutaneous or physical abnormalities to suggest an underlying cancer predisposition syndrome.

**Fig. 2:**
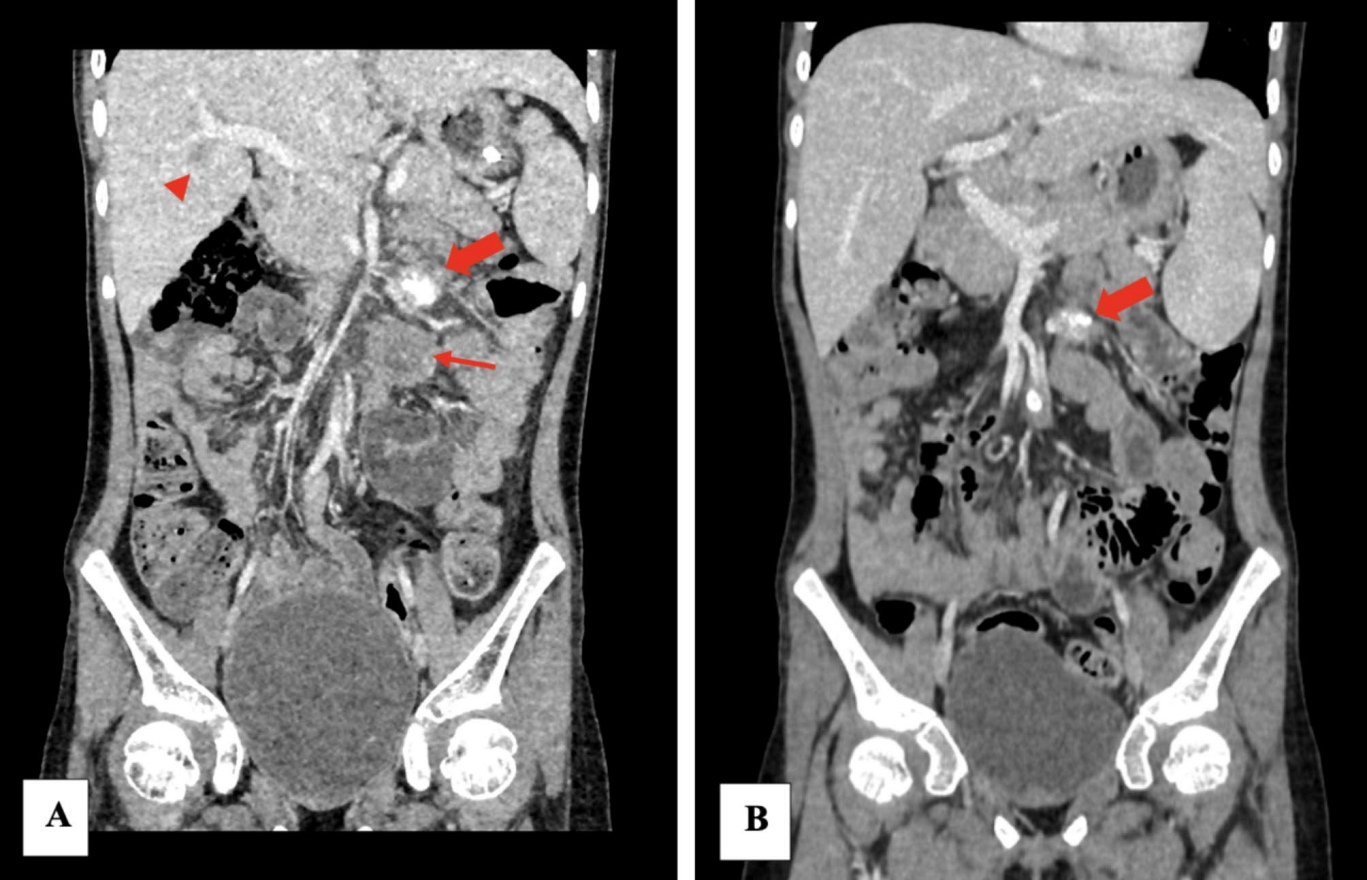
Coronal images of the child’s abdominal and pelvic CT scan **A** CT image 4 months prior to MAPK-targeted therapy revealed an enlarged calcified left mesenteric node (thick arrow), large left paraaortic node (thin arrow) and hypodense liver lesion (arrowhead). **B** CT image after 34 months of MAPK-targeted therapy showed a smaller left mesenteric node (thick arrow), smaller subcentimeter paraaortic nodes and resolution of liver lesion, indicating stable disease and no signs of disease progression

Salvage chemotherapy with ifosfamide, carboplatin and etoposide (ICE) was commenced in an effort to treat the histiocytic sarcoma. Despite five courses, reassessment CT scans of the abdomen and pelvis revealed that the disease was still progressing (Fig. 2A). She presented again with intussusception warranting a third laparotomy. In view of the presence of BRAF^V600^ mutation, MAPK-targeted therapy with dabrafenib and trametinib was instituted, with dabrafenib at 5.25 mg/kg/day divided into 2 doses and trametinib at 0.025 mg/kg/dose daily. She developed fever of unknown origin at the beginning of treatment. Trametinib dose was reduced temporarily but subsequently increased back to optimal dose once fever settled. She achieved a partial response and remained well more than 3 years later with no signs of disease progression (Fig. 2B). At time of writing (3 years 4 months of targeted therapy), she did not experience further pyrexia or any other side effects including dermatological, metabolic, hematological, neurological, gastrointestinal and musculoskeletal side effects.

## Discussion and conclusions

Second malignant neoplasms are well-recognized long-term health problems in individuals diagnosed with and treated for ALL at infancy, childhood and adolescence [11]. Histiocytosis, particularly HS, is very rarely described following ALL [11] with less than 20 cases reported in children to date [2, 4–13]. The overall survival of those with secondary HS was significantly lower at 11.8 months compared with 70 months for those with de novo HS as reported in a study of 23 adults and children [3]. Among the published cases of pediatric secondary HS as summarized in Table 1, there is an 82% male predominance and the mean age of affected children is 6.6 years. It most frequently presents during maintenance therapy for ALL but can occur as early as 3 months from initial diagnosis of ALL and up to 18 months after commencement of the maintenance chemotherapy. Commonly affected areas are extranodal in nature especially the bone (65% of cases), and to a lesser extent the lung, liver and spleen.

The diagnosis of HS can be extremely challenging owing to its rarity and paucity of clinical and genetic information on pediatric cases of secondary HS [11]. The unclear distinction between neoplastic and non-neoplastic proliferation of histiocytes, such as reactive histiocytosis [3] and histologic overlap with diverse mimics adds to the challenge. Patient #13, a 6-year-old boy, was initially diagnosed with hemophagocytic lymphohistiocytosis but died 10 days later despite highly intensive immunosuppressive treatment [2]. Histological evaluation of an abdominal lymph node (CD163+, CD68+, CD3-) removed during laparotomy ultimately revealed a diagnosis of HS [2]. The malignant cells of HS are typically CD163+, CD68+, lysosome + and CD1a- [14]. Prior to the additional immunohistochemical staining which eventually demonstrated CD163+, CD68 + and CD1a- in our patient, she was initially diagnosed and treated for peripheral T-cell lymphoma, stressing the importance of an awareness of HS and thorough clinical, morphological and immunohistochemical examinations.

Unsurprisingly, treatment modalities for secondary HS following ALL in children, are notably variable for this aggressive tumour, with differing outcomes. Castro et al. described four children with ALL preceding HS who were treated with chemotherapy (regimen not specified), three of whom succumbed to disease (#6, #7, #9), while one who also underwent stem cell transplantation was alive at last follow up (#8) [7]. Soslow et al. described an 8-year-old boy who received etoposide and methylprednisolone but died after 3 months (#1), and a 6-year-old boy who was treated with ifosfamide, etoposide and carboplatin but showed evidence of disease progression (#2) [4]. A 10-year-old boy with secondary HS received ALL relapse treatment (ALL-REZ BFM) and allogeneic stem cell transplantation despite absence of evidence of ALL relapse, due to lack of standardized treatment for HS and insufficient minimal residual disease clearance (#14) [2]. 

Additionally, even thalidomide which is generally only recommended as a last resort when all therapies fail was used in secondary HS following hematopoietic stem cell transplantation with favourable results (#4) [10]. Targeted therapy with monoclonal antibodies alemtuzumab was offered in a child with CD52 positivity in tumour cells but with only partial response, complicated with opportunistic infections, and he subsequently passed away (#16) [11]. A 4-year-old boy’s family opted for palliative care considering the dismal prognosis of the secondary HS he developed 1.5 years after maintenance therapy for T-cell ALL (#12) [12]. 

In adults, BRAF^V600^ mutations are commonly found in melanoma and thyroid cancers, and to a lesser degree other tumour types [15]. Mutation in BRAF at codon 600 causes constitutive activation of the MAPK pathway [16]. Successful inhibition of this pathway with BRAF/ MEK inhibitors results in clinically meaningful benefits [16]. This precision medicine approach has shown promising results in BRAF^V600^-mutated melanoma, non-small-cell lung carcinoma and thyroid cancer and is the standard-of-care option [17–19]. The NCI-MATCH trial involving more than 16 different tumour types with BRAF mutations including one patient with HS, reported that dabrafenib (BRAF inhibitor) and trametinib (MEK inhibitor) therapy resulted in responses in 38% of patients and showed a high rate of disease control, suggesting that BRAF/MEK inhibition may be a viable treatment strategy across the majority of BRAF^V600^-mutated cancers [15]. 

Langerhans cell histiocytosis, another histiocytic neoplasm, primarily affecting children, is well known to harbour BRAF mutations in more than 50% of cases [20]. Dabrafenib monotherapy or in combination with trametinib showed preliminary evidence of clinical efficacy in BRAF-mutant pediatric Langerhans cell histiocytosis, with a safety profile comparable to that observed in solid tumours in adults [20]. The first reported child with T-cell ALL developing secondary BRAF^V600E^-mutant HS, also treated with MAPK-targeted therapy had demonstrated therapeutic benefit and he remained in remission for 14 months (#15) [13]. Similarly, our patient with secondary BRAF^V600^-mutant HS responded dramatically to MAPK-targeted therapy and remains in partial remission with good quality of life and no evidence of disease progression for 3 years (#17).

In a study of histiocytic sarcoma in adults in Japan, only 6.1% (2 out of 33 patients) harboured BRAF^V600E^ mutation [21]. However moving forward, it may become essential to investigate for BRAF mutation in childhood histiocytic sarcoma. Insights into such genetic alterations have significant treatment implications as highly effective therapies with BRAF/ MEK inhibitors are now available. We are unable to conclude the origin of BRAF^V600^ mutation in our patient as it was not tested for in the initial ALL specimens. Given the rarity of secondary HS, compounded by the low overall rate of BRAF mutation in most tumour types [15], the feasibility of conducting disease-specific studies is limited. This highlights the value of our report as there is no consensus on optimal treatment yet, laying the foundation for future work on such malignancies.

HS as a secondary neoplasm following childhood leukemia is an exceptional and aggressive tumour which lacks uniform, well-established treatment protocols. For many rare cancers, it is challenging to develop clinical trials that recruit enough patients to show benefit from certain therapies, more so for strategies that target tumours with unique genetic mutations. We described a second child with BRAF^V600^-mutant secondary HS who showed therapeutic benefit from MAPK-targeted therapy, underlining the importance of investigation for BRAF mutations in HS and increasing the confidence in precision medicine to approach this highly malignant tumour. Further research is needed as we are still far from defining the standard treatment and the long-term effects of these newer avenues remain largely unknown.

## Electronic supplementary material

Below is the link to the electronic supplementary material.

Supplementary Material 1

Supplementary Material 2

Supplementary Material 3

Supplementary Material 4

Supplementary Material 5

Supplementary Material 6

Supplementary Material 7

Supplementary Material 8

Supplementary Material 9

Supplementary Material 10

Supplementary Material 11

## Data Availability

Data is provided within the manuscript and supplementary information files.

## Abbreviations

HS: Histiocytic sarcoma
ALL: Acute lymphoblastic leukemia
BMA: Bone marrow aspiration
