# Supplementary material for: An 8-year-old girl with secondary histiocytic sarcoma with BRAFV600 mutation following T-cell acute lymphoblastic leukemia demonstrating stable disease for 3 years on dabrafenib and trametinib – a case report and literature review
Source: BMC Pediatr. 2025 Mar 8;25:178. doi: 10.1186/s12887-025-05539-2 (PMC11889787; doi:10.1186/s12887-025-05539-2)
Supplement: Supplementary file 1 — Supplementary Material 1 [file 12887_2025_5539_MOESM1_ESM.pdf]

Med Pediatr Oncol 2003;40:51–53

BRIEF REPORT
True Histiocytic Lymphoma Following Acute Lymphoblastic Leukemia

Malai Wongchanchailert, MD* and Vichai Laosombat, MD

Key words: true histiocytic lymphoma; acute lymphoblastic leukemia; non-Hodgkin lymphoma

True histiocytic lymphoma (THL) is an uncommon type of non-Hodgkin lymphoma (NHL), the malignant cells of which originate from phagocytic histiocytes [1]. Using only histopathologic study, the diagnosis of THL has frequently been misinterpreted [2]. However, by the application of immunophenotypic technique for studying different cell lineages, many previously diagnosed THL have turned out to be Ki-1-positive anaplastic large cell lymphoma and high grade lymphoma of B or T cell origin [2,3]. The criteria generally used for diagnosis of THL is the reactivity of the malignant cells with the monocyte-macrophage markers and lack of reactivity with B- and T-cell markers [2–4]. Using such immunophenotypic criteria, the incidence of THL studied retrospectively is only 0.2–0.6% of all cases of NHL [3,5–8]. The common clinical feature of THL is a localized tumor that may or may not be disseminated. The usual organs of involvement are the skin, lymph nodes, gastrointestinal tract, and soft tissues [9–12]. Recently, the disease was reported to be a second tumor following completed treatment of lymphoblastic neoplasms [13]. Herein we report an additional patient with THL manifested during the maintenance chemotherapy for acute lymphoblastic leukemia (ALL).

She was an 8-year-old girl who presented with a 2-week history of limping, low grade fever, and bruises. Hepatosplenomegaly, generalized lymphadenopathy and petechiae and ecchymoses on trunk and extremities were found on physical examination. The complete blood count showed hemoglobin 91.0 gm/L, white blood cells 41.3×10^9/L, 69% of which were blast cells and platelet count 88.0×10^9/L. Lymphoblasts of L_2 (FAB) morphology with positive PAS staining were found in bone marrow aspirate. The immunophenotypes revealed 10 and 5% positive CD_8 and CD_2 markers respectively. She was treated with the modified Children Cancer Study Group protocol for ALL with good response. Six months later, she developed right hip pain. An osteolytic lesion was found in the right upper femur on plain film. The whole body bone scan showed multiple areas of increased radioactivity in the right upper femur, right acetabulum, distal left femur, the left seventh posterior rib, distal left humerus, and both pubic bones. Bone biopsy of the right femur was reported to be benign fibrous histiocytoma. She was continued on the maintenance phase of chemotherapy for ALL. Four months later, she developed pain in the back and weakness of the lower extremities. MRI of the whole spine showed an extradural mass at T8–10 level with cord compression. She went on T8–10 laminectomy and total tumor removal. The pathologic report was THL with positive CD 68 marker and negative myeloperoxidase, LCA, CD 45, CD 3, CD 45 RO, CD 20, and S100 markers (Fig. 1). The electron microscopic study confirmed the diagnosis of histiocytic lymphoma. She was treated with the CHOP protocol. The tumor partially responded. A few months later, the tumor recurred locally and additional tumor masses were found in the left distal humerus and ulna. The pathologic findings of the recurrent extradural and distal humeral masses were the same as the previous extradural mass. Nearly at the same time, blast cells were found in the peripheral blood. Bone marrow examination revealed a hypercellular marrow with 90% blast cells. These were negative for CD 68 staining and found to be lymphoblasts by electron microscopic study. The other immunophenotypes were not available. Reinduction chemotherapy included vincristine, doxorubicin, and cytarabine (etoposide was omitted because of anaphylactic reaction) together with local irradiation to the spine. Neutropenia ensued and she died of severe sepsis.

DISCUSSION

The diagnosis of ALL in our patient was straightforward by clinical, and laboratory findings. She responded

Department of Pediatrics, Faculty of Medicine, Prince of Songkhla University, Hat Yai, Songkhla, Thailand

*Correspondence to: Malai Wongchanchailert, MD, Department of Pediatrics, Faculty of Medicine, Prince of Songkhla University, Hat Yai, Songkhla, Thailand 90110. E-mail: Vprayong@ratree.psu.ac.th

Received 14 November 2000; Accepted 13 October 2001

© 2003 Wiley-Liss, Inc.
DOI 10.1002/mpo.10056

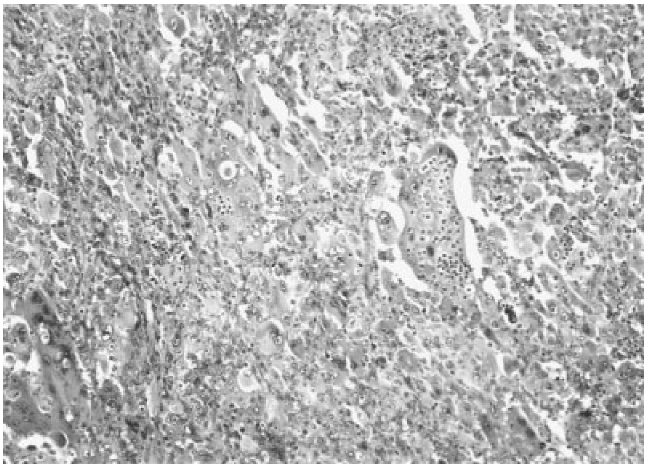

Fig. 1. Photomicrograph of the extradural mass reveals malignant histiocytes. Some of these cells show multinucleation with massive hemophagocytosis.

well to the usual chemotherapy for ALL and was in remission for 6 months before developing true histiocytic lymphoma (THL). The reactivity of CD 68 and the negative myeloperoxidase stain and B- and T-cell markers confirmed the diagnosis of THL. CD 68 has been suggested as a good marker among quite a number of immunophenotype markers used to identify histiocyte-macrophage cell lineage [14]. Bucsky et al. included negative gene rearrangement studies for immunoglobulin and T-cell receptor as additional criteria for diagnosis of THL [2]. However, it has been found that these gene rearrangements are not specific to lymphoid cell lineage and have been detected in other malignancies as well as THL [5,15].

The clinical manifestation of THL in our patient is quite similar to the two pediatric patients reported in the series of Soslow, et al. [13]. All of them are ALL patients in remission post chemotherapy and bone is the major organ involved as it is occasionally in primary THL [4,6,8]. Whether the ALL or the previous chemotherapy has any causative effect on the occurrence of THL is obscure. In view of the short interval between the treatment of ALL and the emergence of THL, chemotherapeutic agents seem unlikely to be the direct cause of the second malignancy. There are many reported cases of benign and malignant proliferation of histiocytic cells after the treatment of ALL and lymphoma, some of them associated with certain viral infections [16–22]. It could be that the histiocytes undergo reactive or malignant transformation effected by chemotherapeutically destroyed leukemic cell. Possibly the immunosuppressive state generated by the antileukemic chemotherapy plays a part. Otherwise, there may be a more subtle relationship between the lymphocytic malignancy and the malignant histiocytic disorder. The relationship would reflect the way in which histiocytes and lymphocytes interact in the immunologic response.

THL is an aggressive lymphoma. The treatment outcome for primary localized or disseminated THL is fairly good after combined treatment [4,8]. The chemotherapy commonly used is the CHOP protocol. There are too few cases of THL following lymphoblastic malignancy to assess for the optimal treatment. Etoposide based multiagent chemotherapy, generally used to treat acute monoblastic leukemia (AMoL), was suggested for THL since both THL and AMoL arise from histiomonocytic cell lineage [23]. However, both patients treated by Soslow et al. had only short remissions after such therapy. The overall prognosis for THL as second neoplasm remains dismal.

In conclusion, there appears to be more than a chance association between ALL post chemotherapy and THL. Study of more patients is needed to answer questions about the causal relationship between ALL and THL and effective treatment for this manifestation of THL.

ACKNOWLEDGMENTS

The authors are grateful to Associate Professor Sanya Sukpanichnan of the Department of Pathology, Siriraj Medical School, for his critical pathology review and comments.

REFERENCES

1. Cline MJ. Histiocytes and histiocytosis. Blood 1994;84:2840–2853.
2. Bucsky P, Favara B, Feller AC, et al. Malignant histiocytosis and large cell anaplastic (Ki-1) lymphoma in childhood: guidelines for differential diagnosis-report of the Histiocyte Society. Med Pediatr Oncol 1994;22:200–203.
3. Egeler RM, Schmitz L, Sonneveld P, et al. Malignant histiocytosis: a reassessment of cases formerly classified as histiocytic neoplasms and review of the literature. Med Pediatr Oncol 1995; 25:1–7.
4. Levine EG, Hanson CA, Jaszcz W, et al. True histiocytic lymphoma. Semin Oncol 1991;18:39–49.
5. Lukes RJ, Parker JW, Taylor CR, et al. Immunologic approach to non-Hodgkin's lymphoma and related leukemias. Analysis of the results of multiparameter studies of 425 cases. Semin Haematol 1978;15:322–351.
6. Hanson CA, Jaszcz W, Kersey JH, et al. True histiocytic lymphoma: histopathologic, immunophenotypic and genotypic analysis. Br J Haematol 1989;73:187–198.
7. Ralfkiaer E, Delsol G, O'Connor NTJ, et al. Malignant lymphomas of true histiocytic origin. A clinical, histological, immunophenotypic and genotypic study. J Pathol 1990;160:9–17.
8. Soria C, Orradre JL, Garcia-Almagro D, et al. True histiocytic lymphoma (monocytic sarcoma). Am J Dermatopathol 1992;14: 511–517.
9. Mirchandani L, Shah I, Palutke M, et al. True histiocytic lymphoma. A report of four cases. Cancer 1983;52:1911–1918.
10. Hsu SM, Ho YS, Hsu PL. Lymphomas of true histiocytic origin. Expression of different phenotypes in so-called true histiocytic lymphoma and malignant histiocytosis. Am J Pathol 1991;138: 1389–1404.

1096911x, 2003, 1, Downloaded from https://onlinelibrary.wiley.com/doi/10.1002/mpo.10056 by National Institutes Of Health Malaysia, Wiley Online Library on [30/09/2024]. See the Terms and Conditions (https://onlinelibrary.wiley.com/terms-and-conditions) on Wiley Online Library for rules of use; OA articles are governed by the applicable Creative Commons License

11. Milchgrub S, Kamel OW, Wiley E, et al. Malignant histiocytic neoplasms of the small intestine. Am J Surg Pathol 1992;16: 11–20.
12. Copie-Bergman C, Wotherspoon AC, Norton AJ, et al. True histiocytic lymphoma. A morphologic, immunohistochemical, and molecular genetic study of 13 cases. Am J Surg Pathol 1998; 22:1386–1392.
13. Soslow RA, Davis RE, Warnke RA, et al. True histiocytic lymphoma following therapy for lymphoblastic neoplasms. Blood 1996;87:5207–5212.
14. Knapp W, Rieber P, Dorken B, et al. Towards a better definition of human leucocyte surface molecules. Immunol Today 1989;10: 253–258.
15. Miettinen M, Fletcher CDM, Lasota J. True histiocytic lymphoma of small intestine. Analysis of two S-100 protein-positive cases with features of interdigitating reticulum cell sarcoma. Am J Clin Pathol 1993;100:285–292.
16. Karcher DS, Head DR, Mullins JD. Malignant histiocytosis occurring in patients with acute lymphoblastic leukemia. Cancer 1978;41:1967–1973.
17. Skoog DP, Feagler JR. T cell acute lymphoblastic leukemia terminating as malignant histiocytosis. Am J Med 1978;64:678–682.
18. Trubowitz S, Sobel H, David S. Null cell (non-T, non-B) acute lymphoblastic leukemia terminating as malignant histiocytosis. Am J Clin Pathol 1980;73:725–730.
19. Starkie CM, Kenny MW, Mann JR, et al. Histiocytic medullary reticulosis following acute lymphoblastic leukemia. Cancer 1981;47:537–544.
20. Yin JAL, Kumaran TO, Marsh GW, et al. Complete recovery of histiocytic medullary reticulosis-like syndrome in a child with acute lymphoblastic leukemia. Cancer 1983;51:200–202.
21. Liang D, Chu ML, Shih C. Reactive histiocytosis in acute lymphoblastic leukemia and non Hodgkin's lymphoma. Cancer 1986;58:1289–1294.
22. Takasaki N, Kaneko Y, Maseki N, et al. Hemophagocytic syndrome complicating T-cell acute lymphoblastic leukemia with a novel t (11;14) (p15; q11) chromosome translocation. Cancer 1987;59:424–428.
23. Elghetany MT. True histiocytic lymphoma: Is it an entity? Leukemia 1997;11:762–764.

1096911x, 2003, 1, Downloaded from https://onlinelibrary.wiley.com/doi/10.1002/mpo.10056 by National Institutes Of Health Malaysia, Wiley Online Library on [30/09/2024]. See the Terms and Conditions (https://onlinelibrary.wiley.com/terms-and-conditions) on Wiley Online Library for rules of use; OA articles are governed by the applicable Creative Commons License
