# Supplementary material for: An 8-year-old girl with secondary histiocytic sarcoma with BRAFV600 mutation following T-cell acute lymphoblastic leukemia demonstrating stable disease for 3 years on dabrafenib and trametinib – a case report and literature review
Source: BMC Pediatr. 2025 Mar 8;25:178. doi: 10.1186/s12887-025-05539-2 (PMC11889787; doi:10.1186/s12887-025-05539-2)
Supplement: Supplementary file 2 — Supplementary Material 2 [file 12887_2025_5539_MOESM2_ESM.pdf]

Pediatr Blood Cancer 2011;56:307–310

BRIEF REPORT
Pediatric Histiocytic Sarcoma Clonally Related to Precursor B-Cell Acute Lymphoblastic Leukemia With Homozygous Deletion of *CDKN2A* Encoding $p16^{INK4A}$

Riten Kumar, MD,[1] Shakila P. Khan, MD,[1] Divya-Devi Joshi, MD,[2] Gene R. Shaw, MD,[3] Rhett P. Ketterling, MD,[4] and Andrew L. Feldman, MD[4]*

Histiocytic sarcoma (HS) is a rare malignancy of tissue histiocytes with a dismal prognosis. We report a 4-year-old male who developed HS during maintenance chemotherapy for precursor B-cell acute lymphoblastic leukemia (pre-B ALL). Both tumors showed identical clonal immunoglobulin and T-cell receptor gene re-arrangement patterns, as well as homozygous deletion of the *CDKN2A* gene encoding $p16^{INK4A}$. These data suggest a clonal relationship between the two neoplasms despite their distinct lineages. Since *CDKN2A* deletion predisposes to development of HS in experimental models, the cytogenetic features of the patient's pre-B ALL may have predisposed to this change in lineage. Pediatr Blood Cancer 2011;56:307–310. © 2010 Wiley-Liss, Inc.

Key words: B-cell acute lymphoblastic leukemia; CDKN2A; histiocytic sarcoma; $p16^{INK4A}$; trans-differentiation

INTRODUCTION

Hematopoiesis traditionally has been viewed as unidirectional maturation of pluripotent stem cells into lineage-committed cells (lymphoid, myeloid, etc.). Accordingly, hematopoietic neoplasms are classified by the World Health Organization based on the lineage of the postulated normal cell from which the tumor derived [1]. Recent data, however, suggest a greater degree of lineage plasticity in both normal and malignant hematopoietic cells than previously believed. For example, it has been shown that murine B cells can be reprogramed into macrophages [2]. In humans, histiocytic sarcoma (HS)—the malignant counterpart of histiocytes/macrophages—has been reported in association with (and sometimes clonally related to) B-cell or T-cell acute lymphoblastic leukemia/lymphoma (ALL) [3–7].

Histiocytic sarcoma is a rare malignancy in all age groups, but is particularly uncommon in children. The largest series of HS, from the International Lymphoma Study Group, reported 18 cases with a median age of diagnosis of 46 years [8]. Clinical data was available for 12 patients and only 3 were in complete remission at last follow-up. A study from the Armed Forces Institute of Pathology reported an equally aggressive course with four of the five patients surviving only 2–15 months [9]. Pediatric data on HS is even more limited, but case reports usually indicate advanced presentation and refractoriness to conventional chemotherapy [10,11]. The etiology of HS is unknown, but HS has been associated with loss of the tumor suppressor, $p16^{INK4A}$, in both a murine model and in human tumors [12]. Interestingly, deletion of the *CDKN2A* locus encoding $p16^{INK4A}$ is also seen in both B-cell and T-cell ALL [13]. Here, we report a 4-year-old male who developed HS while receiving maintenance chemotherapy for pre-B ALL whose leukemic clone demonstrated a homozygous *CDKN2A* deletion. The subsequent HS showed identical immunoglobulin heavy chain (IgH) and T-cell receptor (TCR) gene rearrangement patterns, and demonstrated the same homozygous deletion of *CDKN2A*.

CASE REPORT

A 4 year-old-male was referred to Mayo Clinic, Rochester for a second opinion regarding a recent diagnosis of HS. The patient had been diagnosed with standard risk, pre-B ALL (Fig. 1A). Flow cytometry at diagnosis showed that the leukemic blasts expressed TdT, CD10, CD19, CD20, CD22, and CD34 (Fig. 1B,C). Bone marrow karyotype showed 46,XY,−9, i(9)(q10),del(17)(p11.2),+r[17]/46,XY[10]. Fluorescence in situ hybridization (FISH) revealed homozygous deletion of the *CDKN2A* locus at 9p21; the remainder of the B-ALL FISH panel was normal. The patient was enrolled on the Children's Oncology Group study AALL0331. Initial induction chemotherapy led to a complete response and subsequent chemotherapy was tolerated without major side effects. One month after starting maintenance chemotherapy, the patient developed diffuse, bilateral leg pain, which was initially attributed to avascular necrosis secondary to steroid therapy. His pain worsened and became localized to the right knee. A subsequent plain film demonstrated a 2.7 cm × 2.2 cm × 1.7 cm lytic lesion in the distal femoral metaphysis. Findings were confirmed on MRI, and the patient underwent an open biopsy of the femoral lesion and a posterior iliac crest bone marrow aspiration and biopsy.

The bone marrow biopsy showed no evidence of malignancy by morphologic evaluation or flow cytometry. Morphologic evaluation of the femoral lesion revealed large cells with markedly pleomorphic, multilobated nuclei and abundant, eosinophilic cytoplasm (Fig. 1D). Immunohistochemistry revealed that the tumor cells expressed the histiocytic markers CD4 (Fig. 1E), CD68 (Fig. 1F), CD45RO, and CD163. There was weak, focal positivity for S100. $p16^{INK4A}$ was negative (Fig. 1G), as were the B-lineage markers, CD19 and PAX5, and the lymphoblast marker, TdT. A diagnosis of

[1]Division of Pediatric Hematology-Oncology, Mayo Clinic, Rochester, Minnesota; [2]Division of Pediatric Hematology-Oncology, Torrance Hospital IPA, Torrance, California; [3]Division of Pathology, Marshfield Clinic, Marshfield, Wisconsin; [4]Department of Laboratory Medicine and Pathology, Mayo Clinic, Rochester, Minnesota

This case was presented at the 23rd Annual Meeting of the American Society of Pediatric Hematology/Oncology, April 7–10, 2010, Montreal, Quebec, Canada.

Conflict of interest: Nothing to declare.

*Correspondence to: Andrew L. Feldman, Department of Laboratory Medicine and Pathology, Mayo Clinic, 200 First Street SW, Rochester, MN 55905. E-mail: feldman.andrew@mayo.edu

Received 23 April 2010; Accepted 2 August 2010

© 2010 Wiley-Liss, Inc.
DOI 10.1002/pbc.22810
Published online 22 October 2010 in Wiley Online Library (wileyonlinelibrary.com).

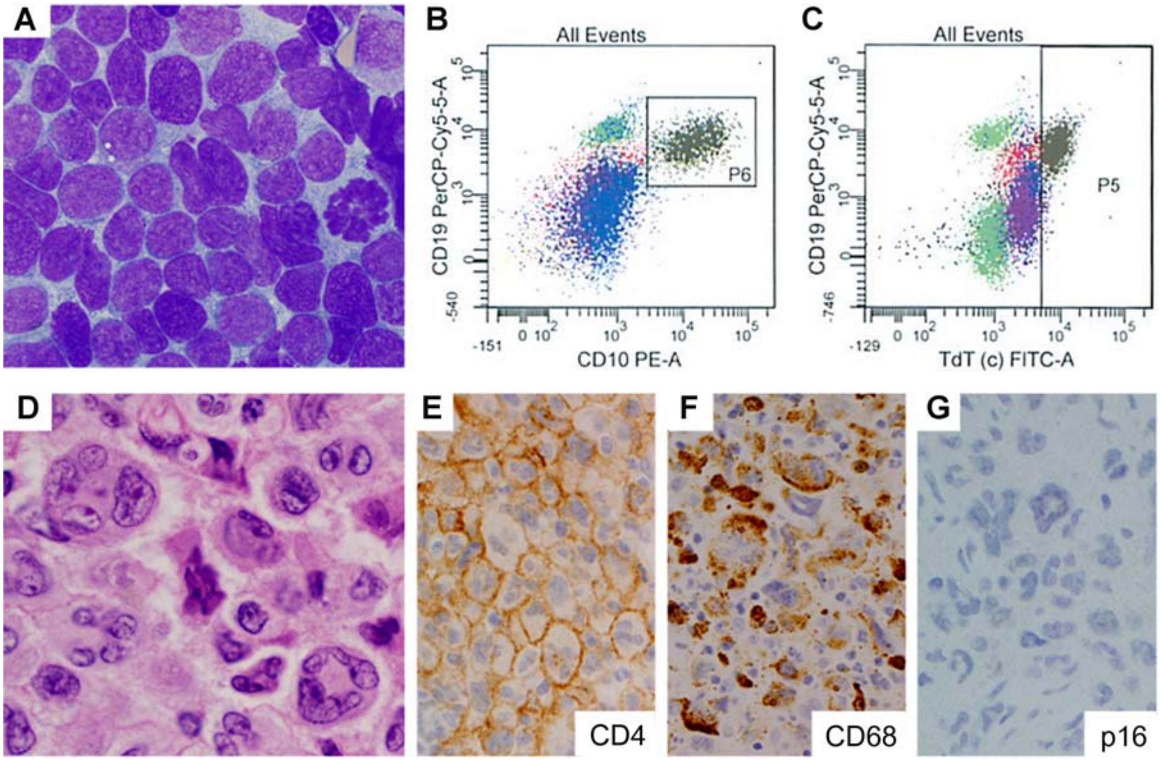

Fig. 1. Wright-Giemsa-stained smear from the original B-ALL shows lymphoblasts (bone marrow, 100×) (**A**). Flow cytometry histograms show the blast population (gray dots) to be positive for the B-cell marker CD19, the common ALL antigen (CALLA, CD10; P6) (**B**), and terminal deoxynucleotidyl transferase (TdT; P5) (**C**). Hematoxylin and eosin-stained section of the subsequent histiocytic sarcoma shows large, malignant cells with markedly pleomorphic nuclei (femur, 100×) (**D**). Immunohistochemical stains (40×) show the tumor cells are positive for the histiocytic markers CD4 (**E**) and CD68 (**F**), and negative for the tumor suppressor p16INK4 (**G**).

HS was made. PCR studies of the IgH (framework regions 3, 3a, and 3b) and TCR-gamma gene regions each showed identical, clonal bands in both the HS and the previous pre-B ALL (Fig. 2A,B). FISH showed homozygous deletion of *CDKN2A*, identical to the original pre-B ALL (Fig. 2C,D).

Leukemia therapy was held and the patient was started on chemotherapy as per AIEOP-ALCL 99. Pre-phase consisting of dexamethasone, cyclophosphamide, triple intrathecal and the first course of chemotherapy with dexamethasone, methotrexate, ifosfamide, cytarabine, and etoposide were completed. Two weeks after finishing the first course of chemotherapy, the patient developed marked hepatosplenomegaly, ascites, chest, and hip pain. Repeated imaging suggested involvement of bilateral iliac bones, ninth rib, and right mandible with interval progression of the right femoral lesion. After discussion with the parents, the patient was placed on palliative radiation therapy and died 1 year later.

DISCUSSION

Although secondary malignancies are relatively common in leukemia patients, few cases of HS have been reported [3–7]. Moreover, some cases reported as HS prior to the advent of routine immunohistochemistry likely were non-Hodgkin's lymphomas rather than true HS. Soslow et al. [3] described HS arising in three adults 10–20 months following therapy for ALL, and suggested this phenomenon might represent a distinct clinicopathologic entity. van

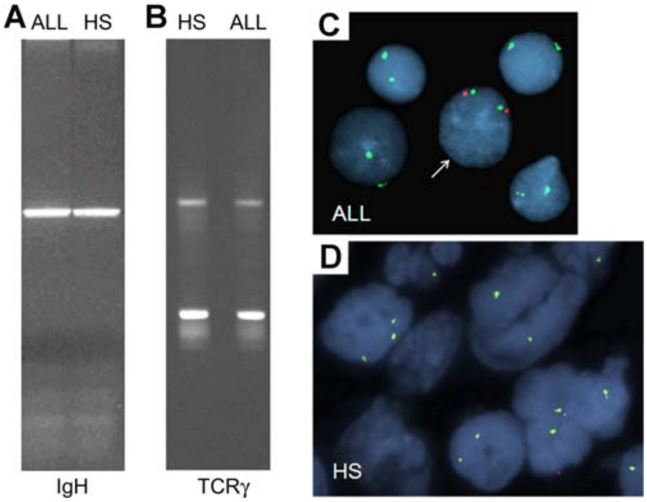

Fig. 2. PCR for immunoglobulin heavy chain (IgH; framework 3 shown) (**A**) and T-cell receptor gamma (TCRγ) (**B**) show identical clonal bands in the ALL and the HS. FISH was performed using probes for CDKN2A (encoding p16^{INK4A}) at 9p21 (red) and centromere 9 (green). The arrow shows a single normal cell in the ALL specimen (**C**). Red signals are absent in the other cells shown, as well as in the HS specimen (**D**), demonstrating homozygous CDKN2A deletion in both tumors.

der Kwast et al. [4] reported a histiocytic neoplasm with a clonal IgH gene rearrangement following therapy for T-lymphoblastic lymphoma. The same clone was identified in a post-treatment specimen from the original lymphoma. The authors suggested that both neoplastic populations probably were present at initial diagnosis, and while the lymphoma responded to chemotherapy, the malignant histiocytes were chemoresistant. Bouabdallah et al. [14] reported a 19-year-old male who developed HS 4 years after the therapy for B-ALL. Based on identical IgH gene rearrangements in both specimens, the authors suggested the B-ALL cells might act as progenitors that could trans-differentiate into a different lineage.

Whether due to two neoplasms with a common clonal origin, or one neoplasm trans-differentiating to another lineage, the report of similar cases suggests this phenomenon does indeed represent a distinct entity [5,6]. A recent series identified evidence of a clonal relationship in 7 of 15 histiocytic lesions following ALL [7]. Furthermore, this phenomenon is not restricted to lymphoblastic neoplasms, which might be hypothesized to retain some degree of lineage plasticity since they are tumors of precursor cells. Feldman et al. [15] reported clonally related histiocytic/dendritic cell neoplasms in eight patients with follicular lymphoma, a neoplasm of mature B cells.

What remains unclear is why rare patients with ALL (or other lymphoid neoplasms) develop clonally related histiocytic tumors, while most do not? Studies in mice suggest that down-regulating expression of the B-cell transcription factor, PAX5, can reprogram B cells to either macrophages or uncommitted progenitor cells [2,16]. As in the present case, histiocytic tumors following B-cell neoplasms have been reported to lack PAX5 expression [15], but the mechanism for down-regulation has not been established; furthermore, down-regulation of PAX5 would not explain the occurrence of histiocytic lesions following T-ALL [7]. Another possibility is suggested by a study of double-mutant mice lacking both copies of *CDKN2A* and one copy of the tumor suppressor gene, *PTEN* [12]. These mice developed both lymphoblastic lymphomas and HSs, which the authors postulated was associated with abnormal regulation of myelolymphoid (myeloid, T-cell, and B-cell) progenitor (p-MTB) cells. In addition, they demonstrated loss of $p16^{INK4A}$ expression in 4/10 human HS tumors. In our case, homozygous *CDKN2A* deletion was present in both the pre-B ALL and HS specimens, suggesting the possibility that loss of $p16^{INK4A}$ expression might be one factor in ALL that predisposes to development of HS. It would have been of interest to test PTEN in our case, but this was not technically feasible on the material available. It is interesting to note, however, that a case of HS following T-cell ALL has been reported in which both neoplasms showed homozygous deletion of *CDKN2A* [7]. If the murine model [12] holds true for humans, perhaps the fundamental defect in patients with both HS and ALL is an abnormal myelolymphoid precursor with loss of critical tumor suppressor genes such as *CDKN2A*.

The rarity of HS makes it difficult to formulate evidence-based treatment guidelines. Surgery, radiation therapy, chemotherapy, stem cell transplant, and a combination of these modalities have been used with poor results [8,9]. Among 14 cases reviewed by Hornick et al. [17], six patients received post-operative radiation therapy and seven received adjuvant chemotherapy (six received CHOP). Follow-up data was available for 10 patients of whom two recurred locally, five had distant metastasis and two died of disease. The authors concluded that large cell lymphoma chemotherapy was a reasonable approach in patients with HS. 2-Chlorodeoxyadenosine (2-CdA), a purine analog known to have both in vitro and in vivo activity against monocytes [18], has been successfully used in the treatment of disseminated Langerhans cell histiocytosis [19]. Buonocore et al. [10] described a 3-year-old male with chemoresistant HS who had clinical and radiological improvement with radiation therapy and 2-CdA, though long-term outcome was not provided. The authors also describe an adult HS patient who achieved clinical remission after five cycles of 2-CdA, with eventual disease recurrence. Anecdotal evidence exists for the use of thalidomide monotherapy, though durable remissions have not been described [20,21]. It is hoped that further study of cases such as that presented here will elucidate the pathogenesis of HS and aid in the development of novel, urgently needed therapeutic approaches.

REFERENCES

1. Swerdlow S, Campo E, Harris N, et al. editors. WHO classification of tumours of haematopoietic and lymphoid tissues. Lyon: International Agency for Research on Cancer; 2008.
2. Xie H, Ye M, Feng R, et al. Stepwise reprogramming of B cells into macrophages. Cell 2004;117:663–676.
3. Soslow RA, Davis RE, Warnke RA, et al. True histiocytic lymphoma following therapy for lymphoblastic neoplasms. Blood 1996;87:5207–5212.
4. van der Kwast TH, van Dongen JJ, Michiels JJ, et al. T-lymphoblastic lymphoma terminating as malignant histiocytosis with rearrangement of immunoglobulin heavy chain gene. Leukemia 1991;5:78–82.
5. Feldman AL, Minniti C, Santi M, et al. Histiocytic sarcoma after acute lymphoblastic leukaemia: A common clonal origin. Lancet Oncol 2004;5:248–250.
6. McClure R, Khoury J, Feldman A, et al. Clonal relationship between precursor B-cell acute lymphoblastic leukemia and histiocytic sarcoma: A case report and discussion in the context of similar cases. Leuk Res 2010;34:e71–e73.
7. Costa da Cunha Castro E, Blazquez C, Boyd J, et al. Clinicopathologic features of histiocytic lesions following ALL, with a review of the literature. Pediatr Dev Pathol 2009:1.
8. Pileri SA, Grogan TM, Harris NL, et al. Tumours of histiocytes and accessory dendritic cells: An immunohistochemical approach to classification from the International Lymphoma Study Group based on 61 cases. Histopathology 2002;41:1–29.
9. Vos JA, Abbondanzo SL, Barekman CL, et al. Histiocytic sarcoma: A study of five cases including the histiocyte marker CD163. Mod Pathol 2005;18:693–704.
10. Buonocore S, Valente AL, Nightingale D, et al. Histiocytic sarcoma in a 3-year-old male: A case report. Pediatrics 2005;116:e322–e325.
11. El-Matary W, Thorburn K, Baillie C, et al. Histiocytic sarcoma presenting with chylous ascites in a 7-month-old infant: A case report. J Pediatr Hematol Oncol 2009;31:65–68.
12. Carrasco DR, Fenton T, Sukhdeo K, et al. The PTEN and INK4A/ARF tumor suppressors maintain myelolymphoid homeostasis and cooperate to constrain histiocytic sarcoma development in humans. Cancer Cell 2006;9:379–390.
13. Sulong S, Moorman AV, Irving JA, et al. A comprehensive analysis of the CDKN2A gene in childhood acute lymphoblastic leukemia reveals genomic deletion, copy number neutral loss of heterozygosity, and association with specific cytogenetic subgroups. Blood 2009;113:100–107.
14. Bouabdallah R, Abena P, Chetaille B, et al. True histiocytic lymphoma following B-acute lymphoblastic leukaemia: Case report with evidence for a common clonal origin in both neoplasms. Br J Haematol 2001;113:1047–1050.

15. Feldman AL, Arber DA, Pittaluga S, et al. Clonally related follicular lymphomas and histiocytic/dendritic cell sarcomas: Evidence for transdifferentiation of the follicular lymphoma clone. Blood 2008;111:5433–5439.
16. Cobaleda C, Jochum W, Busslinger M. Conversion of mature B cells into T cells by dedifferentiation to uncommitted progenitors. Nature 2007;449:473–477.
17. Hornick JL, Jaffe ES, Fletcher CD. Extranodal histiocytic sarcoma: Clinicopathologic analysis of 14 cases of a rare epithelioid malignancy. Am J Surg Pathol 2004;28:1133–1144.
18. Carrera CJ, Terai C, Lotz M, et al. Potent toxicity of 2-chlorodeoxyadenosine toward human monocytes in vitro and in vivo. A novel approach to immunosuppressive therapy. J Clin Invest 1990;86:1480–1488.
19. Rodriguez-Galindo C, Kelly P, Jeng M, et al. Treatment of children with Langerhans cell histiocytosis with 2-chlorodeoxyadenosine. Am J Hematol 2002;69:179–184.
20. Abidi MH, Tove I, Ibrahim RB, et al. Thalidomide for the treatment of histiocytic sarcoma after hematopoietic stem cell transplant. Am J Hematol 2007;82:932–933.
21. Dalle JH, Leblond P, Decouvelaere A, et al. Efficacy of thalidomide in a child with histiocytic sarcoma following allogeneic bone marrow transplantation for T-ALL. Leukemia 2003;17:2056–2057.
