# Supplementary material for: An 8-year-old girl with secondary histiocytic sarcoma with BRAFV600 mutation following T-cell acute lymphoblastic leukemia demonstrating stable disease for 3 years on dabrafenib and trametinib – a case report and literature review
Source: BMC Pediatr. 2025 Mar 8;25:178. doi: 10.1186/s12887-025-05539-2 (PMC11889787; doi:10.1186/s12887-025-05539-2)
Supplement: Supplementary file 3 — Supplementary Material 3 [file 12887_2025_5539_MOESM3_ESM.pdf]

Journal of Health Sciences

CASE REPORT Open Access

Palliative care in the home: a case study of secondary histiocytic sarcoma in a 3-year-old child

Zuzana Karabová[1,3], Lubica Ilievová[2]

[1]Faculty of Health Sciences and Social Work, Department of Nursing, University of Trnava, Slovakia. [2]Faculty of Health Sciences and Social Work, Department of Nursing, University of Trnava, Slovakia. [3]Children's University Hospital and Clinic, Department of Pediatric Hematology and Oncology, Bratislava, Slovakia.

ABSTRACT

This article describes the medical, psychological, and social challenges encountered during home-based, family-centred palliative care of a 3-year-old female with secondary histiocytic sarcoma diagnosed during treatment for T-cell acute lymphoblastic leukaemia. Histiocytic sarcoma is an exceedingly rare cancer in adults, but even less frequent and highly aggressive when presenting as a secondary cancer in children. Comprehensive, multidisciplinary paediatric hospice care services are not widely available across Slovakia, thus limiting the number of patients and families offered such highly specialized end-of-life care. This case study illustrates the primary benefits for the child and family of such a program as well as the impact on the medical and nursing professionals working in the field of paediatric haematology-oncology.

Keywords: paediatric; palliative; secondary cancer; histiocytic sarcoma; home care; hospice.

INTRODUCTION

Cancer is relatively rare in childhood, with almost 1 in 500 children developing some form of cancer by 14 years of age (1). Acute lymphoblastic leukaemia (ALL) is the most common malignancy diagnosed in children (2). In Europe, ALL accounts for approximately 80% of leukaemias among children aged 0–14 years with a peak incidence in children aged 2-5 years (1,3). More than 80% of children with ALL are cured with current treatment regimens but are at risk of developing secondary cancers (3,4).

*Corresponding author: Ľubica Ilievová
Faculty of Health Sciences and Social Work,
Department of Nursing, University of Trnava,
Univerzitné námestie 1, Trnava, 918 43, Slovakia
E-mail: ilievova.lubica@truni.sk

Submitted 22 September 2013 / Accepted 10 December 2013

We present a case study that highlights the challenges to providing comprehensive, family-centred, home-based hospice care to a 3-year-old female battling the terminal stages of histiocytic sarcoma, an aggressive secondary malignancy. Histiocytic sarcoma is an exceedingly rare malignancy accounting for less than 1% of all haemato-lymphoid cancers and most commonly occurs in adults (5,6). Only a few reports of bona fide histiocytic sarcoma exist in the literature, mostly involving adults (7,8). A recent literature search uncovered only four published case reports of secondary histiocytic sarcoma in children (8-11).

Believes that the family home is the place that best meets the needs of terminally ill children and that symptom management must be an integral part of palliative care (12). A crucial element of com-

UNIVERSITY OF SARAJEVO
FACULTY OF HEALTH STUDIES

© 2013 Zuzana Karabová, Lubica Ilievová; licensee University of Sarajevo - Faculty of Health Studies. This is an Open Access article distributed under the terms of the Creative Commons Attribution License (http://creativecommons.org/licenses/by/2.0), which permits unrestricted use, distribution, and reproduction in any medium, provided the original work is properly cited.

prehensive, family-centred, paediatric hospice care is to provide consistent, seamless care, regardless of where the child is being treated (13). The child discussed in this case study was diagnosed with histiocytic sarcoma during maintenance treatment for T-cell ALL. Given the poor prognosis of childhood secondary histiocytic sarcoma, the paediatric patient described here received family-centred hospice care in the home.

CASE STUDY

A case study report was selected as the most suitable method to describe the home-based palliative nursing care of this terminally ill child, referred to here by the pseudonym 'Katarina'. Information was extracted from medical records and nursing notes covering the period when she received palliative care in the family home. During informal interviews, special attention was paid to elements of Katarina's verbal and non-verbal communication. How the mother adapted to the provision of palliative care was also noted. The mother provided written consent for the use of all information presented in this report.

Katarina's social and medical history

Katarina was the only child of healthy parents. Katarina enjoyed a stable emotional relationship with her mother and maternal grandparents with whom she lived in a common family home. Her father did not reside with Katarina and her mother. Katarina was born by caesarean section at 26 weeks of gestation weighing 690 grams and suffering from respiratory insufficiency and anaemia.

At two and a half years of age, Katarina developed low-grade fever, loss of appetite, abdominal pain and a white cell count of 458,900. Bone marrow biopsy confirmed the diagnosis of T-cell ALL. Katarina completed the induction and consolidation phases of the IC BFM 2002 protocol for ALL (14) and prophylactic cranial irradiation. Two months into ALL maintenance chemotherapy with oral mercaptopurine and intrathecal methotrexate, Katarina, now 3 years of age, developed abdominal pain, colitis, and abdominal lymphadenopathy. Lymph node biopsy revealed histiocytic sarcoma. Shortly thereafter, intestinal invagination resulted in laparotomy with lymphadenectomy and bowel resection. Histological examination confirmed the presence of a secondary malignancy consistent with histiocytic sarcoma.

Katarina's clinical course was characterized by rapid progression of her histiocytic sarcoma in the abdomen, pelvis, and lungs. Over the ensuing four months, Katarina received curative chemotherapy consisting of dexamethasone and cyclophosphamide followed by reduced-dose clofarabine, etoposide and cyclophosphamide. Her clinical condition worsened with profound myelosuppression and further progression of the abdominal tumour masses. Intensive chemotherapy was therefore stopped.

End of life care in the home

Katarina's parents agreed to end of life palliative care in the family home. A multidisciplinary team from an established 24-hour paediatric mobile hospice care service assumed responsibility of Katarina's complete symptom management. The home was equipped with oxygen and a supply of anti-emetics, laxatives, and sterile dressings. The nursing team incorporated Katarina's mother into the daily routine of her daughter's care while staying directly involved in the child's hygiene to ensure that she didn't develop pressure ulcers. They also monitored Katarina's pain using the Faces Pain Rating Scale—FPRS (15) alerting the team physician when modification to her analgesic therapy was required, thus resulting in superior pain control. Katarina was at high risk for difficult to manage constipation owing to her opiod dependence and reduced mobility caused by increasing ascites. Oral laxatives, rectal suppositories, and enemas were used prophylactically and therapeutically.

Katarina's first nights at home were characterized by restlessness during sleep until she achieved an undisturbed sleeping pattern. She repeatedly verified her mother's presence in the room throughout the night via eye contact. Despite the medical management of her shortness of breath and cough, she spent most of the day and night in the Fowler position to alleviate her laboured breathing. Hydration and nutrition were ensured with an oral regimen, easily digestible foods, and nutritional supplements.

Short hospitals stays were necessary on occasion for transfusions and albumin therapy to manage in-

creasing ascites. During this time, she repeatedly verbalized her immediate wish to return home. While in the hospital playroom, she drew predominantly black and grey-coloured pictures throwing aside red or yellow pencils when they were offered only to return to draw again with the black or grey pencils. Once back in the family home and in the care of the hospice team, the mother, nurses and a special educator kept Katarina constantly stimulated and motivated in playful activities. She found stability and consolation in being read the same fairy tales and watching the same videos repeatedly.

Parallel family care

The hospice nurses practiced effective communication with both Katarina and her mother. Their work was based on interaction. In general it is true that person's personality traits predetermine how to person establishes relationships with the surroungings and whether the person contributes, when communicating, to the atmosphere of trust or on the contrary sets it back. Also the nurse's approach and communication leaves a response in the patient's experiencing that may be even stronger due to the patient's vulnerability. If the patient experiences anxiety , uncertainty or fear owing to their state of health or a situation they are in, than the patient requires the atmosphere of safety, understanding and support (16). Katarina's mother received medical, psychological, and emotional support from hospice care doctors, nurses, psychologists and the case social worker. She was exhausted both mentally and physically having accompanied Katarina through her initial lengthy treatment for ALL, the diagnosis of her secondary histiocyctic sarcoma, its failed treatment culminating in home-based end of life palliative care. Her anxiety and fears were managed through psychological support and anti-anxiety medication while the child's grandparents provided additional emotional support.

The Final Stage

While still in the family home, Katarina gradually stopped communicating with the multidisciplinary team members, her grandparents, and then her mother. She exhibited signs of silent suffering, preferred to remain alone in her room and communicated only minimally with her favourite stuffed toy. Her clinical status deteriorated over several days with a decrease in level of consciousness, worsening dyspnoea, a cold grey paling of the skin, urinary retention, marked facial, and lower extremity oedema. Approximately 7 months following the diagnosis of histiocyctic sarcoma, Katarina died surrounded by her mother and grandparents, in a quiet home environment from progression of the underlying disease, respiratory failure, and cardiac arrest.

DISCUSSION

The World Health Organization defines palliative care for children as the active total care of the child's body, mind, and spirit, which also involves giving support to the family. Its purpose is to improve the quality of life of young patients and their families, and in the vast majority of cases the home is the best place to provide such care (17). Paediatric palliative hospice care relies on the value of the home as the place where one lives, rejoices, dies, and mourns. To be at home is a natural need of each child. Home is the place that best meets the needs of terminally ill and dying children (12). This case study demonstrates that the goals of paediatric palliative care can be achieved in the home environment by a multidisciplinary professional hospice health care team even in children with difficult to treat, rare, fatal malignancies. Reported that the death of a child due to cancer brings more distress to the child's parents than the death of a child due to another chronic illness (18). That life after the loss of a child is difficult, full of pain and sorrow and that such pain cannot be removed or avoided. It can be however, partially shared with others.

The benefits of an established paediatric hospice home care program also extend beyond the child and the child's family to the oncology nurses working in large specialized paediatric haematology-oncology institutions. End of life paediatric palliative hospice home care therefore provides a certain degree of emotional protection for nurses by relieving these hospital-based professionals from the repeated experience of sharing in the dying process.

Given the benefits of the paediatric home-based hospice care illustrated by this single case study, implementation of such services on a national scale

would thus greatly improve the provision of quality multidisciplinary paediatric oncology health care in Slovakia.

CONCLUSION

In this case report, we describe the case of a 3-year-old child who received family-centered palliative care in the home for the rare secondary malignancy of histiocytic sarcoma. Through case reporting we have highlighted the challenges encountered in providing home-based hospice care to a child in the terminal stages of life and identified its concrete benefits to the child, her family, hospital-based oncology nurses, and its potential impact on a nation-wide basis. This case report also serves to underscore the importance of cooperation between the child's parents, in this case the mother, and the paediatric hospice home care multidisciplinary team, as well as the participation of the university department paediatric haematology-oncology health care team.

COMPETING INTERESTS

The authors declare no conflict of interest.

REFERENCES

1. Cancer Research UK. CancerStats: Childhood Cancer – Great Britain & UK. November 2010.
2. Kanwar VS. Pediatric Acute Lymphoblastic Leukemia. MEDSCAPE Reference. Accessed May 31, 2012. http://emedicine.medscape.com
3. Pui CH, Robison LL, Look AT. Acute lymphoblastic leukaemia. Lancet. 2008, 371(9617):1030-43.
4. Hunger SP, Lu X, Devidas M, Camitta BM, Gaynon PS, Winick NJ, Reaman GH, Carroll WL. Improved Survival for Children and Adolescents With Acute Lymphoblastic Leukemia Between 1990 and 2005: A Report From the Children's Oncology Group. J Clin Oncol. 2012; 30(14):1663-9
5. Vos J, Abbondanzo SL, Barekman CL, Andriko JW, Miettinen M, Aguilera NS. Histiocytic sarcoma: a study of five cases including the histiocyte marker CD163. Modern Pathology. 2005; (18):693–704.
6. Chang KL. Histiocytic Sarcoma. Accessed May 2013. www.lasop.org/pgs/hdouts/2006-05_Case5.pdf
7. Pileri SA, Grogan TM, Harris NL, et al. Tumours of histiocytes and accessory dendritic cells: an immunohistochemical approach to classification from the International Lymphoma Study Group based on 61 cases. Histopathology. 2002;41(1):1–29.
8. Buonocore S, Valente AL, Nightingale D, Bogart J, Souid AK. Histiocytic Sarcoma in a 3-Year-Old Male: A Case Report. Pediatrics, published online July 15, 2005.
9. Jain M, Nangia A, Bajaj P. Malignant histiocytosis in childhood: A case report. Diagn Cytopathol. 1999;21(5):359-61.
10. Dalle JH, Leblond P, Decouvelaere A, Yakoub-Agha I, Preudhomme I, Nelken B, Mazingue F. Efficacy of thalidomide in a child with histiocytic sarcoma following allogeneic bone marrow tranplantation for T-ALL. Leukemia. 2003;(17):2056–2057.
11. Li YX, Wu HJ, Zhang JJ, Pan KL, Yu P, Fu X, Wang Z. A case report of childhood histocytic sarcoma. Zhongguo Dang Dai Er Ke Za Zhi. 2010;12(5):405-6. [Article in Chinese]
12. Jasenková, M. How to live together unitl the end. 1st edition. Bratislava: Cicero, 2005. p 13.
13. Shivani S. Tripathi, et al. Pediatric Palliative Care in the Medical Home. Pediatric Annals. 2012;41(3):112-116.
14. ALL IC-BFM 2002. A Randomized Trial of the IBFM-SG for the Management of Childhood non-B Acute Lymphoblastic Leukemia, 2002.
15. Wong DL, Hockenberry-Eaton M, Wilson D, Winkelstein ML, Schwartz P. Wong's Essentials of Pediatric Nursing, 6th ed, St. Louis, 2001, p. 1301.
16. Ilievová Ľ, Qualifications for excercising the profession of nurse. Assisting professions in the context of university education. Prague, 2010. p. 11-21.
17. Benini F, Spizzichino M, Trapanotto M, Ferrante A. Pediatric palliative care. Italian Journal of Pediatrics. 2008;(34):4.
18. James L, Johnson B. The Needs of Parents of Pediatric Oncology Patients During the Palliative Care Phase. Journal of Pediatric Oncology Nursing. 1997;(14)2:83.
