# Supplementary material for: An 8-year-old girl with secondary histiocytic sarcoma with BRAFV600 mutation following T-cell acute lymphoblastic leukemia demonstrating stable disease for 3 years on dabrafenib and trametinib – a case report and literature review
Source: BMC Pediatr. 2025 Mar 8;25:178. doi: 10.1186/s12887-025-05539-2 (PMC11889787; doi:10.1186/s12887-025-05539-2)
Supplement: Supplementary file 4 — Supplementary Material 4 [file 12887_2025_5539_MOESM4_ESM.pdf]

Pediatr Blood Cancer 2015;62:1656–1660

BRIEF REPORT

Secondary Histiocytic Sarcoma May Cause Apparent Persistence or Recurrence of Minimal Residual Disease in Childhood Acute Lymphoblastic Leukemia

Julia Alten, MD,[1] Wolfram Klapper, MD,[2] Ivo Leuschner, MD,[3] Cornelia Eckert, PhD,[4] Rita Beier, MD,[5] Elisabeth Vallo, MD,[6] Martin Krause, MD,[1] Alexander Claviez, MD,[1] Simon Vieth, MD,[1] Kirsten Bleckmann, MD,[1] Anja Möricke, MD,[1] Martin Schrappe, MD,[1] and Gunnar Cario, MD[1]*

Histiocytic sarcoma (HS) is a rare disease with poor prognosis which may develop subsequent to acute lymphoblastic leukemia (ALL). Here we report two children treated within the AIEOP-BFM ALL 2009 trial: one patient succumbed to fulminant hemophagocytic lymphohistiocytosis triggered by HS during ALL maintenance therapy, the other patient had a smoldering course of HS for over 2 years, and subsequently died after allogeneic stem cell transplantation. In both cases, HS and ALL were clonally related and apparent return of minimal residual disease (MRD) was detected by qPCR in bone marrow. Thus, HS should be considered in ALL when MRD appears to persist or reappear. Pediatr Blood Cancer 2015;62:1656–1660. © 2015 Wiley Periodicals, Inc.

Key words: acute lymphoblastic leukemia; histiocytic sarcoma; minimal residual disease; molecular MRD reappearance

INTRODUCTION

Histiocytic sarcoma (HS) is a rare, aggressive non-Langerhans histiocytic disorder that has been reported in association with hematological malignancies including acute lymphoblastic leukemia (ALL) and lymphoma [1–7]. Tumor cells are derived from the monocyte-macrophage lineage and diagnosis requires the presence of histiocytic markers and systematic exclusion of B- and T-cell markers [8]. HS may involve lymph nodes, bone marrow (BM), intestine, skin, and other organs (Table I), [1,8] and dismal prognosis is caused by rapid progression and poor response to therapy [2,9,10]. In some cases, histiocytic lesions share the same molecular-cytogenetic features with the original lymphoma or leukemia [3–6,11]. Minimal residual disease (MRD) detection via quantitative real-time PCR (qPCR) measurement of clone-specific immunoglobulin- and T-cell-receptor (Ig/TCR) gene rearrangements is commonly used for disease monitoring and risk stratification during ALL treatment [12,13]. MRD reappearance represents a conversion from MRD negativity to quantifiable MRD positivity [14] and can be equated with MRD relapse in specific ALL protocols [15].

We report two children with secondary HS subsequent and clonally related to T-cell ALL (T-ALL) as determined by identical TCR gene rearrangements. Both patients were treated within the AIEOP-BFM ALL 2009 trial and the first signs of HS including MRD reappearance in BM developed during treatment. However, disease kinetics varied considerably between the two patients.

CASE DESCRIPTION

Patient I

A 6-year old male patient was diagnosed with cortical T-ALL (CD7+, CD2+, CD1a+, CD3+, cyCD3+, TCR-αβ+, TDT−). He had CNS negative disease, had no cytogenetic high risk features, but had heterozygous deletion of CDKN2A. He was treated on the AIEOP-BFM ALL 2009 trial, T-ALL/non-HR arm (prednisone good response, MRD-level $1 \times 10E^{-4}$ on day 33, MRD negative at week 12 of treatment). Six months after the start of maintenance therapy (methotrexate and 6-mercaptopurine), the patient developed fever and thrombocytopenia and despite interruption of maintenance therapy pancytopenia progressed in an otherwise not severely compromised child. Four weeks after the onset of these symptoms, the patient rapidly deteriorated with hepatosplenomegaly, ascites, abdominal lymphadenopathy, progressive liver dysfunction, and respiratory failure. Relapse was excluded by BM morphology and hemophagocytic lymphohistiocytosis (HLH) was diagnosed [16]. Despite highly intensive immunosuppressive treatment with dexamethasone, etoposide, anti-thymocyte-globulin, and basiliximab, the patient died 10 days after diagnosis of HLH due to fulminant multiorgan failure. Histological evaluation of an abdominal lymph node (CD68+, CD163+, CD3−), removed during laparotomy following abdominal bleeding, revealed the diagnosis of HS (Supplemental Fig. 1). Additionally, heterozygous deletion of CDKN2A and detection of the known TCR gene rearrangements in the lymph node tissue confirmed a clonal

Additional Supporting Information may be found in the online version of this article.

Abbreviations: ALL, acute lymphoblastic leukemia; BM, bone marrow; CNS, central nervous system; HLH, hemophagocytic lymphohistiocytosis; HS, histiocytic sarcoma; Ig/TCR, immunoglobulin- and T-cell-receptor; MRD, minimal residual disease; PB, peripheral blood; pB-ALL, precursor B-cell ALL; qPCR, quantitative real-time PCR; SCT, allogeneic stem cell transplantation; T-ALL, T-cell ALL

[1]Department of Pediatrics, University Medical Center Schleswig-Holstein, Campus Kiel, Germany; [2]Department of Pathology, Lymph Node Registry Kiel, Institute of Hematopathology University Hospital Schleswig-Holstein, Campus Kiel, Germany; [3]Department of Pathology, Kiel Pediatric Tumor Registry, Institute of Pediatric Pathology, University Hospital Schleswig-Holstein, Campus Kiel, Germany; [4]Department of Pediatric Oncology/Hematology, Charité, University Medicine, Berlin, Germany; [5]Division of Pediatric Hematology and Oncology, Department of Pediatrics, Hannover Medical School, Hannover, Germany; [6]Department of Pediatrics, Klinikum Lippe Detmold, Detmold, Germany

Conflict of interest: Nothing to declare.

*Correspondence to: Gunnar Cario, Pediatric Oncology/Hematology, University Hospital Schleswig-Holstein, Campus Kiel, Arnold-Heller-Str.3, 24105 Kiel, Germany. E-mail: gunnar.cario@uksh.de

Received 15 January 2015; Accepted 24 February 2015

© 2015 Wiley Periodicals, Inc.
DOI 10.1002/pbc.25523
Published online 1 April 2015 in Wiley Online Library (wileyonlinelibrary.com).

TABLE I. Published Cases of Histiocytic Sarcoma Associated With Acute Lymphoblastic Leukemia

No.	Author	Age/sex	IP	SMN	Clonality	Onset	Organ involvement	Treatment	Outcome
1	patient I	6 y/m	T-ALL	HS	yes	15 mo after ID	BM, liver, spleen, LN	DXM, ETO, ATG, BAS	Death (after 6 weeks due to fulminant HLH)
2	patient II	10 y/m	T-ALL	HS	yes	12 mo after ID	BM, skin, liver, spleen	DXM, VCR, MTX, ASP, ARAC, IDA, CY, NEL, SCT	Death (3 weeks after SCT due to MOF)
3	Dictor et al., 2009 [2]	47 y/f	pB-ALL	HS	yes	14 y after ID	LN, BM	CY, DOXO, VCR, ETO, PDN	Death (after 2 months due to sepsis)
4	Feldman et al., 2004 [3]	14 y/m	preB-ALL	HS	yes	21 mo after ID	BM, spleen, kidney	VCR, CY, DNR, MTX, ETO, ARAC, PDN, SCT	N/K
5	Castro et al., 2010 [4]	5 y/m	T-ALL	HS	yes	6 mo after ID	BN	chemotherapy	Death (due to disease)
6	Castro et al., 2009 [4]	15 y/m	preB-ALL	HS	N/K	3 mo after ID	soft tissue, BN, lung	chemotherapy	Death (due to disease)
7	Castro et al., 2009 [4]	7 y/m	preB-ALL	HS	yes	6 mo after ID	BN, kidney	chemotherapy, SCT	Alive at last FUP
8	Castro et al., 2009 [4]	3 y/m	T-ALL	HS	N/K	16 mo after ID	liver, GIT	chemotherapy	Death (due to disease)
9	McClure et al., 2010 [5]	25 y/m	pB-ALL	HS	yes	4 mo after ID	spleen, skin	CY, VCR, DOXO, DXM, MTX, ARAC, SCT	Alive (last FUP 2 months after SCT abating skin lesions)
10	Kumar et al., 2011 [6]	4 y / m	preB-ALL	HS	yes	in maintenance (1 mo)	BN, BM, spleen	DXM, CY, MTX, IFO, ARAC, ETO, RTX	Death (after 1 year)
11	Pileri et al., 2002 [7]	26 y/m	N/K	HS	N/K	N/K	BN	RTX	ALL-relapse after 3 years
12	Chalasani et al., 2013 [9]	44 y/m	T-ALL	HS	no	16 y after ID	CNS	DXM, MTX, TMZ, RTX	Death (after 27 weeks due to disease)
13	Soslow et al., 1996 [10]	8 y/m	preB-ALL	THL	N/K	10 mo after ID	paraspinous, BN, lung, liver, spleen	ETO, MEP	Death (after 3 months)
14	Soslow et al., 1996 [10]	6 y/m	pB-ALL	THL	N/K	20 mo after ID	BN, paravertebral, lung, liver	IFO, ETO, CBP	Alive (last FUP 16 months after IDX, but progress of disease)
15	Bouabdallah et al., 2001 [11]	23 y/m	pB-ALL	THL	yes	4 y after ID	LN, GIT	DNR, CY, DTIC	Alive (last FUP 8 months after IDX)
16	Dalle et al., 2003 [18]	4 y/m	T-ALL	HS	possible	9 mo after SCT	BN, GIT, lung	Post-SCT: VIN, PDN, THA, DLI	Alive (last FUP 33 months after IDX, cont. treatment with THA)
17	Ganapule et al., 2014 [19]	4 y/m	T-ALL	HS	N/K	in maintenance (18 mo)	BN, lung	Palliative care	N/K
18	Wongchanchailert and Laosombat 2003 [20]	8 y/f	pB-ALL	THL	N/K	6 mo after ID	BN, extradural	CY, DNR, VCR, PDN	Death (ALL-relapse, death due to severe sepsis shortly after)

ARAC, cytosine arabinoside; ASP, E.coli asparaginase, ATG, anti-thymocyte globulin; BAS, basiliximab; BM, bone marrow; BN, bone; CBP, carboplatin; CNS, central nervous system; CY, cyclophosphamide; DLI, donor lymphocyte infusion; DNR, daunorubicin; DOXO, doxorubicin; DTIC, dacarbazine; f, female; IDX, diagnosis; DXM, dexamethasone; ETO, etoposide; FUP, follow-up; GIT, gastrointestinal tract; HLH, hemophagocytic lymphohistiocytosis; HS, histiocytic lymphoma; ID, initial diagnosis; IDA, idarubicin; IFO, ifosfamide; IP, immunophenotype; LN, lymph node; m, male; MEP,methylprednisolone; MO, months; MOF, multiorgan failure; MTX, methotrexate; NEL, nelarabine; N/K, not known; pB-ALL, precursor B-cell ALL; PDN, prednisone; RTX, radiotherapy; SCT, allogeneic stem cell transplantation; SMN, second malignant neoplasm; T-ALL, T-cell ALL; THA, thalidomide; THL, true histiocytic lymphoma; TMZ, temozolomide; VCR, vincristine; VIN, vinblastine; y, years.

relationship between HS and preceding T-ALL (Supplemental Table I). Retrospective quantification of MRD in peripheral blood (PB) and BM samples collected during maintenance therapy showed apparent MRD reappearance rising up to a level of $1 \times 10E^{-2}$ shortly after the onset of first symptoms of HS (Fig. 1A).

Patient II

A 10-year old male patient was diagnosed with cortical T-ALL (CD7+, CD2+, CD1a+, cyCD3+, TDT+, CD3−). He had CNS negative disease, with no cytogenetic high-risk features. He was treated on the AIEOP-BFM ALL 2009 trial, T-ALL/HR arm (prednisone poor response, MRD-level $1 \times 10E^{-3}$ on day 33, MRD negative at week 12 of treatment). The start of maintenance therapy was delayed (>15 months from diagnosis) due to pancytopenia and infectious complications, and treatment was terminated only 4 months later due to persistent thrombocytopenia. Thirty months after T-ALL diagnosis, the patient developed several granulomatous swellings (preauricular, thoracic, palatal) in addition to persistent thrombocytopenia (<100,000/μl). Histological evaluation and analysis of TCR gene rearrangements in biopsy tissue (CD68+, CD163+, CD3−) confirmed the

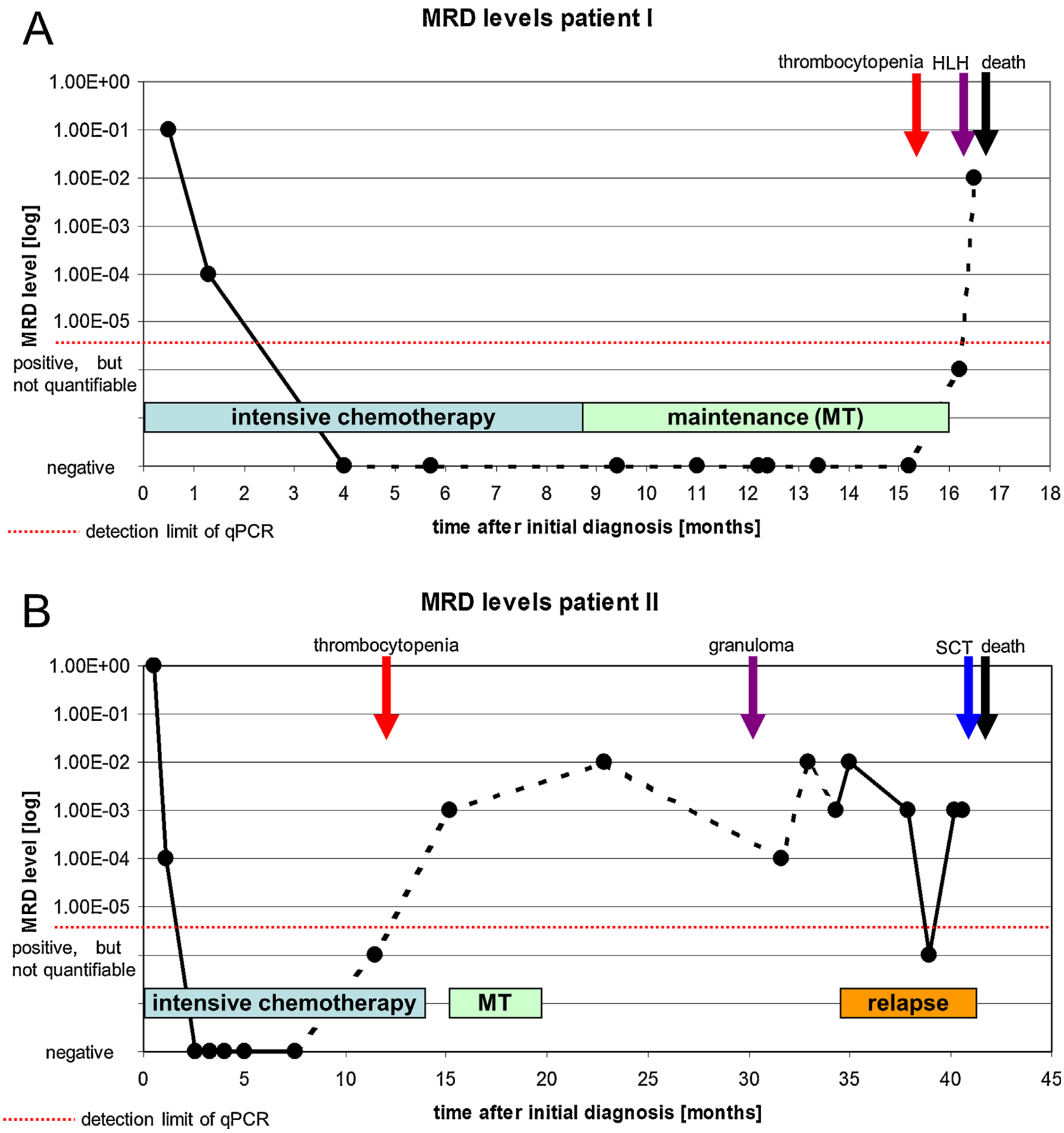

Fig. 1. MRD levels during the course of disease of (**A**) patient I and (**B**) patient II. After initial target screening for immunoglobulin- and T-cell receptor, gene rearrangements at the time of diagnosis MRD measurements via qPCR were performed on BM samples collected at protocol-defined time points during intensive chemotherapy (AIEOP-BFM ALL 2009 and ALL-Rez BFM registry) and were used for final risk stratification (black graph). Furthermore, PB as well as BM samples were collected during maintenance therapy and beyond from both patients in the context of an AIEOP-BFM ALL 2009 add-on study. Informed consent to the participation in this add-on study was obtained by the parents or legal guardian. MRD levels were measured retrospectively in these samples (dotted graph).

diagnosis of HS (Supplemental Fig. 1) clonally related to the preceding T-ALL (Supplemental Table I). Additionally, ALL relapse was excluded by BM morphology, immunophenotyping, and cytogenetics. Nevertheless, the patient received ALL relapse treatment (ALL-REZ BFM) including allogeneic stem cell transplantation (SCT) due to the lack of standardized treatment of HS and insufficient MRD clearance. He died 10 months after diagnosis of HS due to multiorgan failure shortly after SCT. Retrospective quantification of MRD in PB and BM samples collected during treatment and beyond (before the start of relapse treatment) showed apparent MRD reappearance during the last phase of intensive chemotherapy with smoldering MRD levels up to $1 \times 10E^{-2}$ as well as apparent MRD persistence during relapse treatment (Fig. 1B).

DISCUSSION

HS subsequent to ALL is a very rare event; including our two patients, 13 out of 18 published cases have been reported in children (Table I). However, limitations apply since not all histiocytic lesions are clearly categorized and diagnostic labels may vary [4,8]. Therefore, only cases of HS or true histiocytic lymphoma have been included. Summarizing the published cases, there is a male predominance (89%) with a median age at time of onset of 8 years and a slight shift toward pB-ALL (60%). In 80% of the cases, time of onset is within 2 years of initial diagnosis (during maintenance therapy). Site of onset, treatment modality (lack of standardized treatment), and outcome differ widely among patients, but usually the outcome has been very poor due to rapid progression and chemoresistance. In 10 out of 18 patients (55%), a clonal relationship between initial ALL and HS was described with respect to identical Ig/TCR gene rearrangements and/or cytogenetic features i.e., deletion of CDKN2A [6], (Table I).

The exact pathogenesis of shared clonality between HS and ALL still remains unclear. It has been postulated that both malignancies derive from a common abnormality in a precursor or progenitor cell trans-differentiating into another lineage [3,5,11]. The exact mechanism of reprogramming B- or T-cells into histiocytes/macrophages is still unknown, but considering onset during maintenance therapy in most cases (Table I), it is conceivable that either a selective pressure on histiocytic or progenitor cells, or a more favorable environment in maintenance therapy compared to intensive ALL-therapy might promote trans-differentiation. On the contrary, it has been suggested that two neoplastic populations sharing the same clonal origin might be already present at initial diagnosis. While malignant cells with histiocytic differentiation remain chemoresistant, ALL blasts respond to chemotherapy [3,17]. Following this hypothesis, clonally related HS detected by qPCR might be misinterpreted as MRD persistence or reappearance of initial ALL.

Both of our patients were MRD negative after 12 weeks of ALL-treatment and beyond, but showed apparent MRD reappearance (measured retrospectively) in BM and PB during maintenance therapy. At about the same time, thrombocytopenia as a first symptom of HS occurred. However, disease progression and MRD kinetics were completely different. While *patient I* presented a fulminant course with all symptoms of HLH and rapidly increasing MRD levels shortly after onset of thrombocytopenia, *patient II* showed a smoldering MRD persistence in BM for almost 2 years in addition to a late extramedullary manifestation of HS prior to disease progression.

In conclusion, HS subsequent to ALL is a rare event, which may occur during maintenance therapy or even after termination of ALL-treatment. Since time of onset, clinical features as well as disease progression and treatment response vary considerably among patients, presence of HS should be considered in ALL patients with unclear pancytopenia, lymph node swelling, or even HLH. Additionally, patients with apparent MRD refractory ALL late in treatment (i.e., MRD persistence at $\geq 1 \times 10E^{-2/-3}$ as measured by qPCR) might have two coexisting disorders ("chemo-resistant" HS and "chemosensitive" ALL) sharing the same clonal origin and, therefore, leading to a continuous MRD positivity in BM. Furthermore, if reappearance of MRD is detected by qPCR, HS should be considered as a differential diagnosis to ALL relapse and patients should be carefully evaluated before ALL relapse treatment is initiated.

ACKNOWLEDGMENT

We would like to thank Dr. Rolf Köhler of the Institute for Human Genetics, Heidelberg University for the identification of Ig/TCR gene rearrangements at ALL diagnosis.

REFERENCES

1. Mathe G,Gerard-Marchant R, Texier JL, Schlumberger JR, Berumen L, Paintrand M. The two varieties of lymphoid tissue "reticulosarcomas," histiocytic and histioblastic types. Br J Cancer 1970;24:687–695.
2. Dictor M, Warenholt J, Gyorgy C, Mansson I, Larsson G. Clonal evolution to histiocytic sarcoma with the BCR/ABL rearrangement 14 years after acute lymphoblastic leukemia. Leuk Lymphoma 2009;50: 1892–1895.
3. Feldman AL, Minniti C, Santi M, Downing JR, Raffeld M, Jaffe ES. Histiocytic sarcoma after acute lymphoblastic leukaemia: A common clonal origin. Lancet Oncol 2004;5:248–250.
4. Castro EC, Blazquez C, Boyd J, Correa H, de Chadarevian JP, Felgar RE, Graf N, Levy N, Lowe EJ, Manning JT Jr., Proytcheva MA, Senger C, Shayan K, Sterba J, Werner A, Surti U, Jaffe R. Clinicopathologic features of histiocytic lesions following ALL, with a review of the literature. Pediatr Dev Pathol 2010;13:225–237.
5. McClure R, Khoury J, Feldman A, Ketterling R. Clonal relationship between precursor B-cell acute lymphoblastic leukemia and histiocytic sarcoma: A case report and discussion in the context of similar cases. Leuk Res 2010;34:e71–e73.
6. Kumar R, Khan SP, Joshi DD, Shaw GR, Ketterling RP, Feldman AL. Pediatric histiocytic sarcoma clonally related to precursor B-cell acute lymphoblastic leukemia with homozygous deletion of CDKN2A encoding p16INK4A. Pediatr Blood Cancer 2011;56:307–310.
7. Pileri SA, Grogan TM, Harris NL, Banks P, Campo E, Chan JK, Favera RD, Delsol G, De Wolf-Peeters C, Falini B, Gascoyne RD, Gaulard P, Gatter KC, Isaacson PG, Jaffe ES, Kluin P, Knowles DM, Mason DY, Mori S, Muller-Hermelink HK, Piris MA, Ralfkiaer E, Stein H, Su IJ, Warnke RA, Weiss LM. Tumours of histiocytes and accessory dendritic cells: An immunohistochemical approach to classification from the International Lymphoma Study Group based on 61 cases. Histopathology 2002;41:1–29.
8. Takahashi E, Nakamura S. Histiocytic sarcoma: An updated literature review based on the 2008 WHO classification. J Clin Exp Hematop 2013;53:1–8.
9. Chalasani S, Hennick MR, Hocking WG, Shaw GR, Lawler B. Unusual presentation of a rare cancer: Histiocytic sarcoma in the brain 16 years after treatment for acute lymphoblastic leukemia. Clin Med Res 2013;11:31–35.
10. Soslow RA, Davis RE, Warnke RA, Cleary ML, Kamel OW. True histiocytic lymphoma following therapy for lymphoblastic neoplasms. Blood 1996;87:5207–5212.
11. Bouabdallah R, Abena P, Chetaille B, Aurran-Schleinitz T, Sainty D, Dubus P, Arnoulet C, Coso D, Xerri L, Gastaut JA. True histiocytic lymphoma following B-acute lymphoblastic leukaemia: Case report with evidence for a common clonal origin in both neoplasms. Br J Haematol 2001;113: 1047–1050.
12. Flohr T, Schrauder A, Cazzaniga G, Panzer-Grumayer R, van der Velden V, Fischer S, Stanulla M, Basso G, Niggli FK, Schafer BW, Sutton R, Koehler R, Zimmermann M, Valsecchi MG, Gadner H, Masera G, Schrappe M, van Dongen JJ, Biondi A, Bartram CR. Minimal residual disease-directed risk stratification using real-time quantitative PCR analysis of immunoglobulin and T-cell receptor gene rearrangements in the international multicenter trial AIEOP-BFM ALL 2000 for childhood acute lymphoblastic leukemia. Leukemia 2008;22:771–782.
13. van der Velden VH, Cazzaniga G, Schrauder A, Hancock J, Bader P, Panzer-Grumayer ER, Flohr T, Sutton R, Cave H, Madsen HO, Cayuela JM, Trka J, Eckert C, Foroni L, Zur Stadt U, Beldjord K, Raff T, van der Schoot CE, van Dongen JJ. Analysis of minimal residual disease by Ig/TCR gene rearrangements: Guidelines for interpretation of real-time quantitative PCR data. Leukemia 2007;21:604–611.
14. Bruggemann M, Schrauder A, Raff T, Pfeifer H, Dworzak M, Ottmann OG, Asnafi V, Baruchel A, Bassan R, Benoit Y, Biondi A, Cave H, Dombret H, Fielding AK, Foa R, Gokbuget N, Goldstone AH, Goulden N, Henze G, Hoelzer D, Janka-Schaub GE, Macintyre EA, Pieters R, Rambaldi A, Ribera JM, Schmiegelow K, Spinelli O, Stary J, von Stackelberg A, Kneba M, Schrappe M, van Dongen JJ. Standardized MRD quantification in European ALL trials: Proceedings of the Second International Symposium on MRD assessment in Kiel, Germany, 18–20 September 2008. Leukemia 2010;24:521–535.
15. Raff T, Gokbuget N, Luschen S, Reutzel R, Ritgen M, Irmer S, Bottcher S, Horst HA, Kneba M, Hoelzer D, Bruggemann M. Molecular relapse in adult standard-risk ALL patients detected by prospective MRD

monitoring during and after maintenance treatment: Data from the GMALL 06/99 and 07/03 trials. Blood 2007;109:910–915.
16. Henter JI, Horne A, Arico M, Egeler RM, Filipovich AH, Imashuku S, Ladisch S, McClain K, Webb D, Winiarski J, Janka G. HLH- 2004 Diagnostic and therapeutic guidelines for hemophagocytic lymphohistiocytosis. Pediatr Blood Cancer 2007;48:124–131.
17. van der Kwast TH, van Dongen JJ, Michiels JJ, Hooijkaas H, Kappers MC, Hagemeijer A. T-lymphoblastic lymphoma terminating as malignant histiocytosis with rearrangement of immunoglobulin heavy chain gene. Leukemia 1991;5:78–82.
18. Dalle JH, Leblond P, Decouvelaere A, Yakoub-Agha I, Preudhomme C, Nelken B, Mazingue F. Efficacy of thalidomide in a child with histiocytic sarcoma following allogeneic bone marrow transplantation for T-ALL. Leukemia 2003;17:2056–2057.
19. Ganapule AP, Gupta M, Kokil G, Viswabandya A. Histiocytic sarcoma with acute lymphoblastic leukemia a rare association: Case report and literature review. Indian J Hematol Blood Transfus 2014;30(Suppl 1):305–308.
20. Wongchanchailert M, Laosombat V. True histiocytic lymphoma following acute lymphoblastic leukemia. Med Pediatr Oncol 2003;40:51–53.
