# Supplementary material for: An 8-year-old girl with secondary histiocytic sarcoma with BRAFV600 mutation following T-cell acute lymphoblastic leukemia demonstrating stable disease for 3 years on dabrafenib and trametinib – a case report and literature review
Source: BMC Pediatr. 2025 Mar 8;25:178. doi: 10.1186/s12887-025-05539-2 (PMC11889787; doi:10.1186/s12887-025-05539-2)
Supplement: Supplementary file 5 — Supplementary Material 5 [file 12887_2025_5539_MOESM5_ESM.pdf]

Indian J Hematol Blood Transfus
DOI 10.1007/s12288-014-0375-3

CASE REPORT

Histiocytic Sarcoma with Acute Lymphoblastic Leukemia a Rare Association: Case Report and Literature Review

Abhijeet P. Ganapule · Mayank Gupta · Gautami Kokil · Auro Viswabandya

Received: 3 March 2014 / Accepted: 21 March 2014
© Indian Society of Haematology & Transfusion Medicine 2014

Abstract Definition and diagnostic criteria for histiocytic sarcoma (HS) have changed over last two decades due to available new immunohistochemical markers, as well as better understanding of the biology of disease. We report here a case of 4 years old boy diagnosed as acute lymphoblastic leukemia (ALL), who later developed HS of pleura, when he was on maintenance phase of ALL protocol. HS constitutes less than 1 % of the haematolymphoid neoplasm, even more rare is association of HS with ALL. Thus reporting here a rare association of HS with ALL, will help in knowing the actual incidence of HS as well as association with ALL.

Keywords Histiocytic sarcoma (HS) · Haematolymphoid Neoplasm · Acute lymphoblastic leukemia (ALL)

Introduction

Histiocytic sarcoma (HS) is a neoplasm derived from mononuclear phagocytes or histiocytes, which have major role in processing and presenting antigens to T or B lymphocytes [1]. HS is a rare hematopoietic neoplasm constituting less than 1 % of the haematolymphoid neoplasm [2]. There are very few case reports of HS associated with acute lymphoblastic leukemia (ALL) [3–10].

Case Report

A 4 year old boy was evaluated for low grade fever, with no associated localising symptoms. On clinical examination he was pale with just palpable spleen, rest of the clinical examination was unremarkable. He was diagnosed to have T ALL based on peripheral smear showing 95 % blasts, bone marrow consistent with acute leukemia. Immunophenotyping done on bone marrow showed 97 % gated population with T ALL phenotype (CD7-97 %, CD5-82 %, CD2-84 %, CD3-85 %, CD34-67 %, HLA DR-93 %, CD1a-5 %). Bone marrow karyotype analysis was normal. He was started on Berlin Frankfurt Munich (BFM) ALL-1995 protocol, however was later shifted to relapse BFM-ALL 1995 protocol as there was poor steroid response on day +8. He overall tolerated the chemotherapy well, after 1.5 years of maintenance phase of relapse BFM ALL 1995 protocol he presented with dry, non-productive cough associated with low grade fever. Clinical examination revealed pallor, multiple tender nodules on the ribs measuring upto 1.5 cm and decreased air entry on the right side. Rest of the clinical examination was unremarkable. Computerised tomography (CT) of thorax showed right pleural thickening with compressive right lung atelectasis and multiple lytic lesion over vertebral end of the right 2nd, 7th, 8th and 9th ribs. Pleural biopsy showed fragments of fibrocollagenous tissue, few histiocytic aggregates and lymphocytes but no definite granuloma. Special stains and cultures for acid fast bacilli, other bacterial and fungal organisms were negative. A definitive diagnosis, whether inflammatory or neoplastic, could not be established as

A. P. Ganapule · A. Viswabandya (✉)
Department of Haematology, Christian Medical College and Hospital, Ida Scudder Road, Vellore 632004, Tamil Nadu, India
e-mail: aurov@cmcvellore.ac.in

M. Gupta · G. Kokil
Department of Pathology, Christian Medical College and Hospital, Ida Scudder Road, Vellore 632004, Tamil Nadu, India

only a few histiocytic aggregates were present. So subsequently he underwent partial decortication of right pleura with scrapping of the rib lesions, which were sent for histopathological examination. Both the pleural decortication specimen and scrapping showed sheets of polygonal cells displaying eccentrically placed vesicular nuclei, many of those with indentation, distinct to prominent nucleoli and moderate to abundant eosinophilic cytoplasm (Fig. 1a–c). Multinucleate giant cells (Fig. 1b) and occasional mitotic figures including atypical forms were seen. Foci of necrosis, lymphovascular emboli and infiltration of adjacent lung parenchyma (Fig. 2a, b) were evident. These polygonal cells were positive for CD163 (Fig. 3a), PGM-1 (Fig. 3b) and were negative for CD1a, calretinin, CD30, CD21, CD23, myeloperioxidase, pancytokeratin, EMA, HMB-45 and melan A, confirming the diagnosis of HS. Blood and bone marrow examination were normal. The diagnosis, treatment options and prognosis was discussed in detail with the family and they opted for palliative care at local place considering the dismal prognosis.

Discussion

The term HS was introduced by Mathe et al. in 1970, this was based on histologic similarities of the cells to macrophages [11]. It is also known as true histocytic lymphoma, malignant histiocytosis, histocytic medullary reticulosis, reticulum sarcoma and regressing atypical histocytosis [1, 11, 12].

HS is defined as malignant proliferation of cells showing morphologic and immunophenotypic features of mature histiocytes [12]. There is wide age variation from infancy to adults, with male predilection [1]. It commonly involves lymph node, however can occur at extranodal sites like gastrointestinal tract, spleen, soft tissue, skin, head and neck region, salivary gland, lung, mediastinum, breast, liver, pancreas, kidney, uterus, central nervous system, skeletal system and bone marrow [1, 12]. In our case, HS involved ribs and right sided pleura with infiltration of adjacent lung parenchyma, following treatment of T-ALL.

Morphologically, HS comprises of diffuse, non-cohesive proliferation of large cells [1, 12]. The nuclei show atypia

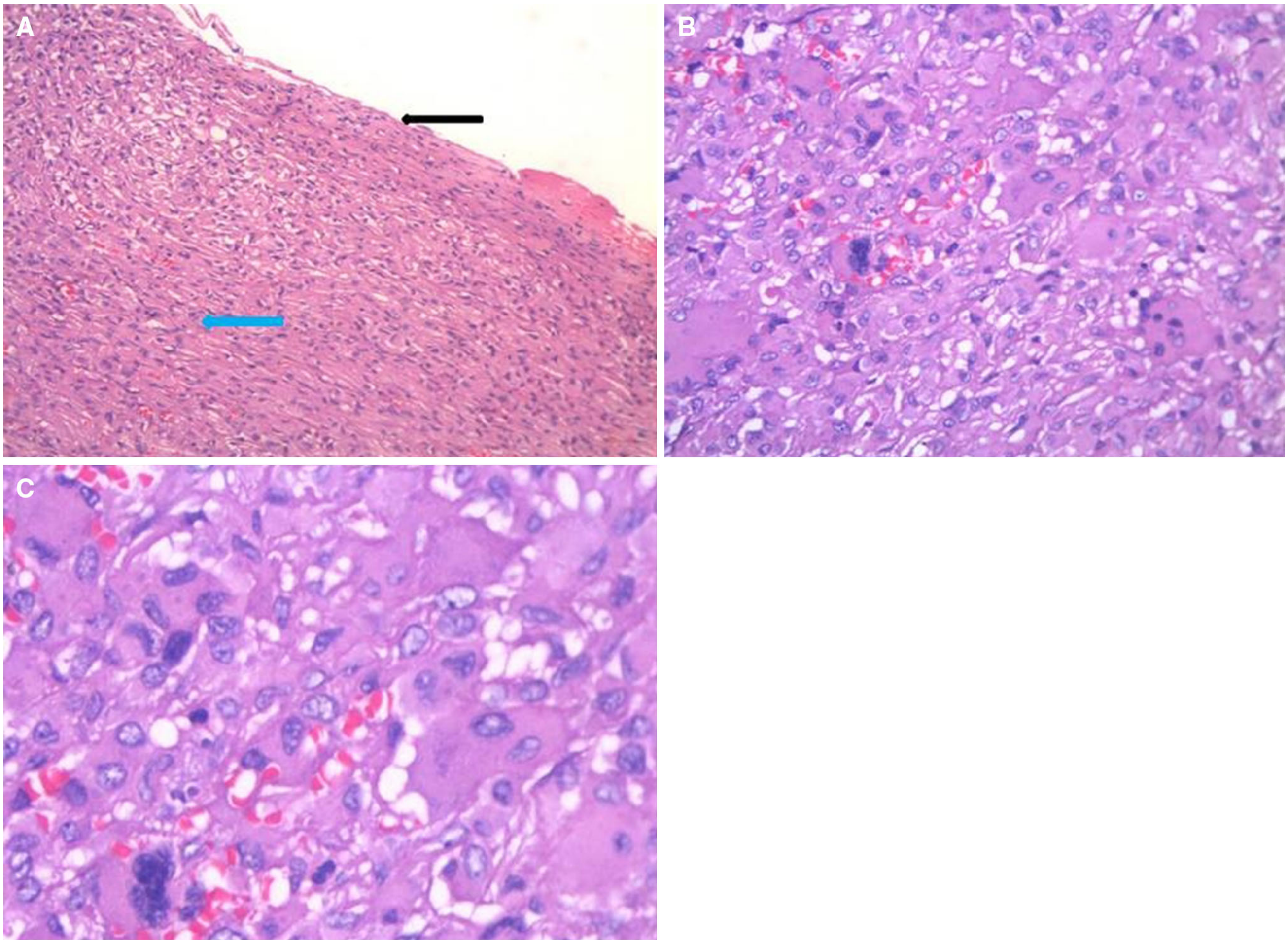

Fig. 1 **a** Sheets of neoplastic histiocytes (*light blue arrow*) with pleural tissue (*black arrow*), ×10 magnification, H&E. **b** Sheets of neoplastic histiocytes, ×20 magnification, H&E. **c** Same as in **b**, ×40 magnification. (Color figure online)

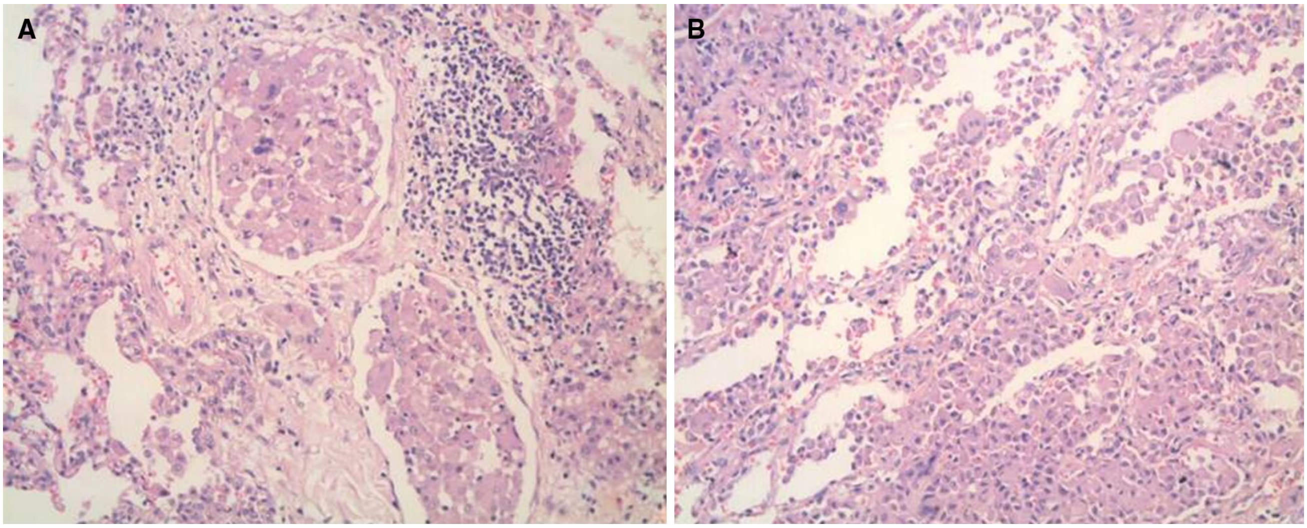

Fig. 2 **a** Lymphovascular tumor emboli (in *centre*) and lung parenchyma (on either side of tumor emboli), ×20 magnification, H&E. **b** Tumor within alveoli of lung, ×20 magnification, H&E

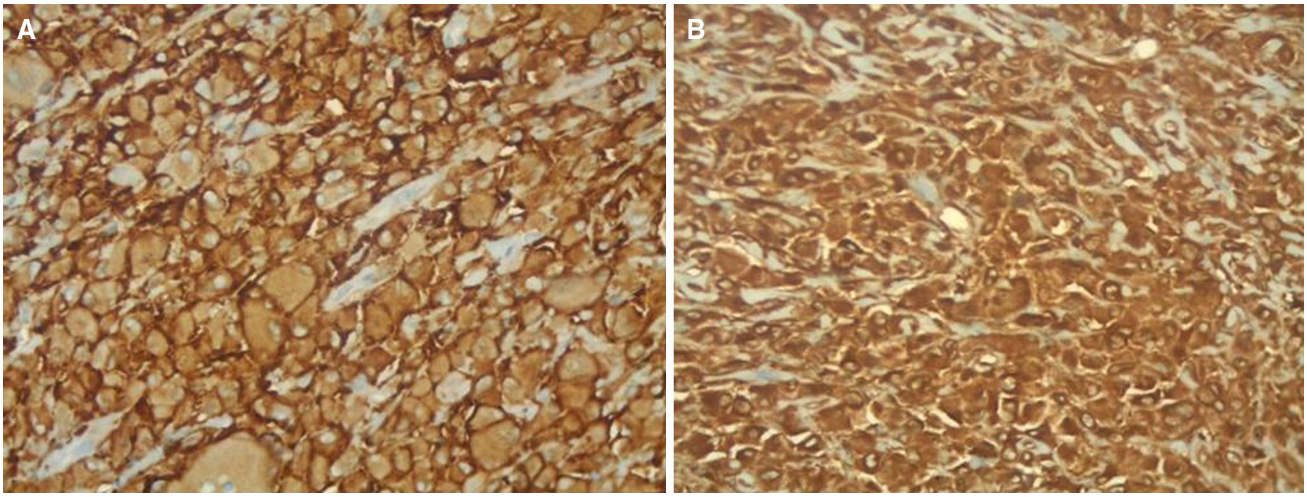

Fig. 3 **a** Tumor cells are diffusely and strongly positive for CD163, ×40 magnification, immunohistochemistry. **b** Tumor cells are diffusely and strongly positive for PGM-1, ×40 magnification, immunohistochemistry

and are generally large, round to oval, irregularly folded and eccentrically placed [1, 12]. Our case had similar histological findings. According to World Health Organisation (WHO) 2008 to make a diagnosis of HS one or more histiocytic markers should be positive for CD163, CD68 or lysozyme, with typical absence of Langerhans cell, follicular dendritic cell, myeloid, B cell and T cell, epithelial, melanin markers (CD1a, langerin, CD21, CD35, CD33, CD13, myeloperoxidase, pancytokeratin, EMA, HMB-45, melanin) [1, 12]. CD163, a scavenger receptor for haemoglobin–haptoglobin complex, is a new and more specific immunohistochemical (IHC) marker of monocytes/histiocytes than CD68 [1]. IHC findings in our case satisfied WHO 2008 criteria for diagnosing HS. According to WHO 2001 definition of HS, if required absence of clonal IgH or TCR gene rearrangement [13]. However, later it was found that some HS developing after or concurrent with follicular lymphoma shared identical genotype [14]. Based on this, WHO 2008 classification no longer strictly requires the absence of clonal IgH or TCR gene rearrangement for the diagnosis of HS [12].

HS can occur de novo, however there have been reports of histocytic lesions preceding or following ALL [3–10]. Castro E.C.C et al. described 15 histocytic lesion following ALL, of which 4 were HS [3]. The interval between diagnosis of ALL and development of histocytic lesion ranged from 3 months to 10 years [3]. In our case the histiocytic neoplasm involving pleura and ribs occurred

about 1.5 years after maintenance phase treatment for T-ALL. Similarly Dictor et al. reported a case of Philadelphia positive ALL 14 years later presented with histiocytic lesion [4]. Feldman et al., Van der kwast et al. and Kumar et al. reported a case each of HS after the treatment of ALL with a common clonal origin [5–7]. Chalasani reported a case of HS of brain 16 years post ALL which was refractory to all treatment modalities [8]. Willems et al. reported a case of HS in patient with B-ALL presenting with intestinal obstruction [9]. Saslow reported a cases series of 4 histocytic lymphoma post treatment of ALL (1 T-ALL and 2 B-ALL) [10]. The hypothesis proposed for histiocytic lesion developing in a patient post ALL treatment are: (1) Transdifferentiation of neoplastic B/T cells to malignant histocytes [1]. (2) Dedifferentiation of neoplastic B/T cells to early progenitors and subsequently redifferentiational on the histocytic lineage [1]. (3) Presence of common progenitor with differentiation along both B cell and histocytic/dendritic lineages [1]. HS has limited response to chemotherapy with high mortality, most of the patient die of progressive disease within 2 years. As this entity is rare there is no standard approach to treatment [1, 3].

Conclusion

HS is a rare haematolymphoid tumor. In the past these might have been reported as lymphoma, due to lack of ancillary techniques. It is important to be aware of histiocytic sarcoma and its diagnostic features, particularly in patients who have been on treatment for leukemia/lymphoma or mediastinal germ cell tumors. The time interval between development of HS (second malignancy) following treatment of leukemia/lymphoma (primary malignancy) is variable. There is no standard approach to treatment of HS and it has dismal prognosis. Thus reporting these cases might help to decipher the actual incidence of this lesion as well as help us understand this entity in more depth.

References

1. Takahashi E, Nakamura S et al (2013) Histocytic sarcoma: an updated literature review based on the 2008 WHO Classification. J Clin Exp Hematop 53:1–7
2. Jaffe ES (1985) Histiocytosis of lymph node, biology and differential diagnosis. Semin Diagn Pathol 5:376–390
3. Castro ECC, Blazquez C, Boyd et al (2010) Clinicopathologic features of histocytic lesion following ALL, with a review of the literature. Pediatr Dev Pathol 13:225–237
4. Dictor M, Warenholt J, Gyorgy C, Mansson I, Larsson G et al (2009) Clonal evolution to histiocytic sarcoma with BCR/ABL rearrangement 14 years after acute lymphoblastic leukemia. Leuk Lymphoma 50:1892–1895
5. Feldman AL, Minniti C, Santi M, Downing JR, Raffeld M, Jaffe E (2004) Histocytic sarcoma after acute lymphoblastic leukemia: a common clonal origin. Lancet 5:248–249
6. Kumar R, Khan SP, Joshi DD, Shaw GR, Ketterling RP, Feldman AL (2011) Pediatric histiocytic sarcoma clonally related to precursor B cell acute lymphoblastic leukemia with homozygous deletion of CDKN2A encoding p15INK4A. Pediatr Blood Cancer 56:307–310
7. Van der Kwast TH, Van Dongen JJ, Michiels JJ, Hoolikaas H, Kappers MC, Hagemejjer A (1991) T lymphoblastic lymphoma terminating as malignant histiocytosis with rearrangement of immunoglobin heavy chain gene. Leukemia 5:78–82
8. Chalasani S, Hennik MR et al (2013) Unusual presentation of rare cancer: histocytic sarcoma in brain 16 years after treatment of ALL. J Clin Med Res 11:31–35
9. Willem E, Van laer C, Billet J et al (2010) Histocytic sarcoma after precursor B lymphoblastic lymphoma a case report. Belgian J Haematol 1:28–31
10. Soslow RA, Davis RE, Warmke RA, Cleary ML, Kamel OW et al (1996) True hitocytic lymphoma following therapy for lymphoblastic neoplasm. Blood 87:5207–5212
11. Mathe G, Gerard MR, Texier JL et al (1970) The two vareties of lymphoid tissue 'retiulosarcoma', histocytic and histoblastic types. Br J Cancer 24:687–695
12. Gorgan TM, Pileri SA, Chan JKC, Weiss LM, Fletcher CDM (2008) Histocytic sarcoma. In: Swerdlow SH, Campo E, Harris NL, Jaffe ES, Pileri SA et al (eds) World Health Organisation classification of tumors, WHO classification of tumors of haematopoietic and lymphoid tissues, 4th edn. International Agency for Research on Cancer (IARC), Lyon, pp 356–357
13. Weiss LM, Gorgan TM, Muller-Hermelink HK, Stein H, Dura T et al (2001) Histocytic and dendritic cell neoplasms. In: Jaffe ES, Harris NL, Stein H, Vardiman JW (eds) World Health Organisation classification of tumors, pathology and genetics of tumors of haematopoietic and lymphoid tissues, 3rd edn. International Agency for Research on Cancer(IARC), Lyon, pp 56–357
14. Weiss LM, Trela MJ, Cleary ML et al (1985) Frequent immunoglobin and T-cell receptor gene rearrangements in histocytic neoplasms. Am J Pathol 121:369–373
