# Supplementary material for: An 8-year-old girl with secondary histiocytic sarcoma with BRAFV600 mutation following T-cell acute lymphoblastic leukemia demonstrating stable disease for 3 years on dabrafenib and trametinib – a case report and literature review
Source: BMC Pediatr. 2025 Mar 8;25:178. doi: 10.1186/s12887-025-05539-2 (PMC11889787; doi:10.1186/s12887-025-05539-2)
Supplement: Supplementary file 6 — Supplementary Material 6 [file 12887_2025_5539_MOESM6_ESM.pdf]

Case report

Histiocytic sarcoma after acute lymphoblastic leukaemia: a common clonal origin

Andrew L Feldman, Caterina Minniti, Mariarita Santi, James R Downing, Mark Raffeld, and Elaine S Jaffe

Lancet Oncol 2004; **5:** 248–50

Histiocytic sarcoma is a rare neoplasm of histiocytic lineage with a poor prognosis. It can be localised (usually to the lymph nodes, skin, or intestinal tract) or disseminated.[1] Cases associated with malignant leukaemia or lymphoma and other neoplasms have been reported, but the nature of this association has not been established. We describe a patient who developed histiocytic sarcoma while on maintenance chemotherapy for acute lymphoblastic leukaemia (ALL). We show identical gene rearrangements in the original leukaemic blasts and the subsequent histiocytic-sarcoma cells, confirming a common clonal origin.

A 14 year-old male was diagnosed with pre-B ALL in January, 2001, after several months of joint and leg pain, weight loss, and fatigue (figure a). Using flow cytometry, the leukaemic blasts expressed TdT, CD19, CD20, CD10, HLA-DR, CD34, CD22, CD38, and cytoplasmic IgM. They were negative for CD11b, CD11c, CD13, and CD33. There was possible weak expression of the myeloid marker CD15, although this finding is not sufficient to classify the case as a biphenotypic leukaemia on the basis of the scoring system of the European Group for the Immunological Classification of Leukaemias[2] and the WHO Classification.[1] Cytogenetic analysis showed no evidence of consistent chromosomal rearrangement. The patient was given chemotherapy based on the Berlin–Frankfurt–Munster regimen for high risk ALL.[3] He responded slowly and received prophylactic cranial irradiation. In October, 2002, while on maintenance chemotherapy (vincristine, methotrexate, 6-mercaptopurine, and prednisone), he developed anaemia and thrombocytopenia. Several bone-marrow aspirates and a bone-marrow biopsy did not show a leukaemic relapse, but showed increased histiocytes with haemophagocytosis. In February, 2003, the patient was noted to have massive splenomegaly (8–10 cm below the left costal margin), lost weight, and developed fatigue and back pain. A CT scan showed multiple masses with poor contrast enhancement in the spleen, a right renal lesion, and a lytic lesion in the 12th rib. The axial skeleton and the pelvic bones had a diffuse lytic and sclerotic pattern. The patient underwent splenectomy for therapeutic and diagnostic purposes.

The spleen weighed 1250 g and had several deep purple nodules of up to 6·0 cm in diameter, some with central necrosis (figure b). Histologically, the nodules were

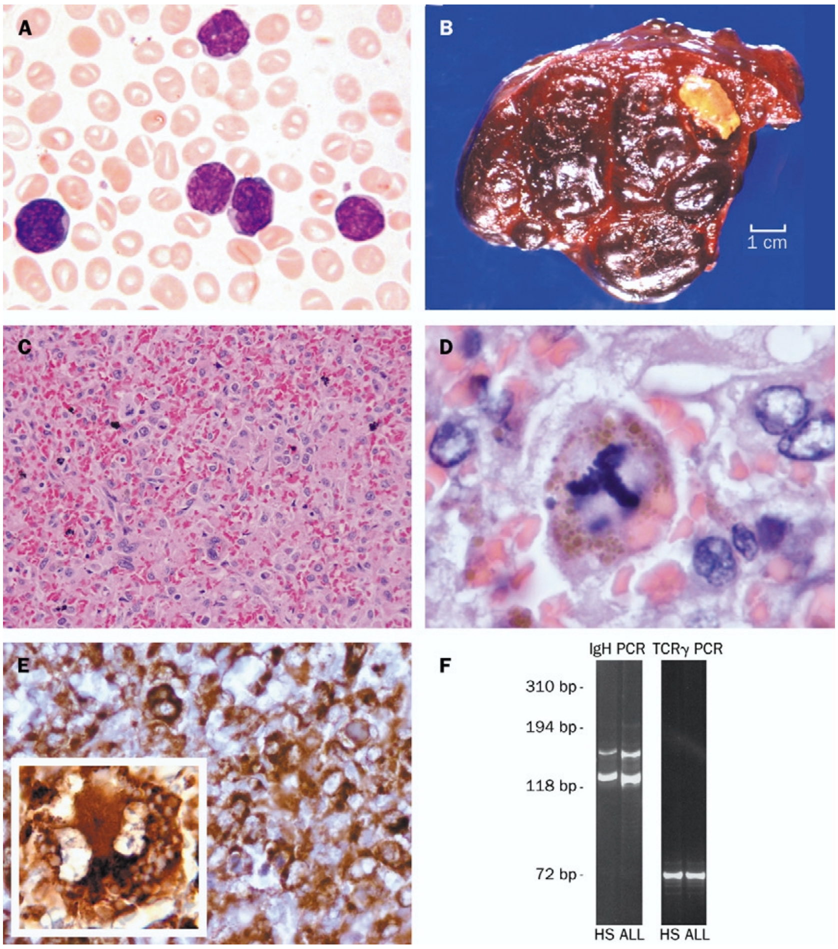

Pre-B ALL at diagnosis, showing lymphoblasts in the peripheral blood (a; Wright stain ×1000). Splenectomy sample with several large nodules, some with necrosis (b). Histiocytic sarcoma involving spleen, showing several large, atypical cells (c; haematoxylin and eosin ×100). Histiocytic sarcoma cell showing atypical, tripolar mitosis and active erythrophagocytosis (d; haematoxylin and eosin ×1000). CD68 immunohistochemical stain showing strong reactivity in histiocytic-sarcoma cells (e; brown reaction product, ×200; inset ×1000). PCR showing identical clonal banding patterns in histiocytic sarcoma and original ALL, by both immunoglobulin (left) and T-cell receptor (right) gene rearrangement studies (f).

For personal use. Only reproduce with permission from The Lancet.

composed of cells with histiocytic morphology, cytological atypia, atypical mitoses, and prominent erythrophagocytosis (figures c and d). Immunohistochemical analyses of the neoplastic cells showed strong positivity for lysozymes and the histiocytic marker CD68 (figure e), and focal positivity for S100. There was multigranular cytoplasmic staining for the B-cell marker CD20, and CD79a and Pax5 were negative. Tumour cells were also negative for markers of residual leukaemia (TdT, CD10), T cells (CD3), Langerhans cells (CD1a), follicular dendritic cells (CD21), and myeloid cells (myeloperoxidase). These findings supported a diagnosis of histiocytic sarcoma.

We did molecular studies on DNA extracted from the patient's original leukaemia and from one of the splenic nodules of histiocytic sarcoma (figure f). PCR amplification with primers to framework region III and the joining region of the immunoglobulin heavy chain gene (IgH) showed identical clonal bands in each sample. PCR amplification with two primer sets for detection of T-cell receptor γ chain (TCRγ) gene rearrangements also showed identical bands in each sample. These results indicate a clonal relation between the two neoplasms. It should be noted that TCRγ gene rearrangements are common in precursor B-cell ALL. RT-PCR assays on frozen tissue from the original leukaemia and from the spleen for translocations associated with ALL were negative in both samples for t(9;22)(BCR/ABL, p190 and p210), t(4;11)(MLL/AF4), t(1;19)(E2A/PBX), and t(12;21)(TEL/AML1).

The patient was given additional chemotherapy (vincristine, cyclophosphamide, daunomycin, methotrexate, etoposide, cytosine arabinoside, and prednisone), however his renal nodule persisted. A core biopsy sample showed histiocytic sarcoma with clonal IgH and TCRγ gene rearrangements identical to those seen in the previous samples. He received an allogeneic bone-marrow transplant in August, 2003.

Histiocytic sarcoma is a rare malignant disorder. The International Lymphoma Study Group[4] have reported the largest series of patients (n=18), of whom seven of 12 with available follow-up died. Most patients were adult males (median age of 46 years). Extranodal presentation occurred in 13 of 18 patients, including involvement of the spleen. Lytic bone lesions and pancytopenia also have been reported.[1] Histologically, the neoplastic cells are large, with abundant cytoplasm and nuclear atypia. Some cells show multinucleation, haemophagocytosis, or both. The shape of the tumour can be strikingly similar to non-histiocytic neoplasms such as diffuse large B-cell lymphoma or anaplastic large-cell lymphoma. As in this case, histiocytic-sarcoma cells strongly express the histiocytic marker CD68, with variable expression of S100. Langerhans and follicular dendritic cell markers are absent. In addition, the mature histiocytic phenotype of the tumour should be verified by showing absence of myeloid markers such as myeloperoxidase.[1]

Histiocytic sarcoma has been reported in association with various other malignant disorders, including ALL.[1] Several earlier reports of this phenomenon preceded the current definition of histiocytic sarcoma (which is based in part on modern immunohistochemical and molecular techniques), and some of these neoplasms would probably be classified differently today. Van der Kwast and colleagues[5] reported a case of a histiocytic neoplasm with IgH gene rearrangement after treatment for T-cell lymphoblastic lymphoma. An identical IgH gene rearrangement was detected in pleural fluid after irradiation of the original lymphoblastic lymphoma, however, a pretreatment sample was not available for comparison. Bouabdallah and colleagues[6] reported a case of a 19-year-old male who presented with a true histiocytic lymphoma 21 months after completing treatment for B-ALL. Both lesions had identical IgH gene rearrangements. The authors suggested that B-ALL cells could act as progenitor cells with the ability to differentiate into other lineages. An experimental model[7] has shown an association between expression of the transcription factor Pax5 and oscillation of cells between B-lymphoid and myeloid lineages. In our case of histiocytic sarcoma, Pax5 staining was negative; however, the unusual finding of cytoplasmic CD20 staining might reflect residual B-cell marker expression.

We present additional molecular evidence that histiocytic sarcoma and ALL have a common clonal origin. We detected identical IgH and TCRγ gene rearrangements in the initial ALL and the subsequent histiocytic sarcoma. The ALL in our case expressed a mature pre-B phenotype, and whether cells showing this amount of maturation could have undergone lineage switching is unclear. Alternatively, both malignant disorders might have resulted from a common abnormality in a precursor cell. Malignant cells with histiocytic differentiation might have been chemoresistant compared with the ALL blasts, only manifesting as histiocytic sarcoma near completion of treatment.

Whether the strict definition of histiocytic sarcoma should include absence of clonal immunoglobulin and TCRγ gene rearrangements is controversial.[1] We emphasise that: histiocytic sarcoma might develop after chemoradiotherapy after treatment for ALL; histiocytic sarcoma that arises in this context might have clonal gene rearrangements; and further molecular studies of both neoplasms should be pursued in such cases to characterise the association with the original leukaemia.

Conflict of interest
None declared.

AF is Clinical Fellow, MR is Staff Physician, and ESJ is Chief of Hematopathology Section; all in the Laboratory of Pathology, National Cancer Institute, Bethesda, MD, USA. CM and MS are Assistant Professors of Paediatrics, George Washington University, Washington, DC, USA and Attending Physicians in the Departments of Hematology–Oncology and Pathology, respectively, at the Children's National Medical Center, Washington, DC, USA. JRD is Member and Chairman, Department of Pathology, St Jude Children's Research Hospital, Memphis, TN, USA.

Correspondence: Dr Andrew L Feldman, Laboratory of Pathology, National Cancer Institute, Building 10, Room 2A33, 10 Center Drive, Bethesda, MD 20892, USA. Tel: +1 (301) 5942945. Fax: +1 (301) 4809488. Email: Andrew_Feldman@nih.gov

For personal use. Only reproduce with permission from The Lancet.

References

1 Jaffe ES, Harris NL, Stein H, Vardiman JW, eds. Pathology and genetics of tumours of haematopoietic and lymphoid tissues. Lyon: IARC Press; 2001.
2 European Group for the Immunological Classification of Leukaemias. The value of c-kit in the diagnosis of biphenotypic acute leukemia. *Leukemia* 1998; **12:** 2038.
3 Nachman J, Sather HN, Gaynon PS, et al. Augmented Berlin-Frankfurt-Munster therapy abrogates the adverse prognostic significance of slow early response to induction chemotherapy for children and adolescents with acute lymphoblastic leukemia and unfavorable presenting features: a report from the Children's Cancer Group. *J Clin Oncol* 1997; **15:** 2222–30.
4 Pileri SA, Grogan TM, Harris NL, et al. Tumours of histiocytes and accessory dendritic cells: an immunohistochemical approach to classification from the International Lymphoma Study Group based on 61 cases. *Histopathology* 2002; **41:** 1–29.
5 van der Kwast TH, van Dongen JJ, Michiels JJ, et al. T-lymphoblastic lymphoma terminating as malignant histiocytosis with rearrangement of immunoglobulin heavy chain gene. *Leukemia* 1991; **5:** 78–82.
6 Bouabdallah R, Abena P, Chetaille B, et al. True histiocytic lymphoma following B-acute lymphoblastic leukaemia: case report with evidence for a common clonal origin in both neoplasms. *Br J Haematol* 2001; **113:** 1047–50.
7 Yu D, Allman D, Goldschmidt MH, et al. Oscillation between B-lymphoid and myeloid lineages in Myc-induced hematopoietic tumors following spontaneous silencing/reactivation of the EBF/Pax5 pathway. *Blood* 2003; **101:** 1950–55.

Advances in research

Adjuvant interferon alfa treatment for patients with malignant melanoma stimulates transporter proteins associated with antigen processing and proteasome activator 28

Faris Abuzahra, Ruth Heise, Sylvia Joussen, Alexandra Dreuw, Hans Merk, Gabriele Zwadlo-Klarwasser, and Jens M Baron

The use of cytokines, especially interferon alfa, for treatment of metastatic melanoma has been assessed in several clinical trials. Although interferon alfa has many immunomodulatory and antiproliferative effects in various malignant disorders, the mechanisms of action in adjuvant therapy for melanoma remain unclear.

We investigated ten patients with UICC (International Union Against Cancer) stage III malignant melanoma and found that expression of transport proteins associated with antigen processing (TAP1 and TAP2) and proteasome activator 28 are upregulated by intravenous adjuvant treatment with 10 million IU/m^2 interferon alfa. This strong stimulatory effect was seen in peripheral blood mononuclear cells both in RNA expression assayed with RT-PCR (figure a), real-time PCR (two-fold to five-fold upregulation of TAP1 mRNA, depending on the patient), and in protein expression assessed with immunohistochemistry (cells labelled with antibody against TAP1 before treatment, figure b; expression of TAP1 after four daily doses of interferon alfa, figure c) and with immunoblotting (figure d).

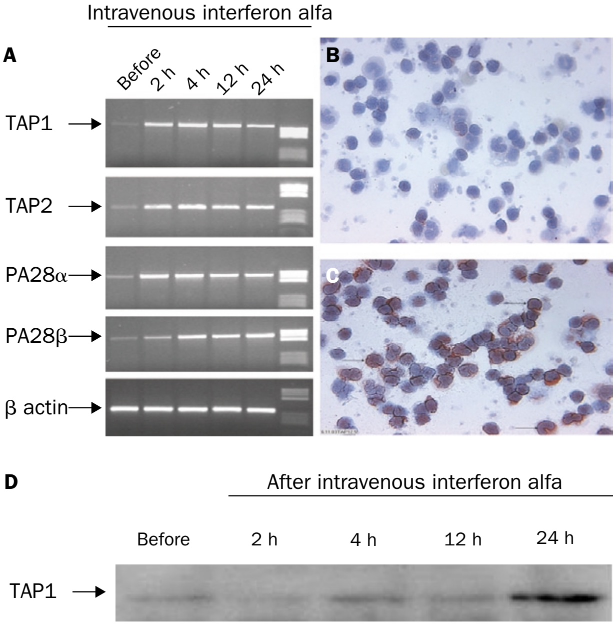

The finding that interferon alfa stimulates cytotoxic effector functions in peripheral blood mononuclear cells of patients receiving intermediate high dose immunotherapy by increasing TAP expression and proteasome activity contributes to the understanding of the immunoregulatory role of type 1 interferons and might help explain the efficacy of interferon alfa therapy. These results also suggest that TAP1 and proteasome activator 28 could be used to monitor therapeutic response and to elucidate the effective dose and duration of interferon alfa treatment.

Correspondence: Dr Jens M Baron, Department of Dermatology, University Hospital RWTH Aachen, Pauwelsstrasse 30, D-52074 Aachen, Germany. Tel: +49 (0) 241 8085113. Fax: +49 (0) 241 8082413. Email: JensMalte.Baron@post.rwth-aachen.de

For personal use. Only reproduce with permission from The Lancet.
