# Supplementary material for: An 8-year-old girl with secondary histiocytic sarcoma with BRAFV600 mutation following T-cell acute lymphoblastic leukemia demonstrating stable disease for 3 years on dabrafenib and trametinib – a case report and literature review
Source: BMC Pediatr. 2025 Mar 8;25:178. doi: 10.1186/s12887-025-05539-2 (PMC11889787; doi:10.1186/s12887-025-05539-2)
Supplement: Supplementary file 7 — Supplementary Material 7 [file 12887_2025_5539_MOESM7_ESM.pdf]

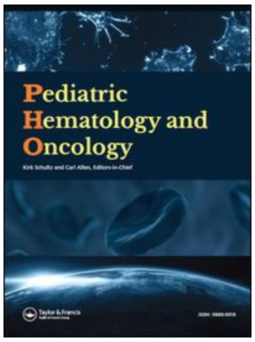

Pediatric Hematology and Oncology

ISSN: (Print) (Online) Journal homepage: https://www.tandfonline.com/loi/ipho20

Short-term response to alemtuzumab in CD52-positive secondary histiocytic sarcoma in a child: Is it time to consider new targets?

Elvis Terci Valera , María Sol Brassesco , Maristella Bergamo Francisco dos Reis , Gilberto Maggioni , Renato Luiz Guerino-Cunha , Carlos Eduardo Grecco , Jorge Elias Jr. , Mery Kato & Luiz Gonzaga Tone

To cite this article: Elvis Terci Valera , María Sol Brassesco , Maristella Bergamo Francisco dos Reis , Gilberto Maggioni , Renato Luiz Guerino-Cunha , Carlos Eduardo Grecco , Jorge Elias Jr. , Mery Kato & Luiz Gonzaga Tone (2020): Short-term response to alemtuzumab in CD52-positive secondary histiocytic sarcoma in a child: Is it time to consider new targets?, Pediatric Hematology and Oncology, DOI: 10.1080/08880018.2020.1811438

To link to this article: https://doi.org/10.1080/08880018.2020.1811438

View supplementary material

Published online: 08 Sep 2020.

Submit your article to this journal

View related articles

View Crossmark data

Full Terms & Conditions of access and use can be found at
https://www.tandfonline.com/action/journalInformation?journalCode=ipho20

PEDIATRIC HEMATOLOGY AND ONCOLOGY
https://doi.org/10.1080/08880018.2020.1811438

Taylor & Francis
Taylor & Francis Group

Check for updates

LETTER

Short-term response to alemtuzumab in CD52-positive secondary histiocytic sarcoma in a child: Is it time to consider new targets?

Elvis Terci Valera[a], María Sol Brassesco[b], Maristella Bergamo Francisco dos Reis[a], Gilberto Maggioni[c], Renato Luiz Guerino-Cunha[d], Carlos Eduardo Grecco[d], Jorge Elias Jr.[e], Mery Kato[f], and Luiz Gonzaga Tone[a]

[a]Department of Pediatrics, Ribeirão Preto Medical School, University of São Paulo, Ribeirão Preto, Brazil; [b]Department of Biology, Faculty of Philosophy, Sciences and Letters at Ribeirão Preto, University of São Paulo, Brazil; [c]Laboratório Dr. Prates, Ribeirão Preto, Brazil; [d]Bone Marrow Transplantation and Cellular Therapy Unit, Department of Medical Imaging, Hematology and Clinical Oncology, Ribeirão Preto Medical School, University of São Paulo Brazil; [e]Department of Medical Imaging, Hematology and Clinical Oncology, Ribeirão Preto Medical School, University of São Paulo Brazil; [f]Diagnostic of Nuclear Medicine - DIMEN - Ribeirão Preto, Brazil

To the Editor

Second malignant neoplasms (SMN) are well-recognized long-term health problems in individuals diagnosed and treated for acute lymphoblastic leukemia (ALL) at infancy and adolescence. Among them, secondary acute myeloid leukemia (AML), myelodysplastic syndrome (MDS) and central nervous system (CNS) tumors are well documented.[1] Histiocytosis, and in particular, histiocytic sarcomas (HS), are very rarely described following ALL.

Histiocytic and macrophage-dendritic neoplasms are very uncommon tumors with variable clinical behavior. Current classification proposal stratifies these lesions into five main groups: L (Langerhans), C (Cutaneous and mucocutaneous histiocytosis), R (Rosai-Dorfman Disease and miscellaneous noncutaneous, non-Langerhans cell histiocytosis), M (Malignant histiocytosis) and H (Hemophagocytic lymphohistiocytosis and macrophage activation syndrome).[2] A large spectrum of histiocytic lesions has been described in association with leukemia in children.[3] These lesions range from benign, locally aggressive, to highly malignant tumors. HS is a subtype of malignant histiocytosis considered a very aggressive and potentially lethal end of this spectrum where genetic information and new insights supporting precision therapies are warranted. Owing to the paucity of clinical and genetic information on pediatric cases of secondary HS following ALL, our brief report aimed to add a thorough description on both clinical and genetic findings of this association in a child. Clinical experience with targeted-therapy as a rescue regimen using alemtuzumab is also described. In addition, we proposed to gather and to explore the most frequent mutations and leading cell pathways involved in this SMN described in pediatric cases to date.

A six-year-old male was consulted elsewhere due to bruising and pallor. Complete blood cell count revealed normal levels of hemoglobin (12.5 g/dL), normal platelet count (210,000/mm^3) and leukocytosis (160,000/mm^3); blood smear depicted 75% of circulating blasts. Bone

CONTACT Elvis Terci Valera etvalera@hcrp.usp.br FMRP-USP, Avenida Bandeirantes 3900 Hospital das Clínicas - Bloco G; 1° Andar. Laboratório de Pediatria. CEP: 14049-900, Ribeirão Preto/SP, Brazil; Maria Sol Brassesco solbrassesco@usp.br Department of Biology, Faculty of Philosophy, Sciences and Letters at Ribeirão Preto, University of São Paulo, Brazil.
*These authors contributed equally to this work.
Supplemental data for this article can be accessed at https://doi.org/10.1080/08880018.2020.1811438
© 2020 Taylor & Francis Group, LLC

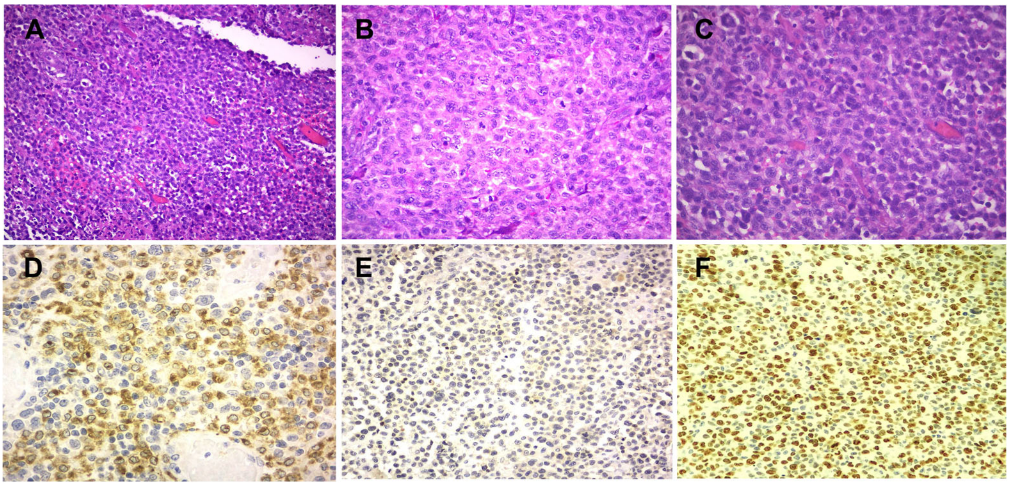

Figure 1. Hematoxylin and Eosin staining (HE; 100X magnification - Figure A; HE; 400X magnification-Figures B and C) showing a densely cellular neoplasia, consisting of cells of histiocytic pattern with relevant nuclear pleomorphism. (D) IHC for CD68 (histiocytic marker; 400X magnification) depicting frequent positivity in neoplastic cells. (E) CD52 IHC staining (400X magnification) with diffuse positivity in neoplastic cells. (F) IHC for Ki-67 (400X magnification) with strong positivity in about 90% of neoplastic cells. Abbreviations: H&E: hematoxylin and eosin; IHC: Immunohistochemistry.

marrow aspirate (BMA) showed massive lymphoblast infiltration. Immunophenotype revealed positivity on leukemic cells for terminal deoxynucleotidyl transferase (TdT), CD45, CD2, cCD3, CD5, CD7, CD8, TDT, CD10, CD38 and CD79a; myeloperoxidase was negative. Final diagnosis was high-risk T-cell ALL; CNS was not involved. Patient was treated according to the Pediatric Brazilian ALL protocol.[4] He achieved complete remission, with negative minimal residual disease by flow-cytometry at the end of induction. During maintenance, two reddish-elevated lesions measuring around 2 cm were observed at his face and neck. A skin biopsy was performed and pathological examination revealed infiltration by Langerhans-cell histiocytosis, with positive immunostaining for S-100/CD1a and low Ki-67 labeling (around 2% of tumor cells). Lesions resolved spontaneously while the patient was taking steroids and 6-mercaptopurine. Patient ended ALL treatment without major complications. Six months later he experienced intense pain at his left leg, associated with left inguinal adenopathy. Positron emission tomography scan (PET-CT) showed multiple abdominal lymph nodes. Tumor biopsy revealed a densely cellular neoplasm consisting of cells of histiocytic pattern with intense nuclear pleomorphism. Immunohistochemistry was positive for CD52, CD1a and CD68; mitoses were very frequent (Ki-67 = 90%; Figure 1A–F). The child was diagnosed with metastatic HS. A new BMA was performed to exclude ALL relapse or bone marrow infiltration by HS and resulted normal. Initial treatment consisted of four cycles of Cyclophosphamide, Doxorubicin, Prednisone and Vincristine (CHOP) with no objective disease response at reassessment. Due to CD52 positivity on tumor cells, a targeted treatment with alemtuzumab was offered.[5] The drug was delivered by intravenous infusion on 5-day cycles at increasing doses as follows (patient weight: 40 kg): 2 mg (D1); 4 mg (D2); 5 mg (D3); and 10 mg on D4 and D5. Methylprednisolone was used prior to alemtuzumab infusions on D1-3, to avoid allergic reactions. Pain centered at tumor locations (inguinal region and lower abdomen) completely disappeared after the first cycle, and opioids were withdrawn. Objective clinical response was initially observed by computed tomography (CT). Abdominal CT prior to the

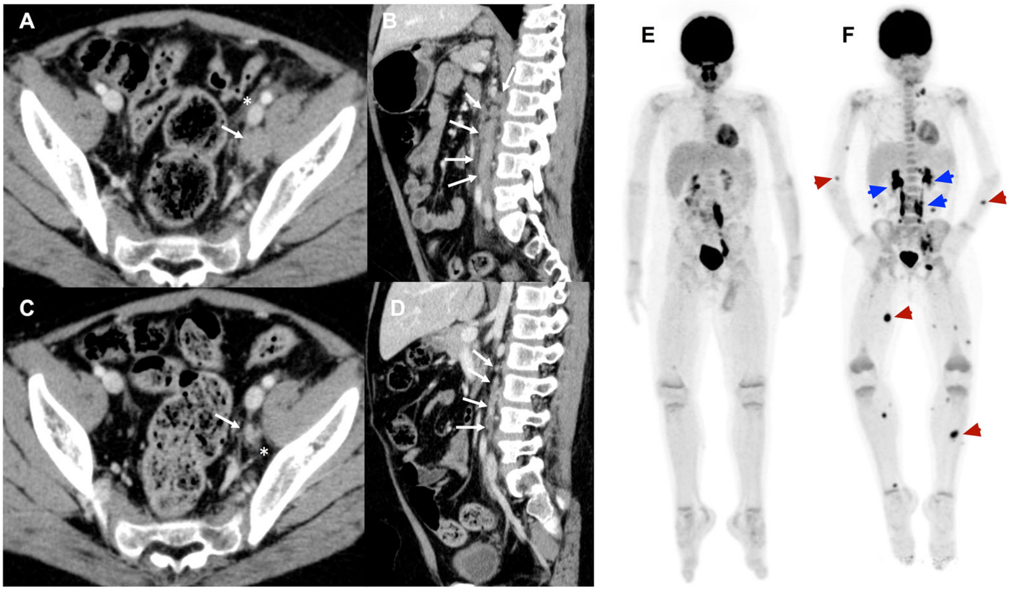

Figure 2. Axial computed tomography and left parasagittal multiplanar reconstruction obtained before (A and B, respectively) and after 2 cycles of alemtuzumab (C and D, respectively). Axial CT shows an enlarged lymph node in the left external iliac chain with irregular contours (white arrow in A). In addition, there is densification of fat around the left iliac vessels (*). Enlarged and coalescent lymph nodes are seen in the retroperitoneum (white arrows in B). After 2 cycles of alemtuzumab, there was a marked reduction in the size of the lymph node in the left external iliac chain (white arrow in C), with onset of fat densification around it (*). Marked reduction in the increase in retroperitoneal lymph nodes was observed (white arrows in D). (E) Positron emission tomography/Maximal intensity projection (MIP) shows sites of tumor involvement, visualized as areas of increase Fluorodeoxyglucose (FDG) uptake in abdomen, inguinal lymphadenopathy and distal right femur. (F) the same MIP acquisition shows extensive sites of tumor involvement visualized as areas of increased FDG uptake at cervical, mediastinal and abdominal (blue arrowheads) lymphadenopathy and several novel large subcutaneous nodules (red arrowheads).

CD52-directed therapy showed multiple enlarged lymph nodes in the retroperitoneum and in the iliac and inguinal chains, with a larger lymph node in the left external iliac chain showing irregular contours (Figure 2A). In addition, it was possible to identify fat densification adjacent to the affected lymph nodes, with a coalescent aspect in several places, more evident in the retroperitoneum (Figure 2B). After 2 cycles of alemtuzumab, the follow-up CT scan showed partial response, with marked reduction in the dimensions of multiple lymph nodes, including complete remission at various levels (Figure 2C,D). Autologous bone marrow transplantation (ABMT) was indicated, but patient failed to mobilize peripheral CD34 stem cell with G-CSF. A third cycle of alemtuzumab was offered but new PET-CT revealed that disease rapidly progressed to the mediastinum, upper thoracic lymph nodes, abdomen and skin (Figure 2E,F). Third-line chemotherapy with Cladribine/Cytarabine[6] was attempted. After 2 cycles the child experienced partial response and was treated with haploidentical bone marrow transplantation. The patient eventually died (day+13) due to complications related to Sinusoidal Obstruction Syndrome (SOS).

Next generation sequencing (NGS) of a targeted set of genes evaluated at HS tumor sample (FoundationOne_Heme®; complete list of genes available at https://assets.ctfassets.net/vhribv12lmne/zBxaQC12cScqgsEk8seMO/a70860fea48927c7ff8b70e90c292182/F1H_Technical

Table 1. List of published cases of histiocytic sarcoma (HS) secondary to acute leukemia occurring in children ad adolescents up to 18 years of age.

Author and reference number	Patient age (y)	Primary tumor	Genetic background
Soslow et al.[6]	8	preB-ALL	ND
	6	preB-ALL	ND
Dalle et al.[7]	4	T-ALL	ND
Wongchanchailert and Laosombat[8]	8	preB-ALL	ND
Feldman et al.[9]	14	preB-ALL	ND
Castro et al.[2]	5	T-ALL	*CDKN2A* loss
	15	preB-ALL	ND
	7	preB-ALL	t(8;14)
	3	T-ALL	ND
Kumar et al.[11]	4	preB-ALL	*CDKN2A* loss
Ganapule et al.[12]	4	T-ALL	ND
Alten et al.[13]	6	cortical T-ALL	*CDKN2A* loss
Bleeke et al.[14]	10	cortical T-ALL	*CDKN2A* loss
	11	biphenotypic/bilineal AL	*CDKN2A* loss, *KRAS* (p.A66fs)
Venkataraman et al.[15]	0.4	T-ALL	*CDKN2A* loss, *BRAF*V600E, (8;14)(q24;q11.2) with *TRA-MYC* rearrangement

Information on patients' age, subtype of acute leukemia prior to HS and genetic information on HS (if available from the original article) are summarized.
ND: not described; y: year.

Information.pdf) revealed somatic mutations in *NRAS* (G12C), *STK11* (W332*) and *TP53* (V173M), along with amplification of *CDK6* (cyclin dependent kinase 6), *HGF* (hepatocyte growth factor), *NFKBIA* (NFKB inhibitor alpha) and *RAD21* (cohesin complex component). Most importantly, the specimen showed loss of the tumor suppressor *CDKN2A*/B – [p16INK4a and p14ARF (exons 2–3)]. The complete list of deleterious variants (lines 1 to 8) and of variants of unknown significance (VUS – lines 9 to 20) is described at Supporting Information Table S1. Additionally, data search on PubMed (https://www.ncbi.nlm.nih.gov/pubmed) applying the uniterms "Histiocytic", "Sarcoma", "Leukemia" and "Children", (search period: Jan 2000 – Feb 2020) retrieved 14 articles. After careful article reading, a total of 15 cases of secondary HS following ALL in children and adolescents (up to 18-year old) were obtained.[3,6–16] Since we were investigating HS as a SMN following leukemia, a case of synchronous HS and B-cell ALL was not included.[17] Table 1 summarizes these studies, along with clinical and genetic information. Median age of leukemia diagnosis was 7.02 years. Acute leukemia subtypes related to HS were T-cell ($n = 7$), Pre-B cell ($n = 7$) and biphenotypic/bilineal in one case. Interestingly, seven out of eight cases with genetic information showed *CDKN2A* loss. Other somatic genetic features described in this cohort included *KRAS* (p.A66fs), two t(8;14) (with one mapped at (q24;q11.2) with *TRA-MYC* rearrangement) and *BRAFV600E* mutation.

Besides contributing with another pediatric case of HS secondary to a T-cell ALL, this brief report compiles for the first time clinical and genetic data on this association in the pediatric population. Clinical treatment options are scarce for children and adolescents experiencing secondary HS. Frequent rescue treatments applied in this scenario for adult patients are CHOP or ProMACE-MOPP chemotherapy regimens.[18] Dose-escalated CHOPP associated to Etoposide was also a successful treatment approach described in literature.[19] Other successful treatment strategies for HS both in children and adults include chemotherapy combination with Cladribine, Cytarabine, G-CSF and Mitoxantrone (CLAM-G),[20] and Cladribine/Cytarabine regimen.[6] The majority of these schedules aim to serve as a bridge for stem cell transplantation,

either using autologous or allogeneic stem-cell sources.[21,22] Additionally, Thalidomide, an extensively used drug with antiangiogenic properties, was used in HS mainly following hematopoietic stem-cell transplantation, with favorable results.[8,23] Alemtuzumab, has been used to treat several different subtypes of refractory histiocytic diseases, including HS.[5,24,25] This monoclonal anti-CD52 antibody may also be indicated to treat severe cases of multiple sclerosis[26] and chronic lymphocytic leukemia.[27] Secondary T-cell depletion and lymphopenia are common side effects of alemtuzumab treatment, which may lead to life-threatening viral and fungal opportunistic infections.[28] Our patient experienced both fungal rhinosinusitis (*Aspergillus*) and viral infection (HHV6 reactivation), leading to the necessity of prolonged hospitalization to treat these complications. Also, long-term side effects related to autoimmune diseases were observed.[29] Besides anti-CD52, different alternatives of targeted therapy to treat HS remain exceptional. Case reports using immunotherapy with nivolumab were described with variable and conflicting clinical outcomes.[30,31]

In addition to these well-recognized treatment strategies in HS, literature review demonstrates a clear association between genetic alterations within the RAS/MAPK pathway and histiocytic tumors, mostly in adults.[11,32] Additionally, *NOTCH1* point mutations were observed in two out of five cases of primary HS in adults.[33] Despite this association being largely accepted for both primary and secondary HS in adults, it remains unclear if children and adolescents share the same genetic mechanisms. Activating *KRAS* and *NRAS* mutations have also been observed in this setting.[34,11] However, the single most frequently altered gene in HS is the tumor-suppressor *CDKN2A*.[35]

It is widely recognized that secondary HS harbors the same molecular or cytogenetic abnormalities as the primary malignancy.[14,15] Secondary malignancies are relatively common in leukemia patients, however, HS affecting children after AL remains exceptional, and most of those with genetic descriptions present loss of *CDKN2A/B*. Both proteins encoded by this gene (p16INK4a and p15INK4b) negatively regulate cyclin/CDK-4/6 complexes to block cell division during the G1/S phase of the cell cycle.[35] Interestingly, deletion of *CDKN2A*/B also occurs in 21% of childhood B-cell precursor ALL and 50% of pediatric T-ALL.[36] Moreover, experimental models with double-mutant mice lacking both copies of *CDKN2A* and one copy of the tumor suppressor gene *PTEN* present a clear predisposition for the development of HS[37] indicating a cooperative action between these pathways, as seen for other tumors.[38]

As in the case of adults,[33,34] mutations involving the MAPK cascade have also been found in childhood secondary HS by us and others.[15] This cascade is a convergent signaling node receiving input from numerous internal (metabolic stress, DNA damage pathways and altered protein concentrations) and external (growth factors, cell-to-cell communication, extracellular matrix) stimuli.[39] Mutations affecting this pathway may occur upstream in membrane receptor genes (*RET*, *EPHA5*, *ERBB2*, in our index), in signal transducers (*HRAS* and *NRAS*, as seen by us and others), regulatory partners and in downstream kinases belonging to pathway itself (as seen for *BRAF*).[16] Such alterations generally result in abnormal activation or gain-of-function mutations that play key roles in tumor establishment and maintenance.

In summary, secondary HS in children and adolescents is a rare and difficult-to-treat hematologic malignancy, which lacks a well-established clinical protocol. Although complete and sustained responses to alemtuzumab in HS are described in literature, clinical outcome with this treatment approach may not be uniform, even for CD52 positive HS disease. Collectively, our data warrants the investigation of *CDKN2A*/B deletions, as well as *MAPK* mutations in children and adolescents with HS as SMN. Aberrantly activated MAPK pathway may be a promising avenue to target this tumor. A few successful applications of precision medicine in adults[40] (trametinib for *MAP2K1*-mutant) and children (Dabrafenib/trametinib for *BRAFV600E*-mutant HS)[16] were recently reported, pharmacologically

exploring abnormally activated MAPK pathway in HS with successful short-term outcomes. Yet, data on translational treatment for this orphan disease are still scarce, as well as long-term effects of these new approaches.

Availability of data and materials

All data generated from this case description are presented in this article and supporting information material.

Author contribution statement

E. T. V planned and conducted data analysis and drafted the manuscript; M. S. B wrote and organized the data, created the figures/tables and edited and finalized the manuscript. M. K. and J. E. J. prepared CT and PET-CT images. M. B. R., G. M., R. C., C. E. G. and L. G. T revised the text for important intellectual content. All authors critically read and approved the final manuscript.

Ethics approval and consent to participate

Parents gave written informed consent for genetic studies and for the publication of data from this case report (available upon request). This case report was also IRB approved (#3.897.057)

Competing interests

The authors declare that they have no competing interests.

Conflict of interest

The authors declare no conflict of interest. All authors read and approved the final version of the manuscript.

Funding

FAEPA (Fundação de Amparo ao Ensino, Pesquisa e Assistência/HCFMRP-USP).

References

1. Schmiegelow K, Levinsen MF, Attarbaschi A, et al. Second malignant neoplasms after treatment of childhood acute lymphoblastic leukemia. *J Clin Oncol*. 2013;31(19):2469–2476. doi:10.1200/JCO.2012.47.0500.
2. Emile JF, Abla O, Fraitag S, et al. Revised classification of histiocytoses and neoplasms of the macrophage-dendritic cell lineages. *Blood*. 2016;127(22):2672–2681. doi:10.1182/blood-2016-01-690636.
3. Castro ECC, Blazquez C, Boyd J, et al. Clinicopathologic features of histiocytic lesions following ALL, with a review of the literature. *Pediatr Dev Pathol*. 2010;13(3):225–237. doi:10.2350/09-03-0622-OA.1.
4. Brandalise SR, Viana MB, Pinheiro VRP, et al. Shorter maintenance therapy in childhood acute lymphoblastic leukemia: the experience of the prospective, randomized Brazilian GBTLI ALL-93 Protocol. *Front Pediatr*. 2016;4:110. Published 2016 Oct 17. doi:10.3389/fped.2016.00110.

5. Shukla N, Kobos R, Renaud T, et al. Successful treatment of refractory metastatic histiocytic sarcoma with alemtuzumab. *Cancer*. 2012;118(15):3719–3724. doi:10.1002/cncr.26712.
6. Iwabuchi H, Kawashima H, Umezu H, et al. Successful treatment of histiocytic sarcoma with cladribine and high-dose cytosine arabinoside in a child. *Int J Hematol*. 2017;106(2):299–303. doi:10.1007/s12185-017-2202-8.
7. Soslow RA, Davis RE, Warnke RA, Cleary ML, Kamel OW. True histiocytic lymphoma following therapy for lymphoblastic neoplasms. *Blood*. 1996;87(12):5207–5212. doi:10.1182/blood.V87.12.5207.bloodjournal87125207.
8. Dalle JH, Leblond P, Decouvelaere A, et al. Efficacy of thalidomide in a child with histiocytic sarcoma following allogeneic bone marrow transplantation for T-ALL. *Leukemia*. 2003;17(10):2056–2057. doi:10.1038/sj.leu.2403075.
9. Wongchanchailert M, Laosombat V. True histiocytic lymphoma following acute lymphoblastic leukemia. *Med Pediatr Oncol*. 2003;40:51–53. doi:10.1002/mpo.10056.
10. Feldman AL, Minniti C, Santi M, Downing JR, Raffeld M, Jaffe ES. Histiocytic sarcoma after acute lymphoblastic leukaemia: a common clonal origin. *Lancet Oncol*. 2004;5(4):248–250. doi:10.1016/S1470-2045(04)01428-7.
11. Choi SM, Andea AA, Wang M, et al. KRAS mutation in secondary malignant histiocytosis arising from low grade follicular lymphoma. *Diagn Pathol*. 2018;13(1). doi:10.1186/s13000-018-0758-0.
12. Kumar R, Khan SP, Joshi DD, Shaw GR, Ketterling RP, Feldman AL. Pediatric histiocytic sarcoma clonally related to precursor B-cell acute lymphoblastic leukemia with homozygous deletion of CDKN2A encoding p16INK4A. *Pediatr Blood Cancer*. 2011;56:307–310. doi:10.1002/pbc.22810.
13. Ganapule AP, Gupta M, Kokil G, Viswabandya A. Histiocytic sarcoma with acute lymphoblastic leukemia a rare association: case report and literature review. *Indian J Hematol Blood Transfus*. 2014;30(S1):305–308. doi:10.1007/s12288-014-0375-3.
14. Alten J, Klapper W, Leuschner I, et al. Secondary histiocytic sarcoma may cause apparent persistence or recurrence of minimal residual disease in childhood acute lymphoblastic leukemia. *Pediatr Blood Cancer*. 2015;62(9):1656–1660. doi:10.1002/pbc.25523.
15. Bleeke M, Johann P, Gröbner S, et al. Genome-wide analysis of acute leukemia and clonally related histiocytic sarcoma in a series of three pediatric patients. *Pediatr Blood Cancer*. 2020;67(2). doi:10.1002/pbc.28074.
16. Venkataraman V, Massoth LR, Sullivan RJ, Friedmann AM. Secondary histiocytic sarcoma with BRAFV600E mutation after T-cell acute lymphoblastic leukemia in a very young child with dramatic response to dabrafenib and trametinib. *Pediatr Blood Cancer*. 2020;67(5):e28200. doi:10.1002/pbc.28200.
17. Pani KC, Yadav M, Kumar S, Agrawal V. Extranodal histiocytic sarcoma in a child with acute lymphoblastic leukemia: cytomorphological features of a rare entity with brief review of literature. *Indian J Pathol Microbiol*. 2018;61(2):278. doi:10.4103/IJPM.IJPM_67_16.
18. Hornick JL, Jaffe ES, Fletcher CDM. Extranodal histiocytic sarcoma: clinicopathologic analysis of 14 cases of a rare epithelioid malignancy. *Am J Surg Pathol*. 2004.
19. Tsujimura H, Miyaki T, Yamada S, et al. Successful treatment of histiocytic sarcoma with induction chemotherapy consisting of dose-escalated CHOP plus etoposide and upfront consolidation auto-transplantation. *Int J Hematol*. 2014;100(5):507–510. doi:10.1007/s12185-014-1630-y.
20. Tomlin J, Orosco RK, Boles S, et al. Successful treatment of multifocal histiocytic sarcoma occurring after renal transplantation with cladribine, high-dose cytarabine, G-CSF, and mitoxantrone (CLAG-M) followed by allogeneic hematopoietic stem cell transplantation. *Case Rep Hematol*. 2015;2015:728260. doi:10.1155/2015/728260.
21. Zeidan A, Bolaños-Meade J, Kasamon Y, et al. Human leukocyte antigen-haploidentical hematopoietic stem cell transplant for a patient with histiocytic sarcoma. *Leuk Lymphoma*. 2013;54(3):655–657. doi:10.3109/10428194.2012.717082.
22. Abu-Sanad A, Warsi A, Michel RP, et al. Long-term remission after autologous stem-cell transplantation for relapsed histiocytic sarcoma. *Curr Oncol*. 2012;19(4):e289–e291. doi:10.3747/co.19.964.
23. Abidi MH, Tove I, Ibrahim RB, Maria D, Peres E. Thalidomide for the treatment of histiocytic sarcoma after hematopoietic stem cell transplant. *Am J Hematol*. 2007;82(10):932–933. doi:10.1002/ajh.20913.

24. Marsh RA, Allen CE, Mcclain KL, et al. Salvage therapy of refractory hemophagocytic lymphohistiocytosis with alemtuzumab. *Pediatr Blood Cancer*. 2013;60(1):101–109. doi:10.1002/pbc.24188.
25. Abid MB, Wadera K, Bird JM, Pawade J, Marks DI. Alemtuzumab-based therapy for Secondary Malignant Histiocytosis arising from Pre-B-ALL. *Leuk Res Rep*. 2017;9:5–8. doi:10.1016/j.lrr.2017.11.003.
26. Jones JL, Coles AJ. Mode of action and clinical studies with alemtuzumab. *Exp Neurol*. 2014;262:37–43. doi:10.1016/j.expneurol.2014.04.018.
27. Alinari L, Lapalombella R, Andritsos L, Baiocchi RA, Lin TS, Byrd JC. Alemtuzumab (Campath-1H) in the treatment of chronic lymphocytic leukemia. *Oncogene*. 2007;26(25):3644–3653. doi:10.1038/sj.onc.1210380.
28. Buonomo AR, Zappulo E, Viceconte G, Scotto R, Borgia G, Gentile I. Risk of opportunistic infections in patients treated with alemtuzumab for multiple sclerosis. *Expert Opin Drug Saf*. 2018;17(7):709–717. doi:10.1080/14740338.2018.1483330.
29. Cossburn M, Pace AA, Jones J, et al. Autoimmune disease after alemtuzumab treatment for multiple sclerosis in a multicenter cohort. *Neurology*. 2011;77(6):573–579. doi:10.1212/WNL.0b013e318228bec5.
30. Bose S, Robles J, McCall CM, et al. Favorable response to nivolumab in a young adult patient with metastatic histiocytic sarcoma. *Pediatr Blood Cancer*. 2019;66(1):e27491. doi:10.1002/pbc.27491.
31. Voruz S, Martins F, Cairoli A, et al. Comment on "MEK inhibition with trametinib and tyrosine kinase inhibition with imatinib in multifocal histiocytic sarcoma. *Haematologica*. 2018;103(3):e130. doi:10.3324/haematol.2017.186932.
32. Egan C, Nicolae A, Lack J, et al. Genomic profiling of primary histiocytic sarcoma reveals two molecular subgroups. *Haematologica*. 2020;105(4):951–960. doi:10.3324/haematol.2019.230375.
33. Hung YP, Lovitch SB, Qian X. Histiocytic sarcoma: new insights into FNA cytomorphology and molecular characteristics. *Cancer Cytopathol*. 2017;125(8):604–614. doi:10.1002/cncy.21851.
34. Shanmugam V, Griffin GK, Jacobsen ED, Fletcher CDM, Sholl LM, Hornick JL. Identification of diverse activating mutations of the RAS-MAPK pathway in histiocytic sarcoma. *Mod Pathol*. 2019;32(6):830–843. doi:10.1038/s41379-018-0200-x.
35. Cánepa ET, Scassa ME, Ceruti JM, et al. INK4 proteins, a family of mammalian CDK inhibitors with novel biological functions. *TBMB*. 2007;59(7):419–426. doi:10.1080/15216540701488358.
36. Sulong S, Moorman AV, Irving JAE, et al. A comprehensive analysis of the CDKN2A gene in childhood acute lymphoblastic leukemia reveals genomic deletion, copy number neutral loss of heterozygosity, and association with specific cytogenetic subgroups. *Blood*. 2009;113(1):100–107. doi:10.1182/blood-2008-07-166801.
37. Carrasco DR, Fenton T, Sukhdeo K, et al. The PTEN and INK4A/ARF tumor suppressors maintain myelolymphoid homeostasis and cooperate to constrain histiocytic sarcoma development in humans. *Cancer Cell*. 2006;9(5):379–390. doi:10.1016/j.ccr.2006.07.012.
38. Hsieh R, Nico MMS, Coutinho-Camillo CM, Buim ME, Sangueza M, Lourenço SV. The CDKN2A and MAP kinase pathways: molecular roads to primary oral mucosal melanoma. *Am J Dermatopathol*. 2013;35(2):167–175. doi:10.1097/DAD.0b013e31825fa1f6.
39. Burotto M, Chiou VL, Lee JM, Kohn EC. The MAPK pathway across different malignancies: a new perspective. *Cancer*. 2014;120(22):3446–3456. doi:10.1002/cncr.28864.
40. Gounder MM, Solit DB, Tap WD. Trametinib in histiocytic sarcoma with an activating MAP2K1 (MEK1) mutation. *N Engl J Med*. 2018;378(20):1945–1947. doi:10.1056/NEJMc1511490.
