# Supplementary material for: An 8-year-old girl with secondary histiocytic sarcoma with BRAFV600 mutation following T-cell acute lymphoblastic leukemia demonstrating stable disease for 3 years on dabrafenib and trametinib – a case report and literature review
Source: BMC Pediatr. 2025 Mar 8;25:178. doi: 10.1186/s12887-025-05539-2 (PMC11889787; doi:10.1186/s12887-025-05539-2)
Supplement: Supplementary file 8 — Supplementary Material 8 [file 12887_2025_5539_MOESM8_ESM.pdf]

Efficacy of thalidomide in a child with histiocytic sarcoma following allogeneic bone marrow tranplantation for T-ALL

Leukemia (2003) **17,** 2056–2057. doi:10.1038/sj.leu.2403075

TO THE EDITOR

A 4-year-old boy was admitted to treat a *de novo* T-ALL. The initial biologic pattern of disease showed high WBC (533×10^9/l with 90% of blast cells), anemia (Hb: 72g/l) and thrombocytopenia (60×10^9/l). Cytogenetic studies revealed a complex abnormality with 45 XY del 6(q21,q25), dic(9;12)(p13;p11) t(8;14)(q24;q11). Molecular studies revealed variable chain markers $V\gamma_1 J_1 J_2+$ and $V\delta_1 J\delta_1+$. The patient was treated according to the high-risk arm of the EORTC-CLCG 58951 protocol. The disease proved to be resistant at the corticosteroid preinduction phase, at day 8 of treatment as defined by the protocol. Complete remission was obtained after induction but minimal residual disease remained higher than 1%, that is, more than 1% of cells still presented initial molecular markers. Two related bone marrow donors (HLA match 10/10) were identified: his twin and his father. Owing to worse results obtained with the graft from twin, he was transplanted from his father. In spite of graft-versus-host disease (GVHD) prophylaxis including cyclosporin A and a short-course of methotrexate, he developed a severe cutaneous acute GVHD at D60 after transplant. He was then given high-dose corticosteroids (5 mg/kg daily for 7 days) associated with mycophenolate mofetyl (500 mg b.i.d.) tapered after 3 months at full dose. Half-month after interruption of his immunosuppressive therapy (and 9 months after transplantation), he presented with severe weight loss associated with abdominal pain and a right tibial spontaneous fracture. Pancorporal CT scan and MRI revealed diffuse marrow infiltration, a gastrointestinal mass, pulmonary nodes and several subpleural masses. Surgery was performed. Macroscopically, the small intestine wall was entirely involved by an ulcerated tumor measuring $2.5 \times 1.5\,cm^2$. Hematoxylin- and eosin-stained sections revealed a diffuse noncohesive proliferation of medium to large cells. The cytoplasm was usually abundant, eosinophilic and occasionally foamy and the chromatin was vesicular. Mitosis and atypia were frequent with highly irregular nuclear outlines (Figure 1). Hemophagocytosis occurred occasionally in the neoplastic cells. Immunohistochemical analysis showed that malignant cells were stained with anti-CD45, antilysozyme, anti-CD68 (KP1 clone) and antivimentin antibodies (Figure 2). Immunostaining with anti-CD1a, anti-PS100, CNA-42 (FDRC), anti-CD30, anti-CD15, anti-MPO, anti-CD3, anti-CD20, anti-CD79a, anti-EMA and anti-ALK antibodies was negative. These data were consistent with the diagnosis of histiocytic sarcoma. Bone marrow biopsy showed a diffuse infiltration by malignant cells. EBV genome was not expressed by malignant cells both in the intestine and in the bone marrow by *in situ* hybridization (mRNAs EBER). Chimerism studies at this time showed 100% donor in bone marrow, but there was residual lymphoblastic leukemia disease ($V\delta_1 J\delta_1+$) present at low level both in the bone marrow and the abdominal tumor. No monoclonal B-cell proliferation was observed. As a result of his very poor clinical status, the patient was given prednisolone daily and vinblastine weekly, 3 weeks a month. After three courses of this treatment, a partial response was obtained but recurrent febrile neutropenia required chemotherapy disruption. He was then given three donor lymphocyte infusions (DLIs) (1×10^7, 1×10^7 and 1×10^8 cells/kg, respectively) from the 12th to 15th month after the transplantation without sign of GVHD reactivation. After 1 month, a bone marrow biopsy showed persistent massive infiltration by malignant histiocytes. The patient then received thalidomide daily (150 mg/day). To date, 33 months after histiocytic sarcoma diagnosis, he is still receiving thalidomide and even though the osteolytic lesions remain stable, all the other disease sites have disappeared and the child is well without evidence of neurological side effects.

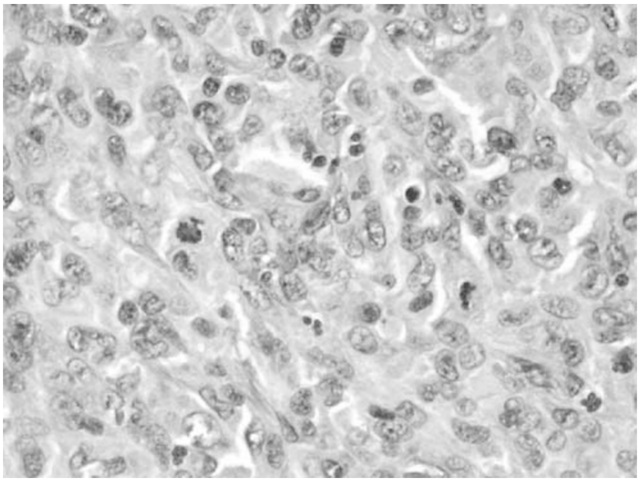

Figure 1 Two mitosis with atypia and highly irregular nuclear outlines.

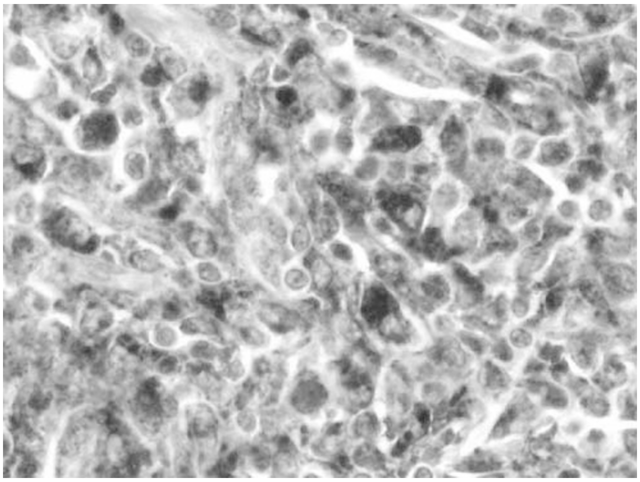

Figure 2 Positive immunohistochemical staining for CD68.

Received: 22 May 2003; accepted: 6 June 2003

Discussion

Histiocytic sarcoma is a very rare malignant proliferation and its definition remains controversial.[1] The International Lymphoma Study Group published recently a classification.[2] According to this classification, our patient presented histiocytic sarcoma defined by CD68+ and CD1a−. Owing to systemic involvement, the diagnosis was consistent with 'malignant hystiocytosis'. Interestingly, of the 18 cases of granulocytic sarcoma reported by Pileri, three had systemic presentation of the disease and two of whom previously presented acute lymphoblastic leukemia or myelodysplasia. Most histiocytic sarcomas are treated as high-grade non-Hodgkin's lymphomas but the prognosis seems very poor.[1,3] The pathogenesis of the histiocytic sarcomas is not well understood. In the case reported here, the etiologic role of the previous ALL and transplantation, or the high immunosuppression level using novel drug as MMF is unknown. The EBV genome was expressed neither by blood cells or by tumor cells. The patient presented a 100% donor chimerism in blood but the exact tumor origin, donor or recipient, remains unknown. Three other cases of histiocytic sarcoma following therapy for lymphoblastic neoplasms were published in one adult and two children. Neither had been transplanted before the diagnosis of histiocytic sarcoma.[4] Nevertheless, the histiocytic sarcoma observation is too rare to conclude about the role of previous hematological malignancy, transplantation or intensive immunosuppression on its pathogenesis.

All three patients reported by Soslow received intensive chemotherapy as histiocytic sarcoma treatment and two were alive with disease at the time of last follow-up. The very precarious clinical status of our patient at the time of histiocytic sarcoma diagnosis associated with the relative recent bone marrow transplantation (9 months) did not permit the use of aggressive chemotherapy. Even vinblastine has not been tolerated more than a few weeks. After DLI failed, we did a trial of thalidomide therapy. Although thalidomide was withdrawn in the early 1960s because of its severe teratogenic effects, it is being used again for the past few years to treat a variety of diseases such as inflammatory disease (as Crohn's disease or Behcet's syndrome) in addition to a variety of cancers, such as colon cancer and glioma and, in hematology, multiple myeloma as reported by Rajkumar *et al.*[5] In hematology, thalidomide is also used for severe, extensive and refractory cutaneous chronic GVHD that resemble sclerodermia. A recent review reported the use of thalidomide in pediatric patients and concluded that 'it should be used as a last resort when all other therapies fail'.[6] Thalidomide presents different potential mechanisms of antitumoral activity: angiogenic properties by vascular endothelial growth factor and basic fibroblast growth factor inhibition, cytokine regulation (inhibition of TNF-α production, stimulation of IL-2 production), apoptosis induction and possibly direct cytotoxicity effect by free radical-mediated oxidative DNA damage.[5,7,8] Most of thalidomide side effects such as drowsiness, weakness, dizziness, tingling, numbness or skin rash, appear dose related, and only 10–30% of patients must discontinue the treatment because of side effects. Neuropathy is observed more frequently in women and older patients. In this case, after almost 3 years of thalidomide use, the boy is well without severe side effects. Nevertheless, complete remission has not been obtained and several bone sites of disease remain stable. Different therapeutic questions remain to be discussed: how long thalidomide must be continued? What kind of positive effect is expected? Is the patient now able to receive aggressive chemotherapy in order to cure THL and if so, what kind of chemotherapy?

This case report confirms the rare but possible development of histiocytic sarcoma after a lymphoblastic neoplasm and shows the potential efficiency of thalidomide in histiocytic cell proliferation and its good tolerance in a single child.

JH Dalle[1]
P Leblond[1]
A Decouvelaere[2]
I Yakoub-Agha[3]
C Preudhomme[4]
B Nelken[1]
F Mazingue[1]

[1]*Unité Protégée A, Hôpital Jeanne de Flandre, Lille, France;*
[2]*Service d'Anatomie Pathologique, Hôpital Calmette, Lille, France;*
[3]*Unité de Greffes Médullaires, Service de Maladie du Sang, Hôpital Claude Huriez, Lille, France;*
[4]*Laboratoire d'hématologie, Hôpital Calmette, Lille, France*

References

1 Elghetany MT. True histiocytic lymphoma: is it an entity? *Leukemia* 1997; **11**: 762–764.
2 Pileri SA, Grogan TM, Harris NL, Banks P, Campo E, Chan JK *et al.* Tumours of histiocytes and accessory dendritic cells: an immunohistochemical approach to classification from the International Lymphoma Study Group based on 61 cases. *Histopathology* 2002; **41**: 1–29.
3 Ralfkiaer E, Delsol G, O'Connor NT, Brandtzaeg P, Brousset P, Vejlsgaard GL *et al.* Malignant lymphomas of true histiocytic origin. A clinical, histological, immunophenotypic and genotypic study. *J Pathol* 1990; **160**: 9–17.
4 Soslow RA, Davis RE, Warnke RA, Cleary ML, Kamel OW. True histiocytic lymphoma following therapy for lymphoblastic neoplasms. *Blood* 1996; **87**: 5207–5212.
5 Rajkumar SV, Dispenzieri A, Fonseca R, Lacy MQ, Geyer S, Lust JA *et al.* Thalidomide for previously untreated indolent or smoldering multiple myeloma. *Leukemia* 2001; **15**: 1274–1276.
6 Bessmertny O, Pham T. Thalidomide use in pediatric patients. *Ann Pharmacother* 2002; **36**: 521–525.
7 Baidas S, Tfayli A, Bhargava P. Thalidomide: an old drug with new clinical applications. *Cancer Invest* 2002; **20**: 835–848.
8 Gupta D, Treon SP, Shima Y, Hideshima T, Podar K, Tai YT *et al.* Adherence of multiple myeloma cells to bone marrow stromal cells upregulates vascular endothelial growth factor secretion: therapeutic applications. *Leukemia* 2001; **15**: 1950–1961.

Hairy cell leukemia and sarcoidosis: a case report and review of the literature

Leukemia (2003) **17,** 2057–2059. doi:10.1038/sj.leu.2403074

TO THE EDITOR

Hairy cell leukemia (HCL) is a hematologic neoplasm characterized by infiltration of the bone marrow and spleen by abnormal lymphocytes with prominent hair-like projections. The disorder is rare, constituting roughly 2% of adult leukemias with 600–800 diagnoses per year in the United States. The median age at the time of diagnosis is 52 years with a male to female ratio of 4:1.[1] Given current therapy with purine nucleoside analogues, the prognosis of HCL is favorable, with progression-free survival estimated to be 70% at 4 years.[2]

Secondary malignancies have frequently been described in HCL, with an estimated two-fold risk for development of solid tumors among patients treated with purine analogues.[3] A number of nonmalignant disorders have also been associated

Received: 25 March 2003; accepted: 28 May 2003
