# Supplementary material for: An 8-year-old girl with secondary histiocytic sarcoma with BRAFV600 mutation following T-cell acute lymphoblastic leukemia demonstrating stable disease for 3 years on dabrafenib and trametinib – a case report and literature review
Source: BMC Pediatr. 2025 Mar 8;25:178. doi: 10.1186/s12887-025-05539-2 (PMC11889787; doi:10.1186/s12887-025-05539-2)
Supplement: Supplementary file 9 — Supplementary Material 9 [file 12887_2025_5539_MOESM9_ESM.pdf]

Received: 21 October 2019 | Revised: 2 January 2020 | Accepted: 20 January 2020
DOI: 10.1002/pbc.28200

Pediatric Blood & Cancer
SOCIÉTÉ INTERNATIONALE D'ONCOLOGIE PÉDIATRIQUE
INTERNATIONAL SOCIETY OF PAEDIATRIC ONCOLOGY
aspho The American Society of Pediatric Hematology/Oncology
WILEY

Check for updates

LETTER TO THE EDITOR

Secondary histiocytic sarcoma with BRAF^{V600E} mutation after T-cell acute lymphoblastic leukemia in a very young child with dramatic response to dabrafenib and trametinib

To the Editor: We report an unusual case of a child with infant T-cell acute lymphoblastic leukemia (T-ALL) who presented during maintenance chemotherapy with *BRAF*^{V600E}-mutant histiocytic sarcoma (HS) that responded to mitogen-activated protein kinase (MAPK)-targeted therapy with dabrafenib and trametinib.

A male infant presented at 5 months of age with fevers, hepatosplenomegaly, and white blood cell count over 600 000/mm^3. Bone marrow aspiration (BMA) demonstrated 91% lymphoblasts with an immature T-cell immunophenotype. Cytogenetic studies identified t(8;14)(q24;q11.2), resulting in a *TRA-MYC* rearrangement. Cerebrospinal fluid (CSF) contained 70 white blood cells per microliter with 90% blasts.

He received induction chemotherapy following a Children's Oncology Group (COG) infant ALL trial, AALL15P1. Postinduction BMA showed complete remission with flow cytometry negative for minimal residual disease (<0.01%) and clearance of CSF leukemia. Postinduction therapy employed a Berlin-Frankfurt-Munster chemotherapy backbone following the intermediate risk arm of COG T-ALL trial AALL1231.

Four months after starting maintenance therapy, the patient presented with a rapidly growing temporal mass (Figure 1A, parental consent obtained). Computed tomography (CT) and magnetic resonance imaging (MRI) showed a 3.6 cm right temporal skull mass with intracranial and extracranial components. BMA showed no evidence of T-ALL; ALL therapy was discontinued during diagnostic evaluation.

Biopsy of the mass revealed morphologic features of HS with immunohistochemical stains positive for CD68, CD163, PU.1, CD45, CD4, MYC, S100 (faint), and BRAF^{V600E} (VE1 antibody) (Figure 2A-C). Negative staining was demonstrated for CD3, CD34, CD117, TdT, as well as specific markers of melanocytic, Langerhans cell, and follicular dendritic cell lineage. Fluorescence in situ hybridization (FISH) testing identified rearrangement of *MYC*, indicating a clonal relationship with the patient's prior T-ALL (Figure 2D). Additionally, *CDKN2A* copy loss and *BRAF p.V600E* were identified by targeted sequencing.

The tumor expanded during 5 days of clofarabine and dexamethasone. Dabrafenib (BRAF inhibitor) and trametinib (MEK1/2 inhibitor) were obtained in liquid formulations through a compassionate use, IRB-approved protocol—dabrafenib twice daily at 2.625 mg/kg, and trametinib once daily at 0.025 mg/kg. Significant response was observed over the first few days (Figure 1B-C). The trametinib dose was increased 7 weeks later to 0.032 mg/kg when the tumor showed some regrowth. Restaging scans showed significant improvement but persistent skull-based tumor, prompting complete surgical resection with cranioplasty that achieved negative margins. The posttreatment excision showed residual tumor with weak BRAF^{V600E} expression by immunohistochemistry. Dabrafenib/trametinib was continued until 1 year after resection. ALL therapy was never resumed given lack of data regarding combining therapies. He remains at risk for relapse; however, there is no evidence of disease 14 months after HS diagnosis and 28 months after T-ALL diagnosis.

This is the first report of a child with infant T-ALL developing secondary *BRAF*^{V600E}-mutant HS responding to MAPK-targeted therapy. Five reported cases of secondary HS after ALL show patients in the age group of 4-10 years, all presented during maintenance chemotherapy, sharing molecular markers of the initial ALL,[1-5] two with *CDKN2A* mutations[2,5] and none with *BRAF*^{V600E} mutation.

In HS, broadly, activating genomic alterations of the MAPK pathway are present in the majority of cases and tumor suppressor *CDKN2A* copy number loss is frequent.[6-8] Medications that target the MAPK pathway, including BRAF and MEK, have demonstrated activity in *BRAF*^{V600E}-mutant solid tumors, such as melanoma,[9-13] anaplastic thyroid cancer,[14] non-small cell lung carcinoma,[15] colorectal cancer,[16] and Erdheim-Chester disease.[17] Two reports show response to vemurafenib in *BRAF*^{V600E}-mutant HS.[18,19] Additionally, combined BRAF/MEK inhibition has been shown to be more effective than single-agent BRAF inhibitors in four randomized phase III trials in metastatic melanoma.[9-12]

In summary, we treated a very young child with aggressive secondary *BRAF*^{V600E}-mutant HS with dabrafenib and trametinib; he remains in remission 14 months later, demonstrating a therapeutic benefit of MAPK-targeted therapy in *BRAF*-mutant HS.

CONFLICT OF INTEREST

Ryan J. Sullivan has worked as *advisory boards/consultant* for Array Biopharma, Merck, and Novartis; received *research support* from Merck; and *clinical trials support* from Roche-Genentech, Novartis, Merck, Array Biopharma, BioMed Valley Discoveries, Asana, Lilly, and Astex. All other authors have no conflict of interest.

© 2020 Wiley Periodicals, Inc.

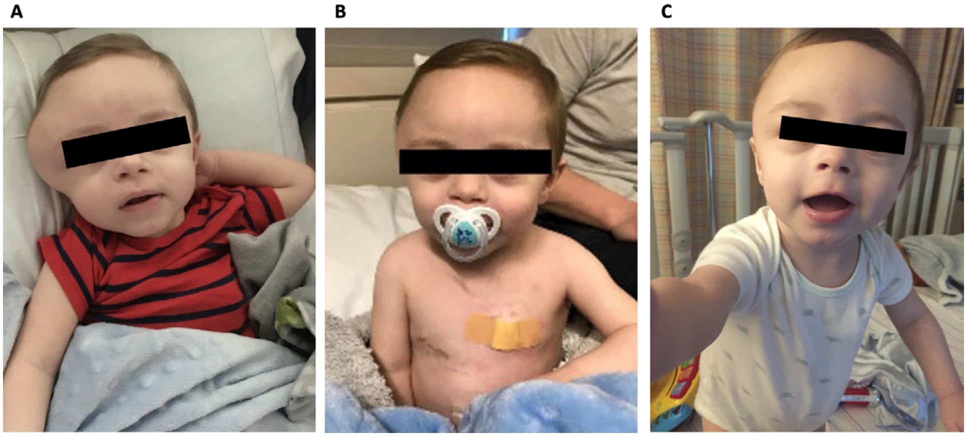

FIGURE 1 Right temporal skull mass prior to and after starting dabrafenib/trametinib treatment. (A) The day dabrafenib/trametinib treatment was started; (B) three days after starting dabrafenib/trametinib; (C) six days after starting dabrafenib/trametinib treatment. His family provided consent to publish images

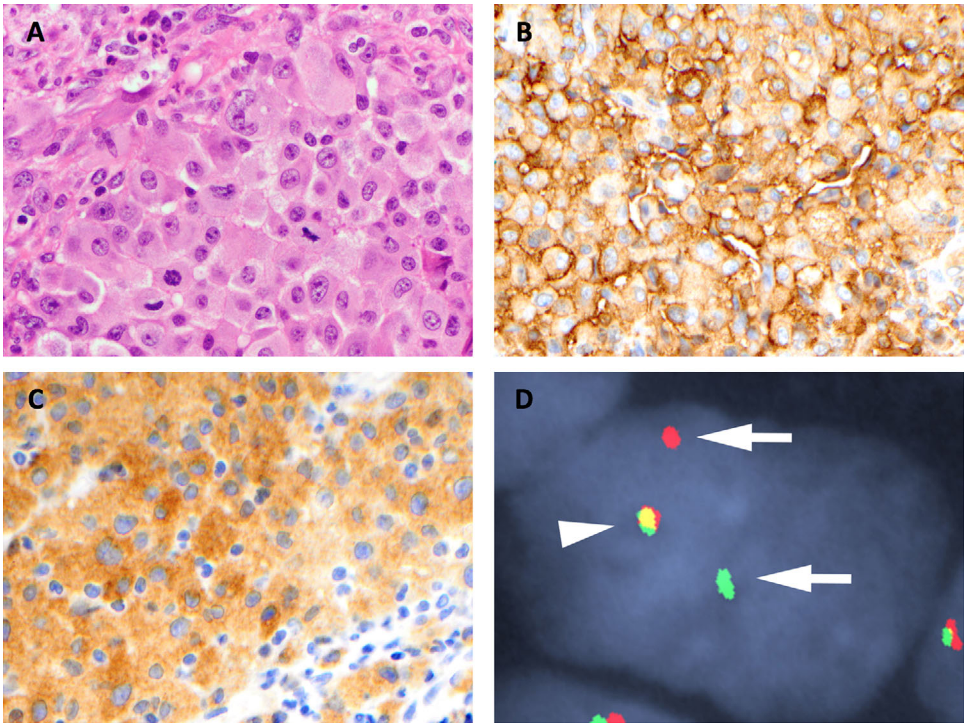

FIGURE 2 Biopsy of skull mass and *MYC* break-apart FISH assay. A hematoxylin and eosin stain of the skull mass demonstrates sheets of large, pleomorphic cells with rounded to lobulated nuclei, distinct nucleoli, and abundant, eosinophilic cytoplasm; the overall appearance of the tumor cells is reminiscent of tissue histiocytes (A). Immunohistochemistry for CD163 stains tumor cytoplasm and cell membranes diffusely (B), while $BRAF^{V600E}$ shows strong cytoplasmic staining (C). MYC break-apart FISH assay demonstrates split red and green signals (arrows), indicating rearrangement of MYC, and one normal fused probe (arrowhead) (D)

ORCID

Vinayak Venkataraman https://orcid.org/0000-0003-0941-2354

Vinayak Venkataraman[1]
Lucas R. Massoth[2]
Ryan J. Sullivan[3]
Alison M. Friedmann[4]

[1] *Departments of Medicine and Pediatrics, Massachusetts General Hospital, Boston, Massachusetts*
[2] *Department of Pathology, Massachusetts General Hospital, Boston, Massachusetts*
[3] *Department of Medicine, Massachusetts General Hospital, Boston, Massachusetts*
[4] *Department of Pediatrics, Massachusetts General Hospital, Boston, Massachusetts*

Correspondence
Alison M. Friedmann, Department of Pediatrics, Massachusetts General Hospital, 55 Fruit Street, Boston, MA 02114.
Email: afriedmann@partners.org

REFERENCES

1. Ansari J, Nagash AR, Munker R, et al. Histiocytic sarcoma as a secondary malignancy: pathobiology, diagnosis, and treatment. *Eur J Haematol*. 2016;97(1):9-16.
2. Alten J, Klapper W, Leuschner I, et al. Secondary histiocytic sarcoma may cause apparent persistence or recurrence of minimal residual disease in childhood acute lymphoblastic leukemia. *Pediatr Blood Cancer*. 2015;62(9):1656-1660.
3. Dalle JH, Leblond P, Decouvelaere A, et al. Efficacy of thalidomide in a child with histiocytic sarcoma following allogenic bone marrow transplant for T-ALL. *Leukemia*. 2003;17(10):2056-2057.
4. Ganapule AP, Gupta M, Kokil G, et al. Histiocytic sarcoma with acute lymphoblastic leukemia a rare association: case report and literature review. *Indian J Hematol Blood Transfus*. 2014;30(Suppl 1):305-308.
5. Kumar R, Khan SP, Joshi DD, et al. Pediatric histiocytic sarcoma clonally related to precursor B-cell acute lymphoblastic leukemia with homozygous deletion of CDKN2A encoding p16INK4A. *Pediatr Blood Cancer*. 2011;56(2):307-310.
6. Shanmugam V, Griffin GK, Jacobsen ED, et al. Identification of diverse activating mutations of the RAS-MAPK pathway in histiocytic sarcoma. *Mod Pathol*. 2019;32(6):830-843.
7. Egan C, Nicolae A, Lack J, et al. Genomic profiling of primary histiocytic sarcoma reveals two molecular subgroups. *Hematologica*. 2019. https://doi.org/10.3324/haematol.2019.230375
8. Massoth LR, Hung YP, Ferry JA, et al. Comprehensive genomic profiling of 104 rare histiocytic and dendritic cell neoplasms reveals shared and distinct targetable genomic alterations. *Blood*. 2019;134(Suppl. 1):2541.
9. Robert C, Karaszewska B, Schachter J, et al. Improved overall survival in melanoma with combined dabrafenib and trametinib. *N Engl J Med*. 2015;372:30-39.
10. Long GV, Stroyakovskiy D, Gogas H, et al. Combined BRAF and MEK inhibition versus BRAF inhibition alone in melanoma. *N Engl J Med*. 2014;371:1877-1888.
11. Larkin J, Ascierto PA, Dreno B, et al. Combined vemurafenib and cobimetinib in BRAF-mutated melanoma. *N Engl J Med*. 2014;371(30):1867-1876.
12. Dummer R, Ascierto PA, Gogas HJ, et al. Overall survival in patients with BRAF-mutant melanoma receiving encorafenib plus binimetinib versus vemurafenib or encorafenib (COLUMBUS): a multicentre, open-label, randomized, phase 3 trial. *Lancet Oncol*. 2018;19(10):1315-1327.
13. Long GV, Hauschild A, Santinami M, et al. Adjuvant dabrafenib plus trametinib in stage III BRAF-mutated melanoma. *N Engl J Med*. 2017;377(19):1813-1823.
14. Subbiah V, Kreitman RJ, Wainberg ZA, et al. Dabrafenib and trametinib treatment in patients with locally advanced or metastatic BRAF V600-mutant anaplastic thyroid cancer. *J Clin Oncol*. 2018;36(1):7-13.
15. Planchard D, Smit EF, Groen HJM, et al. Dabrafenib plus trametinib in patients with previously untreated $BRAF^{V600E}$-mutant metastatic non-small-cell lung cancer: an open-label, phase 2 trial. *Lancet Oncol*. 2017;18(10):1307-1316.
16. Kopetz S, Grothey A, Yaeger R, et al. Encorafenib, binimetinib, and cetuximab in *BRAF* V600E-mutated colorectal cancer. *N Engl J Med*. 2019;381:1632-1643.
17. Idbaih A, Mokhtari K, Emile JF, et al. Dramatic response of a BRAF V600E-mutated primary CNS histiocytic sarcoma to vemurafenib. *Neurology*. 2014;83(16):1478-1480.
18. Branco B, Comont T, Ysebaert L, et al. Targeted therapy of BRAF V600E-mutant histiocytic sarcoma: a case report and review of the literature. *Eur J Haematol*. 2019;103(4):444-448.
19. Diamond EL, Subbiah V, Lockhart AC, et al. Vemurafenib for BRAF V600-mutant Erdheim-Chester disease and Langerhans cell histiocytosis: analysis of data from the histology-independent, phase 2, open-label VE-BASKET study. 2018;4:384-388.
