# Supplementary material for: An 8-year-old girl with secondary histiocytic sarcoma with BRAFV600 mutation following T-cell acute lymphoblastic leukemia demonstrating stable disease for 3 years on dabrafenib and trametinib – a case report and literature review
Source: BMC Pediatr. 2025 Mar 8;25:178. doi: 10.1186/s12887-025-05539-2 (PMC11889787; doi:10.1186/s12887-025-05539-2)
Supplement: Supplementary file 10 — Supplementary Material 10 [file 12887_2025_5539_MOESM10_ESM.pdf]

Check for updates

True Histiocytic Lymphoma Following Therapy for Lymphoblastic Neoplasms

By Robert A. Soslow, R. Eric Davis, Roger A. Warnke, Michael L. Cleary, and Onsi W. Kamel

True histiocytic lymphomas (THLs) are rare tumors in which the malignant cells show morphologic and immunophenotypic evidence of histiocytic differentiation. We describe THLs that arose after therapy for one case of T-lineage lymphoblastic lymphoma (LyL) and two cases of acute lymphoblastic leukemia (ALL) (both CD10$^+$, one pre-B phenotype). The lymphoblastic neoplasms were not unusual in any way, and responded well to standard therapy. The THLs arose 10 to 20 months after complete remission was achieved for the lymphoblastic neoplasms, at which time there was still no clinical or pathologic evidence of the lymphoblastic neoplasms. All three THLs exhibited clinical and morphologic features of malignancy. Neoplastic cells in the THLs had abundant eosinophilic vacuolated cytoplasm and pleomorphic nuclei, and expressed histiocytic antigens in the absence of lymphocyte-specific lineage markers. Because THLs are rare neoplasms, their occurrence after otherwise successful therapy for lymphoblastic neoplasms in these three cases may constitute a distinct clinicopathologic entity.

© ***1996 by The American Society of Hematology.***

THE CONCEPT OF A true histiocytic malignancy distinct from other well-characterized histiocytic disorders has been controversial. Recently, several investigators have defined the morphologic and immunophenotypic features of true histiocytic lymphoma (THL) and its distinction from previously described proliferations such as so-called malignant histiocytosis.[1-7] Other studies have also more fully characterized entities with which THL could be confused, such as anaplastic large-cell lymphoma (ALCL), angiocentric immunoproliferative disorders, and various hemophagocytic syndromes.[4,8-10]

When current definitional criteria are applied, THLs are rare tumors. Most investigators require THLs to show morphologic features consistent with histiocytic differentiation and express histiocytic markers, in the absence of B-cell or T-cell lineage-specific immunologic markers.[1-8] THLs must display cytologic features of malignancy, and be distinguishable on morphologic grounds from reactive histiocytic proliferations (such as those occurring in response to viral infections). Within the spectrum of THLs, some investigators include neoplasms that display clonal immunoglobulin or T-cell receptor gene rearrangements, provided that the neoplasms are morphologically and phenotypically histiocytic, because in this setting these gene rearrangements do not necessarily indicate the corresponding lineage.[2,5,11,12]

A variety of histiocytic disorders have been described in the setting of lymphoblastic lymphoma (LyL) or acute lymphoblastic leukemia (ALL),[13-26] but most of these reports were published before the application of current immunophenotyping and genotyping techniques, and many preceded the description of entities such as ALCL and virus-associated hemophagocytic syndromes. At least some of these cases appear to represent examples of hemophagocytic syndromes, associated with viral infection or ALCL.[13,15,26] We report three cases of LyL or ALL, successfully treated with chemotherapy and/or radiation therapy, in which second neoplasms arose and displayed the morphologic and immunophenotypic features of THLs.

CASE HISTORIES

Case no. 1. This 27-year-old man presented with a mediastinal mass, on which a biopsy was performed and a diagnosis of LyL given. Immunophenotypic studies were indicative of a T-lineage neoplasm. Bilateral bone marrow biopsy specimens showed no evidence of lymphoma. He received chemotherapy followed by bone marrow transplantation, and achieved a complete remission. Fifteen months later, he developed a recurrent mediastinal mass, on which a biopsy was performed and a diagnosis of a malignant neoplasm with morphologic and immunophenotypic features of THL given. He was treated with etoposide (VP-16), cisplatin, and radiation therapy, but has had only a partial response; he was alive with disease at the time of his last follow-up evaluation, 1 year after the diagnosis of THL.

Case no. 2. This 8-year-old boy presented with leukocytosis of 56,300/μL, 88% of which was due to blasts. Immunophenotyping of the blasts by flow cytometry and slide-based methods (Table 1) showed a pre-B phenotypic stage of ALL. He was treated according to the UKALL X protocol (high-dose methotrexate with prophylactic cranial irradiation) and obtained a complete remission. Ten months later, he developed a paraspinous mass that destroyed the L1 vertebral body; a biopsy was performed and a diagnosis of THL given. He was treated with VP-16 and methylprednisolone, but died of disease 3 months after presenting with THL. Postmortem examination demonstrated widely infiltrative THL involving bone, lung, liver, and spleen. There was no histologic evidence of residual ALL.

Case no. 3. This 6-year-old boy developed CD10$^+$ ALL. Additional details regarding immunophenotyping studies were not available. He was treated with chemotherapy according to the BFM 86 protocol (prednisone, asparaginase, vincristine, cytarabine, mercaptopurine, methotrexate, cyclophosphamide, doxorubicin, and dexamethasone) and achieved a complete remission. He developed an osteolytic scapular mass 20 months later, on which a biopsy was performed and a diagnosis of THL given; he was treated with ifosfamide, VP-16, and carboplatin. He remains alive 16 months later but has developed new lesions involving the paravertebral tissues, lung, and liver.

MATERIALS AND METHODS

Case selection and morphologic review. All cases were submitted in consultation to the Laboratory of Surgical Pathology at Stan-

From the Department of Pathology, Stanford University Medical Center, Stanford, CA.

Submitted July 7, 1995; accepted February 6, 1996.

Supported in part by Grants No. 34233 and 33119 from the National Cancer Institute and by a Stanford University Research Incentive Fund Award.

Address reprint requests to Onsi W. Kamel, MD, Department of Pathology, Stanford University Medical Center, 300 Pasteur Dr, Stanford, CA 94305.

The publication costs of this article were defrayed in part by page charge payment. This article must therefore be hereby marked "advertisement" *in accordance with 18 U.S.C. section 1734 solely to indicate this fact.*

© 1996 by The American Society of Hematology.

0006-4971/96/8712-0012$3.00/0

Downloaded from http://ashpublications.org/blood/article-pdf/87/12/5207/620054/5207.pdf by guest on 28 September 2023

Table 1. Immunophenotypic Results in Original Lymphoblastic Neoplasms and Subsequent THLS

Case no. 1	
LyL	
Positive: CD 3, 43, 45RO, and 45; TdT	
Negative: CD 20, 34, and 68; myeloperoxidase	
THL	
Positive: CD 4, 11c, 13, 14, and 68	
Negative: CD 1a, 3, 5, 7, 8, 19, and 30	
Case no. 2	
ALL	
Positive: CD 10, 19, 22 (surface), and 34; IgM (cytoplasmic); TdT; HLA-DR	
Negative: CD 1, 2, 3, 4, 5, 7, 8, 13, and 33; T-cell receptor beta and delta chains	
THL	
Positive: CD 45 and 68; S100 protein (in scattered cells)	
Negative: CD 15, 20, 30, 43, 45RA, and 45RO; lysozyme	
Case no. 3	
ALL	
Positive: CD10	
Negative: not specified	
THL	
Positive: CD68, S100 protein (focal staining)	
Negative: CD 3, 15, 20, 30, 34, 45RA, and 45RO; myeloperoxidase	

Abbreviation: TdT, terminal deoxynucleotidyl transferase.

ford University Medical Center between 1990 and 1994. The cases were submitted because of the unusual nature of the second neoplasms. The three patients had LyL or ALL that had been well characterized morphologically and immunophenotypically, and these diagnoses were confirmed on review of the original pretreatment slides. Diagnosis of the second neoplasms as THLs was based on the following criteria: (1) histologic features consistent with histiocytic differentiation, (2) sufficiently atypical cytologic features to be considered neoplastic (ie, a reactive histiocytic proliferation was excluded on morphologic grounds), and (3) an immunophenotype supporting histiocytic differentiation (ie, reactivity with one or more histiocyte-associated markers, and no reactivity with B-cell– or T-cell–restricted antibodies).[6]

Immunohistochemistry. Paraffin sections in all histiocytic neoplasms and of the original lymphoblastic neoplasm in case no. 1 were immunostained using a biotin-streptavidin method.[27] Details of the method and specific antibodies used including sources have been previously published.[7] In paraffin sections, antibodies against some or all of the following antigens were used depending on tissue availability: CDs 3, 15, 20, 30, 34, 43, 45RB, 45RA, 45RO, and 68; myeloperoxidase; lysozyme; and S100. The THL in case no. 1 was also immunophenotyped in frozen sections (the only case in which frozen tissue was available) using antibodies to the following CD antigens: 1a, 2, 3, 4, 5, 7, 8, 10, 11c, 13, 14, 19, 20, and 68.

Gene rearrangement studies. These studies were performed (case no. 1) as previously described.[28] Briefly, DNA was extracted from fresh-frozen tissue, purified, and digested with appropriate restriction enzymes (New England BioLabs, Beverly, MA). The resulting fragments were separated by electrophoresis in a 0.8% agarose gel and transferred to nylon membranes. The membranes were hybridized with ^{32}P-radiolabeled DNA fragments, washed, and subjected to autoradiography.[28] DNA probes used were fragments of the constant and joining regions of the T-cell receptor beta-chain (TCR-beta) gene, the joining region of the immunoglobulin heavy-chain gene, and the constant regions of the kappa and lambda light-chain genes.

In situ hybridization studies for Epstein-Barr virus. Epstein-Barr virus (EBV) RNA was detected using a 30-base oligonucleotide probe complimentary to a portion of the EBER-1 gene, as previously described.[29] Five-micron thick paraffin sections were deparaffinized, rehydrated, predigested with proteinase K, and hybridized overnight at a concentration of 0.25 ng/μL of biotinylated probe. Detection was accomplished using an avidin-alkaline phosphatase conjugate. For each assay, a known EBV-positive neoplasm and EBV-negative lymphoid tissue served as positive and negative controls, respectively. A poly-dT probe was used to demonstrate the integrity of the mRNA in each tissue sample.

RESULTS

Morphology. Review of the morphology and immunophenotyping results in the original tumors confirmed the diagnoses of LyL in case no. 1 and ALL in cases no. 2 and 3. The mediastinal biopsy from patient no. 1 showed a dense infiltrate of lymphoblastic cells with convoluted nuclei, evenly dispersed chromatin, and scant amounts of cytoplasm (Fig 1A). Because there was no evidence of involvement of blood or bone marrow, this was diagnosed as a LyL, rather than ALL. In cases no. 2 and 3, the peripheral blood contained uniform medium-sized blasts with scant cytoplasm and occasional small inconspicuous nucleoli, consistent with French-American-British (FAB) type L1. Because of extensive involvement of blood and bone marrow, patients no. 2 and 3 were diagnosed with ALL.

The three THLs were morphologically distinct from the original lymphoblastic neoplasms. The THLs were composed of large pale-staining cells that were loosely cohesive to noncohesive, and contained abundant vacuolated pink cytoplasm. The nuclei ranged from round to oval to bean-shaped and demonstrated a vesicular chromatin pattern. Multinucleated tumor giant cells were present in each case, as were cytologic features of pleomorphism or frank anaplasia (Fig 1B). In some areas in case no. 3, the constituent cells showed a more uniform appearance, with nuclei containing grooves or folds suggestive of Langerhans cell differentiation (Fig 2); however, the other areas of the tumor showed morphologic features (described earlier) that precluded this diagnosis. Mitotic figures were a consistent finding. Hemophagocytosis was present, but was not prominent. Other morphologic features included areas of tumor-cell necrosis and focal collections of neutrophils. There was no evidence of residual lymphoblastic neoplasia in any of the three cases.

Immunophenotype. As summarized in Table 1, the THLs stained with one or more histiocytic markers, including CD11c, CD13, CD14, or CD68 (Fig 2B). Individual cases also demonstrated immunoreactivity with CD45, cytoplasmic CD4, and S100 (scattered positive cells in case no. 2 and focal S100 staining in case no. 3), markers that may be seen in cells of histiocytic lineage. In contrast, tumor cells showed no reactivity with markers specific for B or T lineages, or for CD30.

In situ hybridization for EBV. All three cases were negative for EBV EBER-1 RNA. The integrity of mRNA in the

Downloaded from http://ashpublications.org/blood/article-pdf/87/12/5207/620054/5207.pdf by guest on 28 September 2023

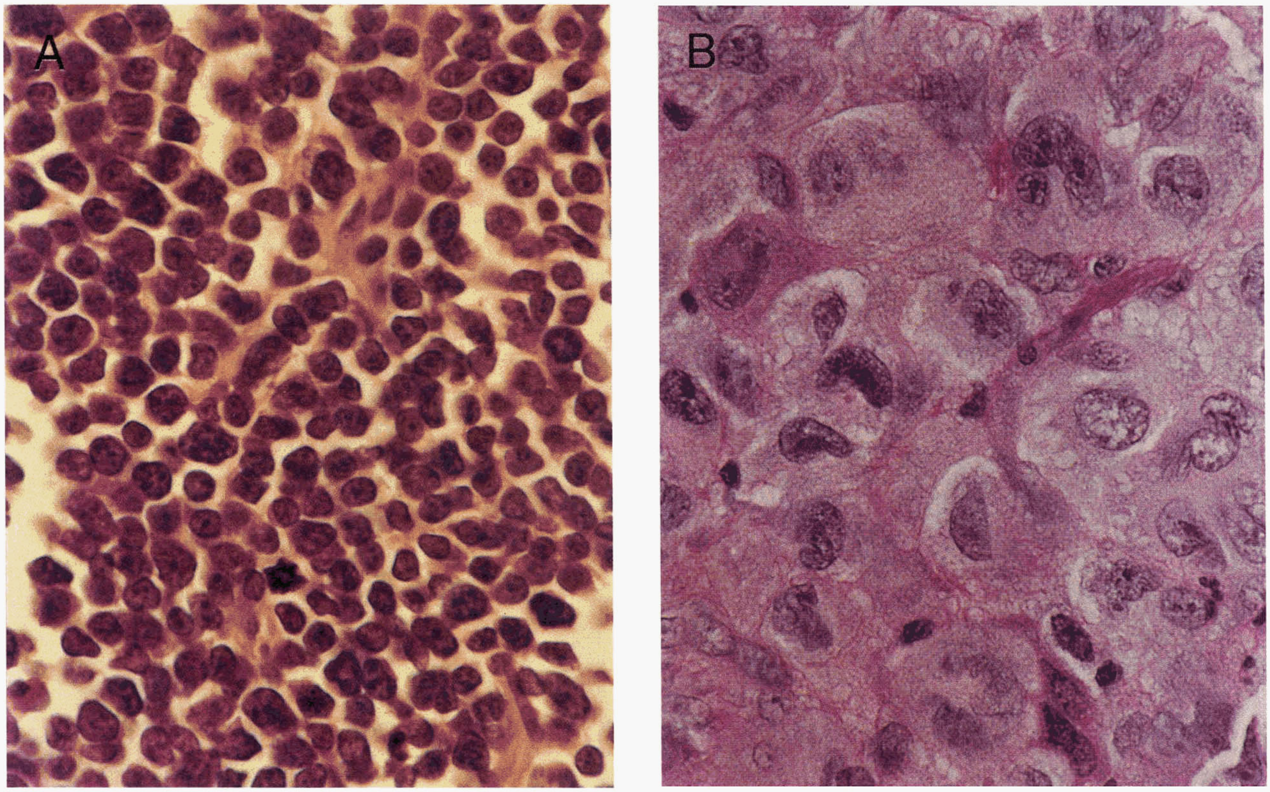

Fig 1. The LyL (A, hematoxylin and eosin) and the THL (B, hematoxilin and eosin) that developed 15 months following a successful bone marrow transplantation in patient no. 1. The THL showed a clonal rearrangement of the TCR-beta gene. Original magnifications × 600.

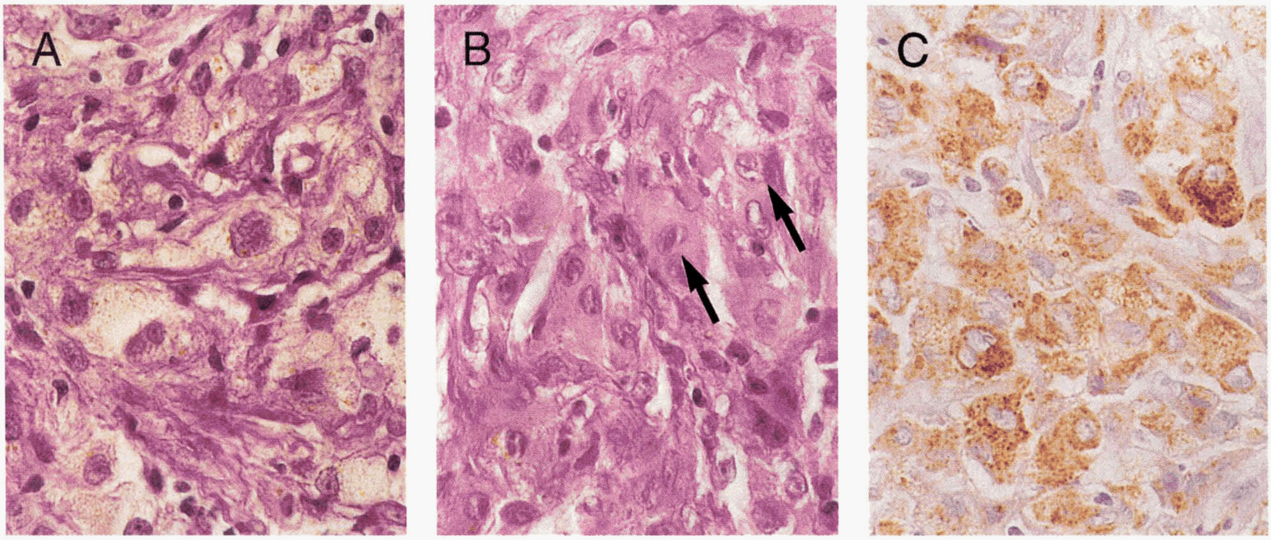

Fig 2. The THL in patient no. 3 showed cells with nuclear pleomorphism (A, hematoxylin and eosin; original magnification × 400) and also showed some areas containing cells with grooved or folded nuclei (arrows) suggestive of Langerhans cell differentiation (B, hematoxylin and eosin; original magnification × 400). Reactivity with CD68, a histiocyte-associated marker, is demonstrated in the neoplastic cells (B, KP1 [anti-CD68]).

Downloaded from http://ashpublications.org/blood/article-pdf/87/12/5207/620054/5207.pdf by guest on 28 September 2023

tissue samples was established by reaction with a poly-dT probe.

Gene rearrangement studies. A clonal rearrangement of the T-cell receptor beta-chain gene was found in the THL of patient no. 1; there was no rearrangement of the immunoglobulin heavy-chain gene or light-chain genes.

DISCUSSION

We report three cases of THL that arose after therapy for LyL or ALL. These tumors expressed histiocytic markers and lacked B-cell and T-cell lineage-specific markers, evidence against B- or T-lineage lymphoma. During the years 1975 to 1981, several investigators reported histiocytic neoplasms in association with ALL[17-19,21-25]; however, these were not studied by immunophenotyping and appear to represent a variety of entities containing histiocytes (discussed further later).

It has been observed for many years that cells with the morphologic appearance of histiocytes are a prominent component in some hematolymphoid malignancies, and, in many instances, these cells appear to be engaged in phagocytosis. However, it has not always been clear whether these cells are, in fact, of histiocytic lineage, or whether they are truly neoplastic (as opposed to proliferating in response to other neoplastic cells or to a viral infection). Nonetheless, before the application of immunophenotyping, there were reports of histiocytic neoplasms; these reports used a variety of names, most commonly malignant histiocytosis.[30-35] The application of immunohistochemical staining has shown that many cases meeting the older clinical and morphologic definition of malignant histiocytosis are actually non-Hodgkin's lymphomas of B, T, or null lineage, in which histiocytes are a prominent, but reactive component. In such cases, immunostains for markers of lymphoid lineages highlight a minor population of cells with malignant cytologic features, and these cells may also show phenotypic aberrancies such as expression of CD15, CD30, or epithelial membrane antigen[8]; histiocytes in the tumor, while numerous and sometimes hemophagocytic, do not stain for these markers and have benign or reactive cytologic features. When immunostaining is applied, some older cases of malignant histiocytosis may also correspond to other newer categories of non-Hodgkin's lymphomas with prominent reactive histiocytes, such as histiocyte-rich B-cell lymphoma.[36] Still other cases of malignant histiocytosis may be diagnosed as nonneoplastic conditions in which histiocytes are prominent, most notably, hemophagocytic syndromes.

Without the benefit of immunophenotyping, therefore, it is difficult to determine the nature of the histiocytic neoplasms in the earlier reports of histiocytic neoplasms in association with ALL. Each of these reports describes prominent erythrophagocytosis in atypical histiocytes, a phenomenon that is more prominent in reactive histiocytes than in malignant histiocytes.[2,10,37] At least some of these reports describe and illustrate cytologic features of malignancy, but it is not clear whether these represent a minority neoplastic cell population associated with reactive histiocytes.[19,23-25] Other studies do not describe or illustrate cytologic features indicative of malignancy, raising the possibility of a histiocytic proliferation reactive to a viral infection.[16,18,21,22]

In making the diagnosis of THL, it is important to exclude virus-associated histiocytic proliferations. EBV has been strongly associated with hemophagocytic histiocytic proliferations[38]; in situ staining detected EBV EBER-1 RNA in one reported case,[39] and EBV-associated hemophagocytic syndromes have been reported following chemotherapy for ALL.[40] The histologic features in our cases were not those of a virus-associated hemophagocytic syndrome, and none of our cases contained EBV EBER-1 RNA by in situ hybridization studies. There was no other known evidence of viral infection in our three cases, and tissue culture and serology from patient no. 2 were negative for herpes simplex virus and cytomegalovirus (CMV). Furthermore, hemophagocytic histiocytic proliferations (and many cases of ALCL, previously diagnosed as malignant histiocytosis) usually exhibit fulminant systemic clinical phenomena, including fever and profound cytopenia; these were absent in our three cases of THL. However, there is precedent in the literature for a relationship between ALL and histiocytic proliferations, both reactive hemophagocytic syndromes and Langerhans cell histiocytosis.[13,15,26,41] McClain et al reviewed the occurrence of virus-associated histiocytic proliferations in children.[42] Yin et al reported a case of a viral histiocytic medullary reticulosis-like syndrome caused by parainfluenza virus[13]; their patient received supportive care and recovered completely. However, some cases of histiocytic proliferations occurring after therapy for ALL have been lethal, whether idiopathic[26] or due to known viruses such as CMV.[15]

The definition of true histiocytic neoplasms has changed over the past 20 years, mostly due to advances in immunophenotyping.[43] A number of earlier studies examined various histiocytic markers in normal and malignant cells of presumed histiocytic lineage, with mixed results, particularly regarding the nature of malignant histiocytosis.[44-48] However, there is currently a general consensus that THLs should express at least some of the enzymes or surface antigens that have been found to be specific for histiocytes, and lack reactivity for specific markers of other lineages; with this restricted definition, THLs have been reported but are rare tumors, distinct from other large-cell lymphomas.[1,7,49] Only a limited number of markers of histiocytic differentiation are applicable to paraffin sections, and these are not entirely specific; the KP1 antibody to CD68,[50] the chief marker that we used for this purpose, stains a small proportion of B-lineage large-cell lymphomas.[51] CD68 was expressed by all of our THLs, but their failure to express B- or T-lineage markers, along with the morphologic features, was equally compelling evidence of histiocytic lineage. Their failure to express CD30 also argues against a diagnosis of ALCL, in keeping with their morphology, although CD30 is expressed by a subset of histiocytic neoplasms.[49,51,52]

At least some investigators suggest that the presence of a clonal TCR-beta gene rearrangement, which we found in the THL of case no. 1, should exclude the diagnosis of a true histiocytic neoplasm.[1,3] However, while TCR-beta and immunoglobulin gene rearrangements usually demonstrate lin-

Downloaded from http://ashpublications.org/blood/article-pdf/87/12/5207/620054/5207.pdf by guest on 28 September 2023

eage and clonality of T-cell and B-cell neoplasms, respectively, it has been well demonstrated that gene rearrangements are not always lineage-specific.[11,12,53-55] In particular, there are several reports and series of histiocytic neoplasms in which immunoglobulin and/or TCR-beta genes have been clonally rearranged.[2,5,49,56,57]

Most cases of THL have arisen without another prior neoplasm or therapy, and to our knowledge the report of van der Kwast et al is the only one which documents fully a true histiocytic phenotype in a malignant proliferation associated with ALL.[14] In that report, the investigators described the occurrence of a true histiocytic neoplasm with one rearranged IgH gene and a t(10;11) in the setting of a T-lymphoblastic lymphoma (not genotyped at original diagnosis); they concluded that the histiocytic neoplasm was present at the time of initial presentation with T-LyL, and that the two neoplasms were morphologically and immunophenotypically distinct.

The second neoplasms in our three cases were all clinically and morphologically malignant, and displayed convincing morphologic and immunophenotypic evidence of histiocytic differentiation. The question arises whether these THLs were related to the original lymphoblastic neoplasms in these patients, or were unrelated neoplasms that arose secondary to the therapy for lymphoblastic disease. Because THLs are rare, their secondary occurrence in three patients with lymphoblastic neoplasms seems greater than would be predicted by chance alone; however, this remains an exceedingly rare phenomenon, and its true incidence cannot be estimated from our practice, since these cases were referred in consultation because of the secondary neoplasms. If there is a causal relationship between the primary lymphoblastic neoplasms and the THLs, one possibility may be the therapy for the LyL or ALL, since secondary hematolymphoid neoplasms are a known complication of many antineoplastic regimens. However, these three patients received different regimens for their LyL or ALL, none of which included VP-16, which has a high rate of inducing acute myeloid leukemia.[58] In addition, with the possible exception of VP-16, therapy-related neoplasms usually require a longer interval for their development than the 10- to 20-month span in these cases. Another possible mechanism of causation is that the second neoplasms arose in a posttreatment state of immunodeficiency, as occurs in several immunodeficiency states (human immunodeficiency virus [HIV]-related, or immunosuppression for organ transplants or collagen-vascular disease); however, such second neoplasms usually show the presence of EBV by in situ hybridization for EBER-1 RNA, and we are not aware of any such cases of true histiocytic lineage. A third possibility for a causal relationship in these cases is that the THLs may represent transformation of the original LyL or ALL.

Hematologists, oncologists, and pathologists will benefit from the knowledge that THLs, documented using current techniques, may occur following otherwise successful treatment of lymphoblastic neoplasms. These are morphologically and immunophenotypically distinct from the lymphoblastic neoplasms and appear to display an aggressive clinical behavior. Additional studies of such cases will be needed to provide information on the possible mechanism of this phenomenon.

REFERENCES

1. Ralfkiaer E, Delsol G, O'Connor NTJ: Malignant lymphomas of true histiocytic origin. A clinical, histological, immunophenotypic and genotypic study. J Pathol 160:9, 1990
2. Levine EG, Hanson CA, Jaszcz W, Peterson BA: True histiocytic lymphoma. Semin Oncol 18:39, 1991
3. Hsu S, Ho Y, Hsu P: Lymphomas of true histiocytic origin: Expression of different phenotypes in so-called true histiocytic lymphoma and malignant histiocytosis. Am J Pathol 138:1389, 1991
4. van der Valk P, van Oostveen JW, V SH, van der Kwast TH, Melief CJM, Meijer CJL: Phenotypic and genotypic analysis of large-cell lymphomas, formerly classified as true histiocytic lymphoma: Identification of an unusual group of tumors. Leukemia Res 14:337, 1990
5. Hanson CA, Jaszca W, Kersey JH: True histiocytic lymphoma: Histopathologic immunophenotypic and genotypic analysis. Br J Haematol 73:187, 1989
6. Kamel OW, Gocke CD, Kell DL, Cleary ML, Warnke RA: True histiocytic lymphoma: A study of 12 cases based on current definition. Leuk Lymphoma 18:81, 1995
7. Milchgrub S, Kamel OW, Wiley E, Vuitch F, Cleary ML, Warnke RA: Malignant histiocytic neoplasms of the small intestine. Am J Surg Pathol 16:11, 1992
8. Wilson MS, Weiss LM, Gatter KC, Mason DY, Dorfman RF, Warnke RA: Malignant histiocytosis: A reassessment of cases previously reported in 1975 based upon paraffin section immunophenotyping studies. Cancer 66:530, 1990
9. Itoyama T, Sadamori N, Sasagawa I: A T-cell neoplasia showing clinicopathologic features of malignant histocytosis with novel chromosomal abnormalities and N-*ras* mutation. Cancer 67:2103, 1991
10. Risdall RJ, McKenna RW, Nesbit ME: Virus-associated hemophagocytic syndrome: A benign histiocytic proliferation distinct from malignant histiocytosis. Cancer 44:993, 1979
11. Ohyashiki JH, Ohyashiki K, Yoyama K: T-cell receptor gene rearrangement and its expression in human myeloid leukemia cell lines. Cancer Genet Cytogenet 37:193, 1989
12. Sheibani K, Wu A, Ben EJ, Stroup R, Rappaport H, Winberg C: Rearrangement of kappa-chain and T-cell receptor beta-chain genes in malignant lymphomas of "T-cell" phenotype. Am J Pathol 129:201, 1987
13. Yin JAL, Kumaran TO, Marsh GW, Rossiter M, Catovsky D: Complete recovery of histiocytic medullary reticulosis-like syndrome in a child with acute lymphoblastic leukemia. Cancer 51:200, 1983
14. van der Kwast TH, van Dongen JJM, Michiels JJ, Hooijkaas H, Kappers MC, Hagemeijer A: T-lymphoblastic lymphoma terminating as malignant histiocytosis with rearrangement of immunoglobulin heavy chain gene. Leukemia 5:78, 1991
15. Takasaki N, Kaneko Y, Maseki N, Sakurai M, Shimamura K, Takayama S: Hemophagocytic syndrome complicating T-cell acute lymphoblastic leukemia with a novel t(11;14)(p15;11) chromosome translocation. Cancer 59:424, 1987
16. O'Brien DJ, Child JA, Stark A, Lauder I, Bird CC: Concurrent T-cell lymphocytic lymphoma and malignant histiocytosis. Histopathology 9:977, 1985
17. Heaton A, Kahn LB: Acute lymphocytic leukaemia terminating in malignant histiocytosis. S Afr Med J 57:502, 1979

Downloaded from http://ashpublications.org/blood/article-pdf/87/12/5207/620054/5207.pdf by guest on 28 September 2023

18. Shreiner DP: Acute lymphoblastic leukemia terminating as histiocytic medullary reticulosis. JAMA 231:838, 1975

19. Starkie CM, Kenny MW, Mann JR, Cameron AH, Hill FGH: Histiocytic medullary reticulosis following acute lymphoblastic leukemia. Cancer 47:537, 1981

20. Rosner F, Grunwald HW: Association of T cell acute lymphoblastic leukemia and histiocytic medullary reticulosis. Am J Med 77:910, 1984

21. Griffin JD, Ellman L, Long JC, Dvorak AM: Development of a histiocytic medullary reticulosis-like syndrome during the course of acute lymphocytic leukemia. Am J Med 64:851, 1978

22. Skoog DP, Feagler JR: T cell acute lymphocytic leukemia terminating as malignant histiocytosis. Am J Med 64:678, 1978

23. Trubowitz S, Sobel H, Davis S: Null cell (non-T, non-B) acute lymphoblastic leukemia terminating as malignant histiocytosis. Am J Clin Pathol 73:725, 1979

24. Chen TK, Nesbit ME, McKenna R, Kersey JH: Histiocytic medullary reticulosis in acute lymphocytic leukemia of T cell origin. Am J Dis Child 130:1262, 1976

25. Karcher DS, Head DR, Mullins JD: Malignant histiocytosis occurring in patients with acute lymphocytic leukemia. Cancer 41:1967, 1978

26. Liang D-C, Chu ML, Shih C-c: Reactive histiocytosis in acute lymphoblastic leukemia and non-Hodgkin's lymphoma. Cancer 58:1289, 1986

27. Bindl JM, Warnke RA: Advantages of detecting monoclonal antibody binding to tissue sections with biotin and avidin reagents in Coplin jars. Am J Clin Pathol 85:490, 1986

28. Cleary ML, Chao J, Warnke R, Sklar J: Immunoglobulin gene rearrangement as a diagnostic criterion of B-cell lymphoma. Proc Natl Acad Sci USA 81:593, 1984

29. Weiss LM, Chen YY, Liu XF, Shibata D: Epstein-Barr virus and Hodgkin's disease: A correlative in situ hybridization and polymerase chain reaction study. Am J Pathol 139:1259, 1991

30. Warnke RA, Kim H, Dorfman RF: Malignant histiocytosis (histiocytic medullary reticulosis): Clinicopathologic study of 29 cases. Cancer 35:215, 1975

31. Esseltine DW, De Leeuw NKM, Berry GR: Malignant histiocytosis. Cancer 52:1904, 1983

32. Huhn D, Meister P: Malignant histiocytosis: morphologic and cytochemical findings. Cancer 42:1241, 1978

33. Lampert IA, Catovsky D, Bergier N: Malignant histiocytosis: a clinicopathologic study of 12 cases. Br J Haematol 40:65, 1978

34. Ducatman BS, Wick MR, Morgan TW, Banks PM, Pierre RV: Malignant histiocytosis: A clinical, histologic, and immunohistochemical study of 20 cases. Hum Pathol 15:368, 1984

35. Falini B, Pileri S, De Solas I, Martelli MF, Mason DY, Delsol G, Gatter KC, Fagioli M: Peripheral T-cell lymphoma associated with hemophagocytic syndrome. Blood 75:434, 1990

36. Delabie J, Vandenberghe E, Kennes C, Verhoef G, Foschini MP, Stul M, Cassiman JJ, De Wolf-Peeters C: Histiocyte-rich B-cell lymphoma: a distinct clinicopathologic entity possibly related to lymphocyte predominant Hodgkin's disease, paragranuloma subtype. Am J Surg Pathol 16:37, 1992

37. Jaffe ES, Costa J, Fauci AS: Malignant lymphoma and erythrophagocytosis simulating malignant histiocytosis. Am J Med 75:741, 1983

38. Chen R-L, Su I-J, Lin K-H, Lee S-H, Lin D-T, Chuu W-M, Lin K-S, Huang L-M, Lee C-Y: Fulminant childhood hemophagocytic syndrome mimicking histiocytic medullary reticulosis: An atypical form of Epstein-Barr virus infection. Am J Clin Pathol 96:171, 1991

39. Dolezal M, Kamel O, van de Rijn M, Cleary M, Sibley R, Warnke R: Virus-associated hemophagocytic syndrome characterized by clonal Epstein-Barr virus genome. Am J Clin Pathol 103:189, 1995

40. Look AT, Naegele RF, Callihan R, Herrod HG, Henle W: Fatal Epstein-Barr virus infection in a child with acute lymphoblastic leukemia in remission. Cancer Res 41:4280, 1981

41. Egeler RM, Neglia JP, Puccetti DM, Brennan CA, Nesbit ME: Association of Langerhans cell histiocytosis with malignant neoplasms. Cancer 71:865, 1993

42. McClain K, Gehrz R, Grierson H, Purtilo D, Filipovich A: Virus-associated histiocytic proliferations in children. Am J Pediatr Hematol Oncol 10:196, 1988

43. Cline MJ: Histiocytes and histiocytosis. Blood 84:2840, 1994

44. Nemes Z, Thomazy V: Diagnostic significance of histiocyte-related markers in malignant histiocytosis and true histiocytic lymphoma. Cancer 62:1970, 1988

45. Pileri S, Mazza P, Rivano MT, Martinelli G, Cavazzini G, Gobbi M, Taruscio D, Lauria F, Tura S: Malignant histiocytosis (true histiocytic lymphoma): Clinicopathological study of 25 cases. Histopathology 9:905, 1985

46. Roholl PJM, Kleyne J, Pijpers HW, van Unnik JAM: Comparative immunohistochemical investigation of markers for malignant histiocytes. Hum Pathol 16:1985

47. Roholl PJM, Kleyne J, Prins MEF, Hooijkaas H, Vroom TM, van Unnik JAM: Immunologic markers analysis of normal and malignant histiocytosis: A comparative study of monoclonal antibodies for diagnostic purposes. Am J Clin Pathol 89:187, 1988

48. Turner RR, Wood GS, Beckstead JH: Histiocytic malignancies: Morphologic, immunologic and enzymatic heterogeneity. Am J Surg Pathol 8:485, 1984

49. Carbone A, Gloghini A, Valli De Re BD, Tamaro P, Boiocchi M, Volpe R: Histopathologic, immunophenotypic, and genotypic analysis of Ki-1 anaplastic large cell lymphomas that express histiocyte-associated antigens. Cancer 66:2547, 1990

50. Pulford K, Rigney E, Micklem K, Jones M, Stross W, Gatter K, Mason D: KP1: A new monoclonal antibody that detects a monocyte/macrophage associated antigen in routinely processed tissue sections. J Clin Pathol 42:414, 1989

51. Carbone A, Gloghini A, Volpe R, Pinto A: KP1 (CD68)-positive large cell lymphomas: A histopathologic and immunophenotypic characterization of 12 cases. Hum Pathol 24:886, 1993

52. Banks PM, Metter J, Allred DC: Anaplastic large cell (Ki-1) lymphoma with histiocytic phenotype simulating carcinoma. Am J Clin Pathol 94:445, 1990

53. Foa R, Casorati G, Giubellino MC: Rearrangements of immunogobulin and T cell receptor b and g genes are associated with terminal deoxynucleotidyl transferase expression in acute myeloid leukemia. J Exp Med 165:879, 1987

54. Pelicci PG, Knowles DM, Dalla-Favera R: Lymphoid tumors displaying rearrangements of both immunoglobulin and T cell receptor genes. J Exp Med 162:1015, 1985

55. Williams L, Moscinski LC: Sterile transcription of immunoglobulin/T-cell receptor genes and other evidence of early lymphoid differentiation in acute myelogenous leukemia. Leukemia 7:1423, 1993

56. Weiss LM, Hu E, Wood GS, Moulds C, Cleary ML, Warnke R, Sklar J: Clonal rearrangements of T-cell receptor genes in mycosis fungoides and dermatopathic lymphadenopathy. N Engl J Med 313:539, 1985

57. Kamesaki H, Koya M, Miwa H, Kita K, Doi S, Tatsumi E, Hatanaka M, Uchino H: Malignant histiocytosis with rearrangement of the heavy chain gene and evidence of monocyte-macrophage lineage. Cancer 62:1306, 1988

58. Winick N, McKenna R, Shuster J: Secondary acute myeloid leukemia in children with acute lymphoblastic leukemia treated with etoposide. J Clin Oncol 11:209, 1993

Downloaded from http://ashpublications.org/blood/article-pdf/87/12/5207/620054/5207.pdf by guest on 28 September 2023
