# Supplementary material for: An 8-year-old girl with secondary histiocytic sarcoma with BRAFV600 mutation following T-cell acute lymphoblastic leukemia demonstrating stable disease for 3 years on dabrafenib and trametinib – a case report and literature review
Source: BMC Pediatr. 2025 Mar 8;25:178. doi: 10.1186/s12887-025-05539-2 (PMC11889787; doi:10.1186/s12887-025-05539-2)
Supplement: Supplementary file 11 — Supplementary Material 11 [file 12887_2025_5539_MOESM11_ESM.pdf]

Pediatric and Developmental Pathology 13, 225–237, 2010
DOI: 10.2350/09-03-0622-OA.1
© 2010 Society for Pediatric Pathology

Clinicopathologic Features of Histiocytic Lesions Following ALL, with a Review of the Literature

EUMENIA C.C. CASTRO,[1] CRISTINA BLAZQUEZ,[2] JAIME BOYD,[3] HERNÁN CORREA,[4] J-P. DE CHADAREVIAN,[5] RAYMOND E. FELGAR,[6] NICOLE GRAF,[7] NORMAN LEVY,[8] ERIC J. LOWE,[9] JOHN T. MANNING JR.,[10] MARIA A. PROYTCHEVA,[11] CHRISTOF SENGER,[12] KATAYOON SHAYAN,[13] JAROSLAV STERBA,[14] ALICE WERNER,[15] URVASHI SURTI,[16] AND RONALD JAFFE[1*]

[1]Department of Pathology, Children's Hospital of Pittsburgh, Pittsburgh, PA, USA
[2]Department of Hematology, Hospital de Jerez, Cadiz, Spain
[3]Pediatra Hematólogo/Oncólogo, Consultorios Royal Center, Apartado, Panamá
[4]Division of Pediatric Pathology, Monroe Carell Jr. Children's Hospital, Nashville, TN, USA
[5]Department of Pathology, St Christopher's Hospital for Children, Philadelphia, PA, USA
[6]Department of Pathology, Division of Hematopathology UPMC-Presbyterian, Pittsburgh, PA, USA
[7]Histopathology Department, The Children's Hospital at Westmead, Sydney, Australia
[8]Department of Pathology, Dartmouth-Hitchcock Medical Center, Lebanon, NH, USA
[9]Children's Cancer and Blood Disorders Center, Children's Hospital of the King's Daughters, Norfolk, VA, USA
[10]Department of Hematopathology, Division of Pathology and Laboratory Medicine, Houston, TX, USA
[11]Department of Pathology and Laboratory Medicine, Children's Memorial Hospital, Chicago, IL, USA
[12]Department of Anatomic Pathology, British Columbia's Children's Hospital, Vancouver, BC, Canada
[13]Department of Pathology, Rady Children's Hospital San Diego, San Diego, CA, USA
[14]Pediatric Oncology Department, University Hospital Brno, Medical Faculty Masaryk, University Brno, Czech Republic
[15]Department of Pathology, Children's Hospital of The King's Daughters, Norfolk, VA, USA
[16]Pittsburgh Cytogenetics Laboratory, University of Pittsburgh, School of Medicine, Magee-Womens Hospital, Pittsburgh, PA, USA

Received March 25, 2009; accepted June 30, 2009; published online July 30, 2009.

ABSTRACT

We describe the clinicopathologic features of 15 patients who had histiocytic lesions that followed acute lymphoblastic leukemia (ALL). Twenty-one separate histiocytic lesions were evaluated that covered a wide spectrum, some conforming to the usual categories of juvenile xanthogranulomas (5), Langerhans' cell histiocytosis (1), Langerhans' cell sarcoma (4), Rosai-Dorfman disease (1), and histiocytic sarcoma (4). Most were atypical for the category by histology, phenotype, or abnormally high turnover rate. Seven low-grade lesions defied easy categorization and were characterized only as ''atypical histiocytic lesion'' following ALL. For those evaluated, the molecular signature of the prior leukemia was present in the histiocytic lesion. In 3 of 15 patients, the leukemia and histiocytic lesion shared immunoglobulin H or monoclonal TCR gene rearrangements and, in 4 of 15 patients, clonal identity was documented by fluorescence in situ hybridization. Four patients died of progressive disease, 3 of whom had histiocytic sarcoma and 1 who had an atypical lesion. One patient died of recurrent ALL. The other 10 patients are alive, 7 after recurrences and treatment with surgery and/or chemotherapy. The post-ALL lesions are more aggressive than their native counterparts, but despite the demonstration of the presence of the leukemia signature in 7 of 15 patients, the prognosis is generally favorable, except for patients with histiocytic sarcoma. It remains unclear whether the histiocytic lesions arise as a line from the original ALL or whether transdifferentiation is involved.

Key words: acute lymphoblastic leukemia, atypical histiocytosis, histiocytes

INTRODUCTION

Histiocytic lesions can express a wide gamut of biological behavior, from the common dermal ''histiocytomas'' to the systemic and unpredictable ''histiocytoses'' and a malignant form, histiocytic sarcoma. The literature contains examples of histiocytic lesions of all categories that follow acute lymphoblastic leukemia (ALL); the lesions are often difficult to characterize according to histopathologic and phenotypic criteria and commonly

Presented in abstract form at the Society for Pediatric Pathology Interim Meeting, Louisville, KY, October, 2008. Pediatr Dev Pathol 2008;11:493–494.

This work was supported by the Marjory Harmer Endowment.

**Corresponding author:* email: Ronald.Jaffe@chp.edu

Table 1. Specifications of the antibodies used in the study

Antibody	Clone	Company	Dillution	Retrieval
CD1a	O10	Immunotech (Marseille, France)	Prediluted	CC1 mild 30″
CD14	7	Vector (Burlingame, VT, USA)	1/10	CC1 mild 30″
CD68	PGM-1	DAKO (Denmark)	1/100	CC1 mild 30″
CD68	KP-1	Ventana (Tuscon, AZ, USA)	Prediluted	CC1 mild 30″
CD163	10D6	Vector (Newcastle, UK)	1/250	CC1 mild 30″
S100	Polyclonal	Novacastra (Newcastle, UK),	1/500	None
Langerin	12D6	Vector (Newcastle, UK)	1/100	CC1 mild 30″
Factor 13a	Polyclonal	Genetex, Inc. (San Antonio, TX, USA)	1/25	Protease digestion 4″
Fascin	55K-2	DAKO (Carpinteria, CA, USA)	1/500	CC1 mild 30″
Ki-67	MIB-1	DAKO (Carpinteria, CA, USA)	1/25	CC1 mild 30″

CC1 indicates cell conditioning 1, which is antigen retrieval from Ventana Benchmark, similar to high pH EDTA buffer.

behave in an aggressive fashion [1–8]. In some instances, the histiocytic lesions have been shown to share molecular genetic or cytogenetic features with the original leukemia [3,4,7,9]. This curious phenomenon raises questions about the derivation of the histiocytes, the biological nature of a lesion that harbors a neoplastic marker, and whether there are histopathologic features that might be important in diagnosis and prognosis.

We describe 22 lesions from 15 patients who have had histiocytic lesions reviewed in consultation following an episode of ALL. We describe the clinical features of the histiocytic lesions, their histopathology, cytology, and phenotype, and relate them to their molecular genetic or cytogenetic content. Treatment, outcome, and diagnostic features are reviewed.

MATERIALS AND METHODS

Case selection

We reviewed material sent in consultation (R.J.), usually as tissue blocks or unstained slides, from 15 patients who had a history of ALL. In 3 instances, 2 separate lesions were reviewed that were temporally distinct, and 4 local recurrences were reviewed from one patient.

For the review, institutional privacy criteria were met at the submitting institutions.

Histopathology and immunophenotype

Slides were stained for hematoxylin and eosin and for a panel of histiocytic markers, as noted in Table 1. Staining done in earlier years was manual and antigen retrieval was most commonly performed by steamer. For the majority of cases that are of more recent vintage, staining was done on a Ventana Benchmark XT automatic slide stainer (Ventana, Tucson, AZ, USA) (Table 1).

CD14/CD68-PGM-1/CD163 is used as a generic macrophage phenotype. S100/CD1a/Langerin is the Langerhans' cell phenotype. The juvenile xanthogranuloma (JXG) family of lesions has the macrophage phenotype and, in addition, expresses Factor 13a and Fascin, but, in most instances, not S100. The Rosai-Dorfman type has a typical R-D cell with abundant cytoplasm, a large pale nucleus, and the macrophage phenotype, as well as S100/fascin. The reticulohistiocytoma or epithelioid histiocytoma has a very large cytoplasm-rich cell with light PAS-positivity and the macrophage phenotype, but without S100 or fascin [10].

In the new 4th edition of the *World Health Organization Volume on Classification of Tumors of Haematopoietic and Lymphoid Tissues*, there is no mention of a separate group of conditions that would encompass the post-ALL lesions, although the individual diagnostic entities are represented [11].

Lesions were labeled ''atypical'' when they were of cytologic low-grade but failed to fit into one of the above categories or contained a mix of features, and/or had inappropriately high nuclear pleomorphism, mitoses, and Ki-67 proliferation marker higher than 10%. Ki-67 was scored by using the histiocyte markers to identify the histiocytes, then counting 200 histiocyte nuclei and the number with Ki-67 staining, expressed as %. The typical histiocytic lesions, with few exceptions, should not have a Ki-67 higher than 10%; this was defined as the upper limit of ''normal'' proliferation (R.J., unpublished).

Clinical follow-up and molecular studies

Follow-up clinical information and, whenever available, molecular genetic/cytogenetic information, were provided by the contributors. Classical cytogenetic analysis was done on the original ALL and compared with the results of fluorescence in situ hybridization on interphase nuclei of the paraffin-embedded histiocytic lesions. During bone marrow remission, normal karyotype was demonstrated by G-banding, and no trisomies or constitutional abnormalities were demonstrated. Clonal T-cell receptor gene rearrangements and immunoglobulin H (IgH) gene rearrangements were proven by polymerase chain reaction (PCR) at the submitting institutions. In one case, the PCR results have been published [3].

Table 2. **Demographic distribution of the post–acute lymphocytic leukemia histiocytic lesions ($n = 21$)**

Patient #	Sex	Age	Leukemia Type	Interval	Diagnosis
1	M	2	T	1 y	Atypical Rosai-Dorfman disease
2	M	5	T	6 mo	Histiocytic sarcoma
3	M	15	B	3 mo	Histiocytic sarcoma
4	M	8	B	4 y	Atypical juvenile xanthogranuloma
5	M	7	B	1 y	Atypical juvenile xanthogranuloma
6a	F	13	B	11 y	Atypical juvenile xanthogranuloma
6b	F	14	B	12 y	Atypical juvenile xanthogranuloma
7	M	7	B	6 mo	Histiocytic sarcoma
8a	M	66	B	10 mo	Atypical adult xanthogranuloma
8b	M	66	B	17 mo	Atypical reticulohistiocytic lesion
9a	M	8	T	2 y	Langerhans' cell histiocytosis
9b	M	9	T	3 y	Langerhans' cell sarcoma
9c	M	10	T	4 y	Langerhans' cell sarcoma
9d	M	12	T	6 y	Langerhans' cell sarcoma
9e	M	13	T	7 y	Langerhans' cell sarcoma
10a	M	3	T	16 mo	Histiocytic sarcoma
10b	M	3	T	17 mo	Atypical histiocytic lesion
11	M	66	B	18 mo	Atypical histiocytic lesion
12	M	18	B	16 mo	Atypical histiocytic lesion
13	F	8	T	17 mo	Atypical histiocytic lesion
14	M	7	T	3 mo	Atypical histiocytic lesion
15	M	7	T	3 y	Atypical histiocytic lesion

RESULTS

Clinical findings

The sampling is skewed by virtue of referral pattern to a pediatric pathologist, accounting for 13 children, ages 2–18 years, and 2 adults, both 66 years old (Table 2). All of the patients were in ALL remission when they were diagnosed with the histiocytic lesion. There were 7 T-cell and 6 pre-B-cell pediatric ALLs. The interval between onset of the leukemia and the appearance of the histiocytic lesion was 3 months to 10 years, with a mean of 14.4 months when the single 10-year outlier was excluded. The interval in both adults was 12–18 months. From these 15 patients we studied 22 histiocytic lesions. Eight of the 19 pediatric histiocytic lesions were diagnosed as ''high-grade,'' that is, sarcomas, and 11 as low-grade, or ''atypical.'' The 3 histiocytic lesions from the 2 adults were ''low-grade/atypical'' in appearance.

Histopathologic findings

Although Langerhans' cell histiocytosis, JXG family, Rosai-Dorfman disease, and reticulohistiocytoma types were recognized in this group of cases, most lesions had features that were ''atypical'' by histopathologic, cytologic, or phenotypic features.

The JXG group included lesions at unusual sites such as lymph node, bone, and deep soft tissue, as well as the common skin site. The JXGs had a dominant population of medium-sized oval histiocytes, often xanthomatous, with interspersed Touton cells in most. The nuclei were oval, without the extensive folding of the Langerhans' cell histiocytosis (LCH) nucleus. The phenotype was CD14/CD68/CD163/F13a/Fascin but with low or absent S100, CD1a, and Langerin. What was atypical for a JXG was the degree of nuclear pleomorphism and, in some, an MIB-1 index of up to 30% (Table 3).

The Rosai-Dorfman lesion in the skin had clusters of large cells with a very pale cytoplasm that contained emperipolesis. The cells had the macrophage phenotype CD14/CD68/CD163, but the cytoplasm also stained strongly for S100 and Fascin. F13a/CD1a/Langerin was not detectable. The Rosai-Dorfman lesion was atypical only by virtue of the high MIB-1 content of the large cells, up to 30%. Two bland dermal JXGs were removed from this child in the clinical follow-up (#1).

The reticulohistiocytoma (epithelioid histiocytoma) was removed from the skin of an adult 7 months after an atypical JXG-type lesion at a different site (#8) was excised. The lesion had giant but mostly uninucleated histiocytes with abundant glassy cytoplasm, without emperipolesis and light PAS-positivity. These cells have smaller nuclei than the Rosai-Dorfman cell. The cells had the macrophage phenotype, CD14/CD68/CD163 without S100 but some F13a was present and the lesion was hemosiderotic. The Ki-67 count was less than 10%.

The Langerhans' cell lesions were seen in a single patient and have been previously reported [3] but were independently reviewed as a series here. The original LCH lesion of the skin was not diagnosable as a sarcoma even in retrospect, despite containing the monoclonal TCRγ gene rearrangement of the preceding ALL. Successive recurrences at the site, however, became progressively more anaplastic and included large, bizarre, atypical multipolar mitoses, and the Ki-67 index rose

Table 3. Histologic and immunohistochemical characterization of the histiocytic lesions

Pt. #	Diagnosis	CD14	CD68*	CD163	F13a	Fascin	S100	CD1a	Langerin	Ki-67
1	Atypical Rosai-Dorfman	+++	+++	+++	0	+++	+++**	0	0	30%
2	Histiocytic sarcoma	+++	+++	+++	+	+++	+	N/D	N/D	50%
3	Histiocytic sarcoma	+++	+++	+++	+	+	+	N/D	N/D	30%
4	Atypical JXG	+++	+++	+++	0	+++	+	0	N/D	30%
5	Atypical JXG	+++	+++	+++	++	N/D	0	0	0	30%
6a	Atypical JXG	+++	+++	+++	0	+++	0	N/D	N/D	5%
6b	Atypical JXG	+++	+++	+++	++	+++	0	N/D	N/D	1%
7	Histiocytic sarcoma	++	++	++	N/D	+++	+	N/D	N/D	50%
8a	Atypical JXG	+++	+++	+++	++	++	+	0	0	5%
8b	Atypical reticulohistiocytic lesion	+++	+++	+++	++	++	0	0	0	1%
9a	Langerhans' cell histiocytosis	N/D	N/D	N/D	N/D	N/D	+++	+++	+++	15%
9b	Langerhans' cell sarcoma	N/D	N/D	N/D	N/D	N/D	+++	+++	+++	50%
9c	Langerhans' cell sarcoma	N/D	N/D	N/D	N/D	N/D	+++	+++	+++	80%
9d	Langerhans' cell sarcoma	N/D	N/D	N/D	N/D	N/D	+++	+++	++	80%
10	Histiocytic sarcoma	+	+++	+++	+/−	N/D	0	0	0	80%
10	Atypical histiocytic lesion	N/D	+++	+++	0	++	0	N/D	N/D	1%
11	Atypical histiocytic lesion	+++	+++	+++	0	+++	0	0	N/D	50%
12	Atypical histiocytic lesion	+++	+++	+++	0	0	0	0	N/D	5%
13	Atypical histiocytic lesion	+++	+++	+++	0/+	+++	0/+++***	0	N/D	10%
14	Atypical histiocytic lesion	+++	+++	+++	+++	+++	+	0	0	N/D
15	Atypical histiocytic lesion	+++	+++	+++	+	+++	+++	0	0	N/D

*PGM 1, **S100 positive in the Rosai-Dorfman–like cells, ***S100 positive in the giant cells. N/D indicates not done; +, percentage of positive cells; +, less than 10%; ++, between 10 and 50%; +++, more than 50%.

progressively. The phenotype was that of Langerhans cell lesions, CD1a/Langerin/S100, with loss of Langerin in later recurrences.

The noncategorizable ''atypical histiocytic lesions'' did not fit clearly into the above compartments because of a mix the various histiocytic cell types and prominent nuclear pleomorphism. The variable nuclear shape and size was accompanied by prominent nucleoli in some, with multinucleated giant cells, some with Touton-type wreath appearance. The cytoplasm varied with xanthomatous elements, epithelioid forms and some with Rosai-Dorfman type pale cytoplasm with emperipolesis. In most there was no dominant histiocyte cell type. The phenotype was also mixed, with most cells having CD14/CD68/CD163 macrophage phenotype, and some having light and variable staining for S100. Factor 13a and Fascin were sparsely represented. Ki-67 was generally high, with up to 30% of histiocytic cells revealing staining (Table 3).

Four cases had ''malignant'' features by virtue of blastic appearance of the histiocytes or anaplastic sarcomatous features with a Ki-67 of 30–80%. The sarcomas were cytologically high-grade and the diagnosis of histiocytic sarcoma was predicated on the histiocytic appearance and phenotype, CD14/CD68/CD163.

Clinical outcome

There was a wide spectrum of biological behavior, treatment, and outcome (Table 4). Some lesions, most notably those confined to skin, could be locally excised even without adjuvant treatment (#1, #8). Recurrence and the identification of IgH in the leukemia and subsequent lesions did not constitute ''malignancy'' and did not receive more aggressive treatment in this instance (#8). A deep soft tissue lesion was characterized as ''atypical JXG'' but had a low Ki-67 rate (5%) recurred locally and rapidly as an aggressive tumor encasing the ribs, again with a low turnover rate (#8) (Fig. 1). Surgery was the mainstay of treatment. Similarly, a local atypical histiocytic lesion that was partially excised continued to grow and required more radical surgery (#11). Some lesions that were not diagnosable as sarcomas on histopathological grounds did disseminate, and it was disconcerting that some of the disseminated lesions, such as those in the marrow, had a relatively bland and nonsarcomatous appearance (#5, #12, #13) (Fig. 2).

Some lesions had ''malignant'' features by virtue of blastic appearance of the histiocytes or anaplastic sarcomatous features. Three of these patients died with disseminated disease, despite chemotherapy (#2, #3, #10) (Fig. 3). One patient, with bone and kidney disease (both sites proven on biopsy) but without further dissemination, is alive following bone marrow transplantation and chemotherapy (#7), and one with disease confined to the skin is alive but without metastasis after chemotherapy and multiple surgical excisions (#9). The relationship between the histopathologic appearance of the lesions and outcome holds broadly in that the sarcomas have a tendency to spread and may be lethal while the more prototypical histiocytic lesions remain localized. Importantly though, the ''atypical'' lesions have recurred locally (#6, #8, #11) or disseminated and required chemotherapy (#5, #12, #13).

Table 4. Location and treatment of the lesions and outcome of the patients

Pt. #	Site	Treatment	Recurrence/outcome
1	Skin	Excision	Two benign JXGs at other sites; on clinical follow-up
2	Multiple bones	Chemotherapy	Died of disease
3	Soft tissue	Chemotherapy	Chemotherapy was discontinued due to multiple recurrences in bone, lung, and soft tissue; presumed dead
4	Lymph node	Excision	No follow-up
5	Multiple bones and lung	Chemotherapy and bone marrow transplant	Free of disease after bone marrow transplant
6	Rib	Excision	Recurrence of the first lesion; now free of disease
7	Femur	Chemotherapy	On treatment
8	Skin	Excision	Recurrence as an atypical reticulohistiocytic lesion restricted to the skin
9	Skin, Testis	Radical excision and chemotherapy	Multiple recurrences as a Langerhans' cell sarcoma; metastatic disease after treatment
10	Liver and small bowel	Chemotherapy	Died of disease
11	Soft tissue	Excision	On treatment with local therapy including radiation; currently well, with no evidence of disease elsewhere
12	Bone marrow/liver/skin	Chemotherapy	Pancytopenia and hepatorenal dysfunction; died of disease
13	Multiple bones	Chemotherapy	Multiple lesions in soft tissue and bone Partial regression after chemotherapy
14	Multiple bones and bone marrow	Chemotherapy	Patient had a relapse of the leukemia and died
15	Intraperitoneal lesions	Chemotherapy, allogeneic blood stem cell transplant, and multiple organ transplant	Patient was last seen 5 years after the allogenic blood stem cell transplant and was asymptomatic and in remission at that time

JXGs indicates juvenile xanthogranulomas.

Lesions within a single patient could vary. Patient #10, who had multiple sites of involvement, had a high-grade anaplastic sarcoma in the liver with a Ki-67 count of 50%, but a low-grade lesion of the intestine without cytologic atypia and a Ki-67 index of only 1% (Fig. 4). This variability in a given patient may make diagnosis and prognostication difficult when low-grade elements are found at some sites, like the marrow (#12). One patient, previously reported [12], had a prototypical Langerhans' cell histiocytosis of the skin with a Ki-67 count of 15% that progressed though 4 local recurrences to a high-grade, anaplastic appearance with a Ki-67 index of 80% (#9) (Fig. 5). Despite the anaplastic appearance, the patient is alive, but with metastatic disease, 10 years after the first local presentation.

Molecular studies findings

The molecular/genetic relationship between the original leukemia and the subsequent histiocytic lesion was established for 7 of the 15 patients. The remaining 8 patients did not have material available for molecular/genetic studies, which is not unusual for a consultation practice (Table 5). Seven of the patients demonstrated clonal identity between the primary leukemia and the histiocytic lesion. For 3 patients, both the leukemia and histiocytic lesions shared IgH or clonal TCR gene rearrangements, a feature reported previously to occur in some ''histiocytic'' lesions [13]. B or T-cell gene rearrangements were found, but not B or T phenotypic lymphocyte markers. The outcome in these 3 patients varied: benign without further treatment (#8), multiple site sarcoma alive after treatment (#9), and localized sarcoma alive on treatment (#13). Of the remaining 4 patients, 3 had atypical lesions; 1 of these 3 patients died of disease (#12), 1 is alive on therapy (#11), and 1 had partial regression after therapy (#13). The fourth patient had histiocytic sarcoma and died of disease (#2).

DISCUSSION

We report 22 histiocytic lesions that presented in 15 patients following a diagnosis of ALL of T or B-cell type. The unifying thread in these lesions is the history of a prior leukemia and, in some, evidence that there is clonal identity between the leukemia and the subsequent histiocytic lesion. From the clinical and histopathologic point of view, there is a wide diversity of lesions and outcomes given the unique context, and the histopathology alone is insufficient to predict outcome in many.

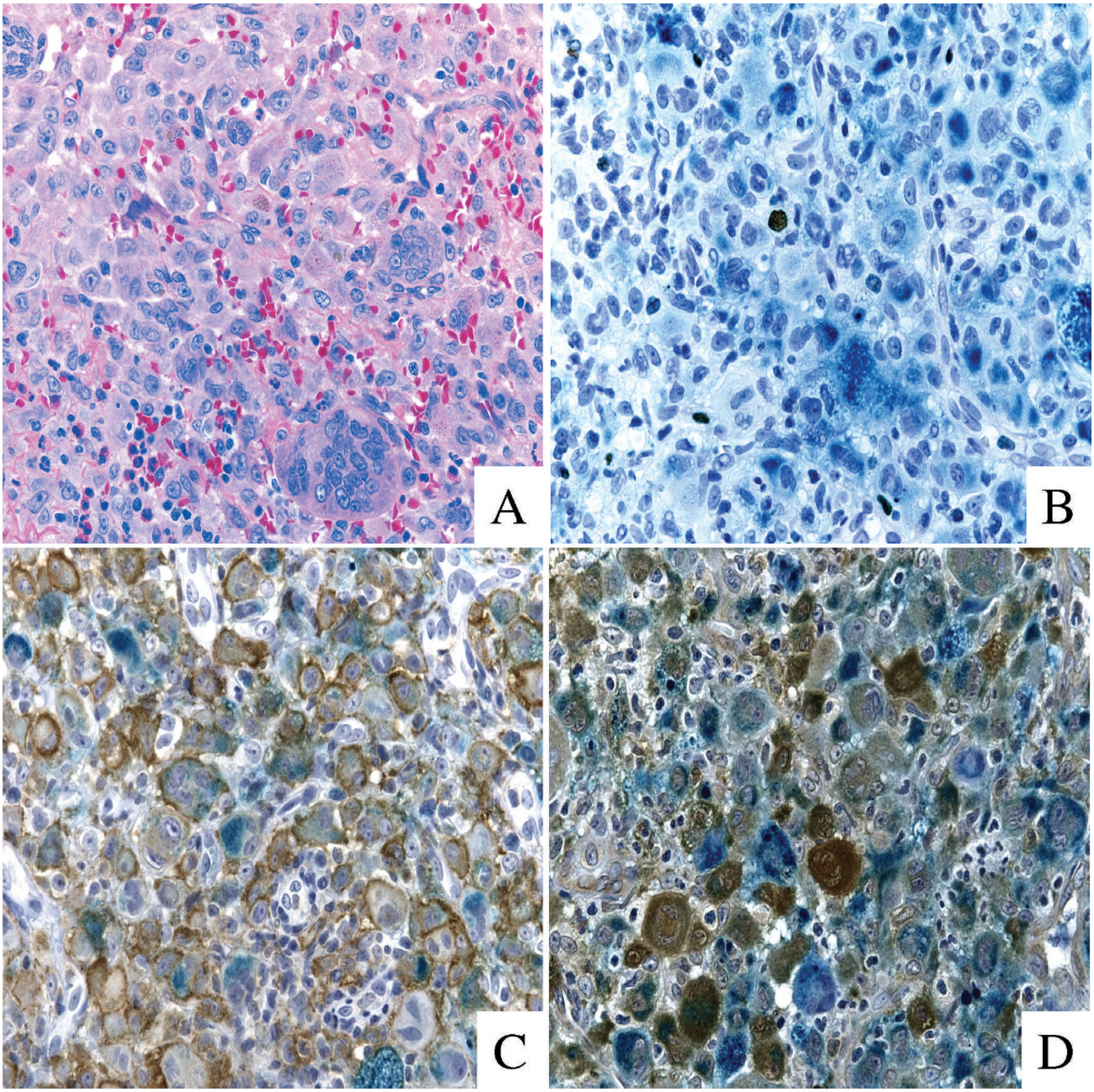

Figure 1. Atypical juvenile xanthogranuloma lesion in the skin, low grade, clinically recurrent. **A.** The hematoxylin and eosin stain reveals a bland but pleomorphic population that includes Touton-like cells (×40). **B.** Ki-67 confirms the minute turnover (×40, iron counterstain). **C.** Small and large cells express CD163 staining (×40, iron counterstain). **D.** Many of the cells express F13a and the high hemosiderin content is visible in blue (×40, iron counterstain).

The lesions fit broadly into the categories of Langerhans' cell disease, the JXG family of histiocytic disorders, Rosai-Dorfman disease, and reticulohistiocytoma (epithelioid histiocytoma). The malignant end of this spectrum is represented by histiocytic sarcoma (''true histiocytic lymphoma'') and Langerhans' cell sarcoma. Not all of the lesions are easily categorized; there are cytologic and phenotypic features that make the lesions ''atypical'' for the prototype and prevent stereotypical diagnostic labels. The cytologic atypia manifests with variable cell shape, size, presence of giant cell forms and, importantly, nuclear changes not usually seen in the prototypical lesions. There is often a mix of histiocytic cells more diverse than one would expect in LCH or JXG, leading to some overlap with the Rosai-Dorfman disease or reticulohistiocytoma types. More concerning was the wide disparity in nuclear shape and size, which was greater than that of the prototype and accompanied by evidence of higher cell turnover, mitoses, and Ki-67 index in some. The Ki-67 rate, by definition, was very high (30–50%) in the sarcomas, but was occasionally in the same range for the merely ''atypical'' proliferations (lesions/patients #1, #5, #9, #11).

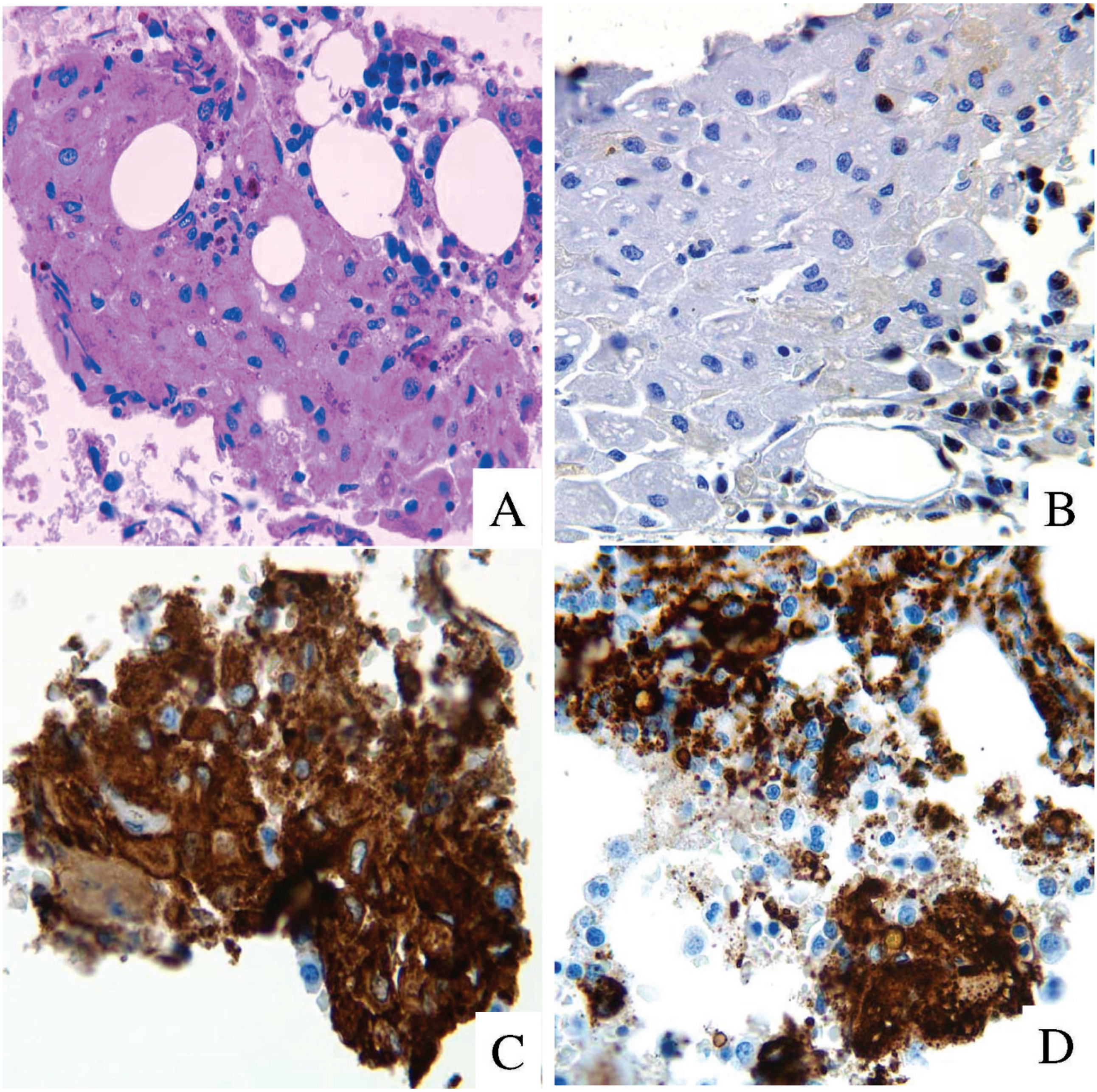

Figure 2. Low-grade atypical histiocytic lesion in the bone marrow, with poor outcome. **A.** The histiocytes are bland with small nuclei and abundant cytoplasm in this marrow aggregate (PAS, ×40). **B.** The Ki-67 index is minimal in the histiocytes (×40). **C.** The cells stain intensely for CD163 (×40). **D.** Clusters of large, bland macrophages stain strongly for CD68 (PGM-1 antibody, ×40).

The sarcomas were cytologically high grade and the diagnosis of histiocytic sarcoma was predicated on the histiocytic appearance and phenotype, CD14/CD68/CD163.

Earlier descriptions of the association of histiocytic lesions of the monocyte/macrophage variety and ALL are handicapped because ''histiocyte'' was the name given to the appearance of a high-grade lymphoma with large, vesicular nuclei, and there is no assurance that the ''anaplastic histiocytic lymphoma'' described by Dosik and colleagues [14] was histiocytic at all. Weiss and colleagues [13] documented that most of the lesions previously described as ''histiocytic'' were, in fact, T-cell in origin, and some of these have since been shown to be anaplastic large cell lymphomas. The suggestion was also made [13] that T-cell gene rearrangements might be a common feature of monocytic/histiocytic tumors and, more recently, that clonal Ig receptor gene rearrangements are frequent in sporadic histiocytic/dendritic cell sarcomas [15]. Kamesaki and colleagues [16] described malignant histiocytosis with monocyte-macrophage differentiation that had Ig heavy chain gene rearrangement. It was Hanson and colleagues [17] who ushered in the current use of the notion of a true histiocytic malignancy

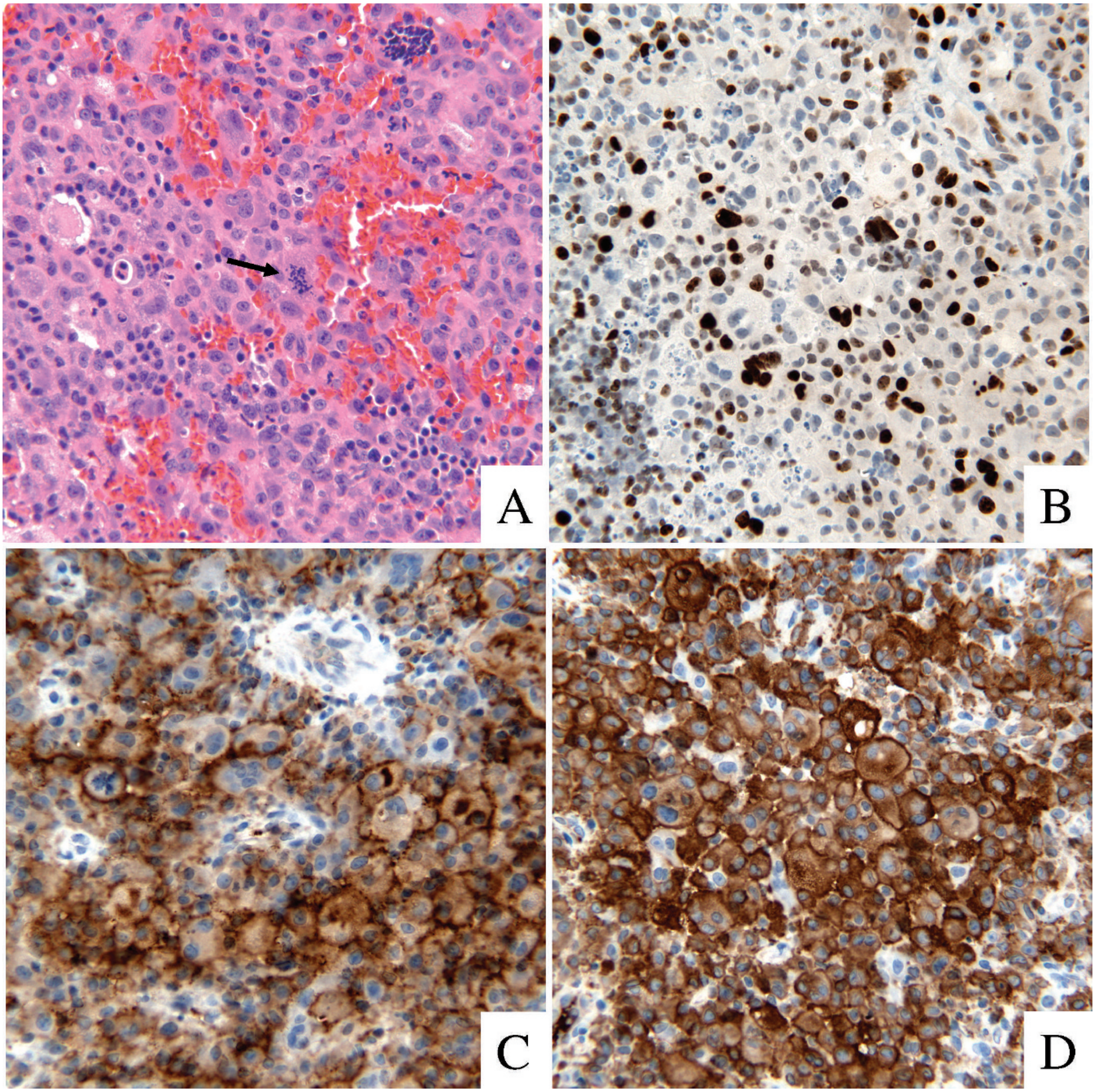

Figure 3. High-grade lesion in the bone, histiocytic sarcoma. **A.** The cells are recognizable as ''histiocytic,'' but there is marked nuclear pleomorphism with atypical mitosis (arrow) (hematoxylin and eosin, ×40). **B.** The Ki-67 marker is positive in almost half the recognizable histiocytes (×40). **C.** CD14 (and CD68) were strongly expressed (CD14, ×40). **D.** CD163 highlights surface and some cytoplasmic stain, confirming the macrophage nature of the lesion (×40).

based on the histopathology, immunophenotype, and genotypic contribution. Van der Kwast and colleagues [18] described a T-lymphoblastic lymphoma that terminated as monocytic-type leukemia with macrophage morphology that had rearrangement of the lg heavy chain gene. Lindh and colleagues [19] described a fatal monohistiocytic malignancy 6 years after a pre-B ALL in which both Ig and TCRγ clonal rearrangements were present in the second tumor. Soslow and colleagues [20] reported 3 instances of ''true histiocytic lymphoma,'' 1 with a T-cell gene rearrangement after a T-ALL and 2 without Ig gene rearrangement after B-ALL. Bouabdallah and colleagues [1] documented histiocytic lymphoma after a precursor B-ALL in which common clonal derivation of the IgH rearrangement was documented. Single cases of the association between ALL and later histiocytic lymphoma continued to accrue [13,21–26], including an example after T-ALL and following stem cell transplant that responded in part to thalidomide [27] after everything else had failed. Feldman and

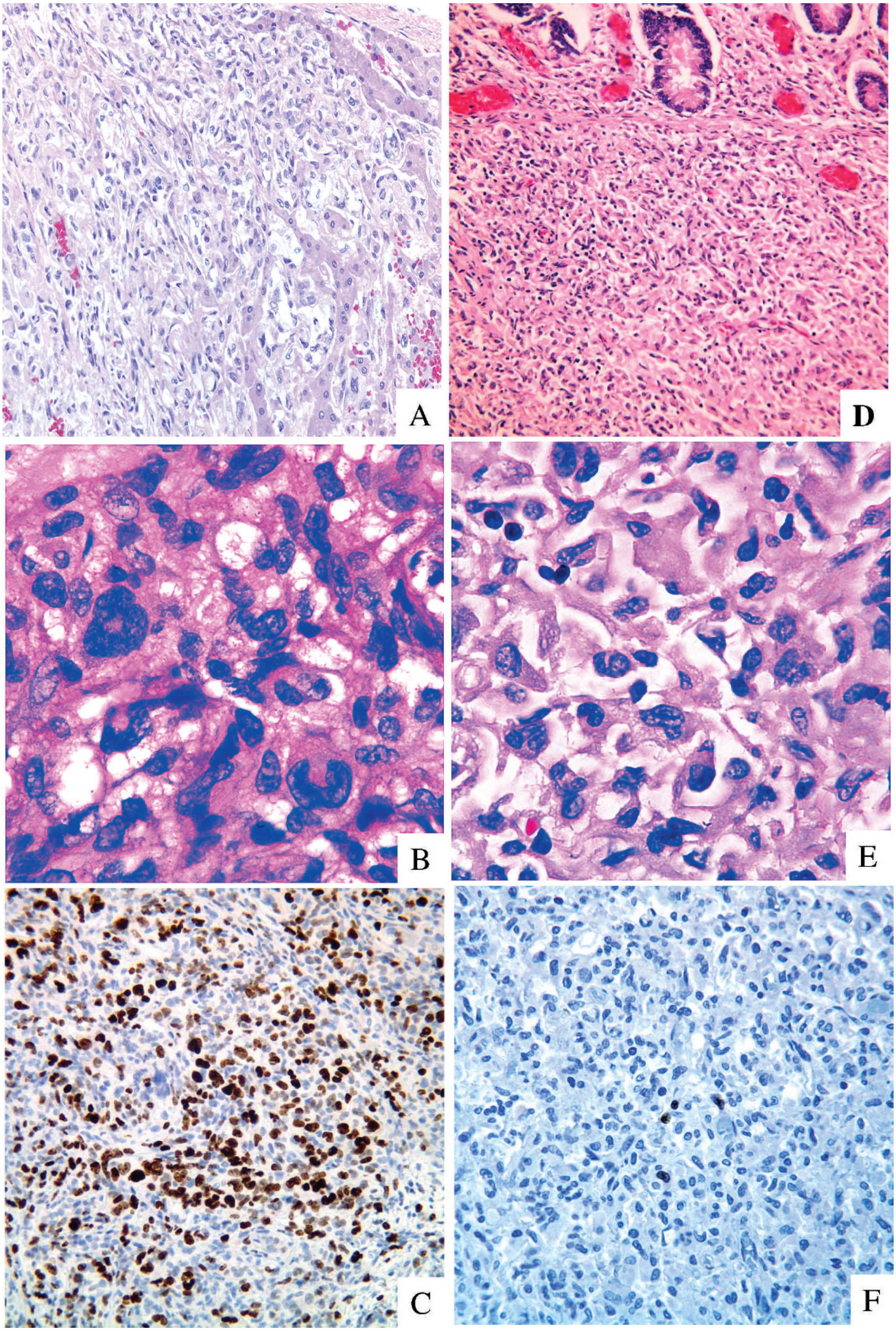

Figure 4. High-grade sarcoma and low-grade lesion in the same patient. **A**, **B**, and **C** (left) show a high-grade lesion in the liver; **D**, **E**, and **F** illustrate a lowgrade lesion in the bowel. **A.** Liver. Much of the liver is infiltrated in a sinusoidal pattern (hematoxylin and eosin [HE], ×20). **B.** The histiocytes are anaplastic, with bizarre nuclei, and atypical mitoses were abundant (HE, ×40). **C.** The Ki-67 content is high, in contrast with that in **F** (×20). **D.** Colonic nodule. A bland histiocytic process fills the submucosa and the lamina propria (HE, ×20). **E.** There is some nuclear pleomorphism without atypical mitoses (HE, ×40). **F.** The Ki-67 index is low by contrast (see **C**) (×20).

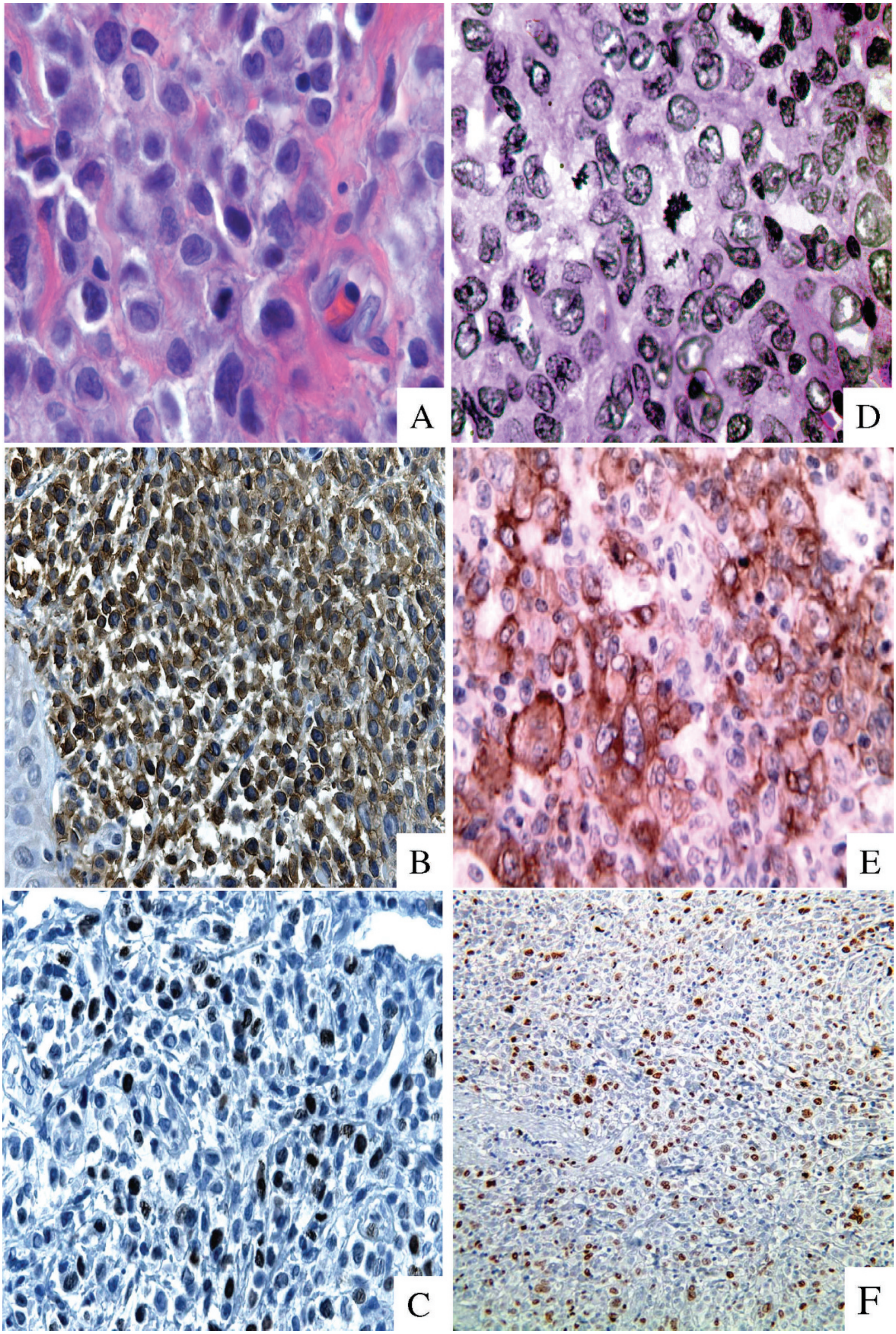

Figure 5. Langerhans cell sarcoma in the skin, progressive from low-grade Langerhans cell lesion. **A**, **B**, and **C** (left) are the initial, low-grade lesion, and **D**, **E**, and **F** (right) illustrate a later high-grade recurrence. **A.** Langerhans' cell lesion, Langerhans' cell histiocytosis, only minimal nuclear pleomorphism and mitoses (hematoxylin and eosin [HE], ×100). **B.** CD1a was strongly expressed on the cell surface and Langerin staining was uniformly positive (not shown) (×40). **C.** Ki-67 shows the prominent local turnover, 15% (×40). **D.** The high-grade recurrences had nuclear pleomorphism, mitosis, and atypical mitoses (HE, ×100). **E.** CD1a was still expressed on a high proportion of histiocytes (×40). **F.** Ki-67 mitotic rate of the CD1a positive cells by contrast (see **C**) was greater than 80% (×40).

Table 5. **Molecular and cytogenetic characterization of the lesions**

Patient #	Molecular characterization of the leukemia	Molecular characterization of the histiocytic lesion
1	N/D	N/D
2	t (7,11) p16 gene deletion on both chromosomes 9	FISH from the thymic lesion demonstrated that 100% of the nuclei had a p16 deletion on both chromosomes 9
3	N/D	N/D
4	N/D	N/D
5	46 XY, add (9)(p24),t(15;19)(p11.2;q12)[18]/46,XY[2]	N/D
6	N/D	N/D
6	N/D	N/D
7	46,XY, t(8;14)(q24;q32)[4]/47,idem,ti(1)(q10)[8]/46,xy[8].	FISH from the bone lesion demonstrated the t(8;14)
8	Immunoglobulin heavy chain rearrangement	Immunoglobulin heavy chain rearrangement
9	Monoclonal rearrangement of TCRγ^{13}	Monoclonal rearrangement of TCRγ^{14}
10	t (17;11)(p13;q13a)	N/D
11	Trisomy 11 by classical cytogenetics and confirmed using interphase FISH with a probe for the MLL gene locus on 11q23. There was no rearrangement of the MLL locus.	FISH of the soft tissue tumor demonstrated 3 signals for chromosome 11, using a probe for the MLL gene locus on 11q23
12	47, XY, +5, in 14 mitoses, then confirmed using FISH in 100% of cells	Trisomy 5 detected by FISH from the skin lesion
13	Monoclonal rearrangement of TCRγ	Monoclonal Rearrangement of TCRγ
14	46XY	N/D
15	N/D	N/D

FISH indicates fluorescent in-situ hybridizaton; N/D, not done.

colleagues [4] confirmed the clonal identity of a histiocytic sarcoma that occurred during maintenance therapy for pre B-ALL and identified identical IgH gene rearrangements in both. They also made the point that TCRγ gene rearrangements can be common in precursor B-ALL.

Onciu [6] described 2 cases presented at the 2003 SHP/EAHP workshop in which histiocytic sarcoma developed 3 months and 2 years after precursor B-cell ALL. Both histiocytic neoplasms contained the clonal gene rearrangement of the previous leukemia, IgH in one and IgH with TCRγ in the other. Szczepanski and colleagues [28] described juvenile myelomonocytic leukemia with monocyte/histiocyte skin lesions extending down to fat and that contained identical TCR gene and Ig rearrangements as the earlier leukemia. Trebo and colleagues [29], analyzing the outcome of 971 T-cell ALL patients treated on BFM-ALL protocols, identified 2 instances of histiocytic sarcoma, 2 of hemophagocytic syndrome, and 2 instances of Langerhans' cell histiocytosis. Borgman [30], reviewing 1376 patients with post-relapse non-B ALL, found one instance of a possible histiocytic sarcoma (or HLH). Feldman et al. [12] presented evidence for clonal trans-differentiation of adult follicular lymphoma to histiocytic or intervals up to 12 years following diagnosis. All 8 cases had t (14:18) IgH gene rearrangement or BCL2 gene breakpoints in the histiocytic sarcoma, suggesting lineage plasticity.

There is a well-described association between bone marrow disorders, usually myelomonocytic leukemia and the development of cutaneous JXG [31]. According to some authors, the association is much higher in the presence of NF1 [32], but others have disputed the strength of this association [33]. Aparicio and colleagues [34] described a child with B-cell ALL in relapse who developed cutaneous JXG over much of the body. No NF1 features were present. Shoo and colleagues [35] described an adult with skin and splenic xanthogranulomas following B-ALL and added one other from the literature that followed a T-ALL. NF1 was not mentioned in any of these instances. Five patients in our group developed lesions that followed B-cell ALL and had features of JXGs not limited to the skin. The lesions fit best into the JXG family by virtue of their histopathology and phenotype, being CD14/CD68/CD163/Factor 13a and fascin positive, but S100 and CD 1a-negative. They were classed as being ''atypical'' in that the degree of cellular pleomorphism and, in 2, the proliferative rate was more than that seen in standard JXGs. Three of these 5 lesions were large and involved bone and soft tissue.

It is evident that instances of histiocytic lesions, atypical proliferations, and histiocytic sarcomas have occurred after ALL and that molecular identity of the lesions can be expected.

The story with Langerhans' cell disease occurring after ALL is somewhat different, in part because the diagnosis is less labile and also because the LCH databases have been mined for associated lesions. Kanter and colleagues [21] described a mandibular LCH after ALL, but thought the association to be fortuitous. Egeler [2,36] and members of the Histiocyte Society found 5 instances of LCH following ALL within a year. They also made the point that most leukemias that followed LCH were acute myeloid leukemia and likely to be therapy-induced. Raj and colleagues [7] described LCH 2 years after completing therapy for ALL. Feldman and col-

leagues [3] described 2 instances of LCH, 1 synchronous and the other 2 years after the T-ALL. The patient with synchronous disease had progressive LCH after the leukemia therapy and both lesions harbored identical TCRγ rearrangements. Their second patient (who is our patient #9) had cytologic progression from a bland LCH-type lesion to a high-grade anaplastic appearance, multiply recurrent at a single site with late metasteses to testes. Once again, identity of the TCRγ rearrangement was documented. Trebo and colleagues [29], in reviewing the outcome of 971 patients with T-cell ALL treated with the BFM-ALL protocol, found 2 instances of LCH, both localized, and no data on clonal association were provided. Rodig and colleagues [37] described an instance of ''aggressive LCH'' with progression to fatal Langerhans' cell sarcoma following T-ALL, and harboring the same T-cell receptor gene rearrangement as well as 2 activating NOTCH1 mutations. Twenty-four instances of LCH and Rosai-Dorfman disease had no NOTCH1 mutations. There are reports of patients who presented with indeterminate cell or interdigitating cell tumors in the course of a previous or concurrent B-cell lymphoma [38–40]. The term ''indeterminate cell tumor'' indicates a dendritic cell tumor composed of Langerhans-type cells expressing CD1a and S100, but lacking Birbeck granules and Langerin [39]. In these instances, a t(14;18) was detected both in the histiocytic lesion and in the previous lymphoma [39]. The authors proposed that the histiocytic lesion was due to an underlying factor causing B-cell dedifferentiation.

The relationship between the ALL and the histiocytic lesions, especially the low-grade lesions, is not yet clear. Recent evidence in the mouse [41–43] provides for a common lymphoid-myeloid precursor that progresses to separate T-cell/myeloid and B-cell pathways. Common origin from a human T-cell/myeloid precursor might explain how the molecular signature is conserved, but the histiocytic element is amenable to varying degrees of differentiation. Other explanations, especially for histiocytic lesions that follow the B-ALL, require that there be lineage switching and greater amounts of plasticity rather than a shared precursor.

The lesions are rare enough that no standard approach to treatment is possible. Excision has been the initial approach for localized lesions, especially those in skin or soft tissues. A variety of chemotherapeutic regimens have been used for the more disseminated lesions and those of high grade. Four of 15 patients with adequate follow-up have died of progressive histiocytic disease.

REFERENCES

1. Bouabdallah R, Abena P, Chetaille B, et al. True histiocytic lymphoma following B-acute lymphoblastic leukaemia: case report with evidence for a common clonal origin in both neoplasms. Br J Haematol 2001;113:1047–1050.
2. Egeler RM, Neglia JP, Arico M, et al. The relation of Langerhans cell histiocytosis to acute leukemia, lymphomas, and other solid tumors. The LCH-Malignancy Study Group of the Histiocyte Society. Hematol Oncol Clin North Am 1998;12:369–378.
3. Feldman AL, Berthold F, Arceci R, et al. Clonal relationship between precursor T-lymphoblastic leukaemia/lymphoma and Langerhans' cell histiocytosis. Lancet Oncol 2005;6:435–437.
4. Feldman AL, Minniti C, Santi M, et al. Histiocytic sarcoma after acute lymphoblastic leukaemia: a common clonal origin. Lancet Oncol 2004;5:248–250.
5. Heaton A, Kahn LB. Acute lymphocytic leukemia terminating in malignant histiocytosis and literature review. A case report. S Afr Med J 1980;57:502–507.
6. Onciu M. Histiocytic proliferations in childhood. Am J Clin Pathol 2004;122:128–136.
7. Raj A, Bendon R, Moriarty T, et al. Langerhans cell histiocytosis following childhood acute lymphoblastic leukemia. Am J Hematol 2001; 68:284–286.
8. Van der Valk P, van Oostveen JW, Stel HV, et al. Phenotypic and genotypic analysis of large-cell lymphomas, formerly classified as true histiocytic lymphoma: identification of an unusual group of tumors. Leuk Res 1990;14:337–346.
9. Plowey ED, Felgar RE. Clonally-related Histiocytic Neoplasm in Patients with Prior Acute Lymphoblastic Leukemia. ASCP Check Sample Program: Clinical Hematology Series 2009;44:87–98.
10. Weitzman S, Jaffe R. Uncommon histiocytic disorders: the non-Langerhans cell histiocytoses. Pediatr Blood Cancer 2005;45:256–264.
11. Jaffe R, Pileri SA, Facchetti F, et al. Histiocytic and dendritic cell neoplasms. In: Swerdlow SH, Campo E, Harris NL. et al, eds. World Heath Organization Classification of Tumors of Haematopoietic and Lymphoid Tissues. Lyon, France: International Agency for Research on Cancer, 2008:353–366.
12. Feldman AL, Arber DA, Pittaluga S, et al. Clonally related follicular lymphomas and histiocytic/dendritic cell sarcomas: evidence for transdifferentiation of the follicular lymphoma clone. Blood 2008; 111:5418–5419.
13. Weiss LM, Trela MJ, Cleary ML, et al. Frequent immunoglobin and T-cell receptor gene rearrangements in histiocytic neoplasms. Am J Pathol 1985;121:369–373.
14. Dosik H, Anon R, Lee S, et al. Histiocytic lymphoma fifteen years following remission of acute lymphoblastic leukemia. Blood 1980; 55:944–945.
15. Chen W, Lau SK, Fong D, et al. High frquency of clonal immunoglobulin receptor gene rearrangements in sporadic histiocytic/dendritic cell sarcomas. Am J Surg Pathol 2009;33:863–873.
16. Kamesaki H, Koya M, Miwa H, et al. Malignant histiocytosis with rearrangement of the heavy chain gene and evidence of monocyte-macrophage lineage. Cancer 1988;62:1306–1309.
17. Hanson CA, Jaszcz W, Kersey JH, et al. True histiocytic lymphoma: histopathologic, immunophenotypic and genotypic analysis. Br J Haematol 1989;73:187–198.
18. van der Kwast TH, van Dongen JJ, Michiels JJ, et al. T-lymphoblastic lymphoma terminating as malignant histiocytosis with rearrangement of immunoglobulin heavy chain gene. Leukemia 1991;5:78–82.
19. Lindh J, Nordernson I, Osterman B, et al. Ig-gene and T-cell receptor gene rearrangements in a secondary, mono-histiocytic malignancy. Acta Oncol 1993;32:525–530.
20. Soslow RA, Davis RE, Warnke RA, et al. True histiocytic lymphoma following therapy for lymphoblastic neoplasms. Blood 1996;87:5207–5212.
21. Kanter HM, Lin LM, Goepp RA. Mandibular histiocytosis X and acute lymphoblastic leukemia. Oral Surg Oral Med Oral Pathol 1976;42:221–230.
22. Magni M, Di Nicola M, Carlo-Stella C, et al. Identical rearrangement of immunoglobulin heavy chain gene in neoplastic Langerhans cells and B-lymphocytes: evidence for a common precursor. Leuk Res 2002;26:1131–1133.
23. Martin Rodilla C, Fernandez Acenero J, Pena ML, et al. True histiocytic lymphoma as a second neoplasm in a follicular

centroblastic-centrocytic lymphoma. Pathol Res Pract 1997;193: 319–322.
24. Wetzler M, Kurzrock R, Goodacre AM, et al. Transformation of chronic lymphocytic leukemia to lymphoma of true histiocytic type. Cancer 1995;76:609–617.
25. Wongchanchailert M, Laosombat V. True histiocytic lymphoma following acute lymphoblastic leukemia. Med Pediatr Oncol 2003; 40:51–53.
26. Levine EG, Hanson CA, Jaszcz W, et al. True histiocytic lymphoma. Semin Oncol 1991;18:39–49.
27. Dalle JH, Leblond P, Decouvelaere A, et al. Efficacy of thalidomide in a child with histiocytic sarcoma following allogeneic bone marrow transplantation for T-ALL. Leukemia 2003;17:2056–2057.
28. Szczepanski T, de Vaan GA, Beishuizen A, et al. Acute lymphoblastic leukemia followed by a clonally-unrelated EBV-positive non-Hodgkin lymphoma and a clonally-related myelo-monocytic leukemia cutis. Pediatr Blood Cancer 2004;42: 343–349.
29. Trebo MM, Attarbaschi A, Mann G, et al. Histiocytosis following T-acute lymphoblastic leukemia: a BFM study. Leuk Lymphoma 2005;46:1735–1741.
30. Borgmann A, Zinn C, Hartmann R, et al. Secondary malignant neoplasms after intensive treatment of relapsed acute lymphoblastic leukaemia in childhood. Eur J Cancer 2008;44:257–268.
31. Cooper PH, Frierson HF, Kayne AL, et al. Association of juvenile xanthogranuloma with juvenile myeloid leukemia. Arch Dermatol 1984;120:371–375.
32. Zvulunov A, Barak Y, Metzker A. Juvenile xanthogranuloma, neurofibromatosis, and juvenile chronic myelogenous leukemia. World statistical analysis. Arch Dermatol 1995;131:904–908.
33. Cambiaghi S, Restano L, Caputo R. Juvenile xanthogranuloma associated with neurofibromatosis 1: 14 patients without evidence of hematologic malignancies. Pediatr Dermatol 2004;21:97–101.
34. Aparicio G, Mollet J, Bartralot R, et al. Eruptive juvenile xanthogranuloma associated with relapsing acute lymphoblastic leukemia. Pediatr Dermatol 2008;25:487–488.
35. Shoo BA, Shinkai K, McCalmont TH, et al. Xanthogranulomas associated with hematologic malignancy in adulthood. J Am Acad Dermatol 2008;59:488–493.
36. Egeler RM, Neglia JP, Arico M, et al. Acute leukemia in association with Langerhans cell histiocytosis. Med Pediatr Oncol 1994;23:81–85.
37. Rodig SJ, Payne EG, Degar BA, et al. Aggressive Langerhans cell histiocytosis following T-ALL: clonally related neoplasms with persistent expression of constitutively active NOTCH1. Am J Hematol. 2008;83:116–121.
38. Cossu A, Deiana A, Lissia A, et al. Synchronous interdigitating dendritic cell sarcoma and B-cell small lymphocytic lymphoma in a lymph node. Arch Pathol Lab Med 2006;130:544–547.
39. Rezk SA, Spagnolo DV, Brynes RK, et al. Indeterminate cell tumor: a rare dendritic neoplasm. Am J Surg Pathol 2008;32:1868–1876.
40. Vasef MA, Zaatari GS, Chan WC, et al. Dendritic cell tumors associated with low-grade B-cell malignancies. Report of three cases. Am J Clin Pathol 1995;104:696–701.
41. Bell JJ, Bhandoola A. The earliest thymic progenitors for T cells possess myeloid lineage potential. Nature 2008;452:764–767.
42. Graf T. Immunology: blood lines redrawn. Nature 2008;452:702–703.
43. Wada H, Masuda K, Satoh R, et al. Adult T-cell progenitors retain myeloid potential. Nature 2008;452:768–772.

Copyright of Pediatric & Developmental Pathology is the property of Allen Press Publishing Services Inc. and its content may not be copied or emailed to multiple sites or posted to a listserv without the copyright holder's express written permission. However, users may print, download, or email articles for individual use.
